# Conformational control enables boroxine-to-boronate cage metamorphosis†‡

**DOI:** 10.1039/d3sc02920d

**Published:** 2023-10-05

**Authors:** Manuel Rondelli, Samuel Delgado-Hernández, Antonio H. Daranas, Tomás Martín

**Affiliations:** a Instituto de Productos Naturales y Agrobiología, Consejo Superior de Investigaciones Científicas (IPNA-CSIC) Avda. Astrofísico Francisco Sánchez, 3 38206 La Laguna Tenerife Spain tmartin@ipna.csic.es; b Doctoral and Postgraduate School, University of La Laguna (ULL) 38200 La Laguna Tenerife Spain; c Departamento de Química, Unidad Departamental de Química Analítica, Universidad de La Laguna (ULL) 38206 La Laguna Tenerife Spain; d Instituto Universitario de Bio-Orgánica “Antonio González”, ULL Avda. Astrofísico Francisco Sánchez, 2 38206 La Laguna Tenerife Spain

## Abstract

The discovery of molecular organic cages (MOCs) is inhibited by the limited organic-chemical space of the building blocks designed to fulfill strict geometric requirements for efficient assembly. Using intramolecular attractive or repulsive non-covalent interactions to control the conformation of flexible systems can effectively augment the variety of building blocks, ultimately facilitating the exploration of new MOCs. In this study, we introduce a set of boronic acid tripods that were designed using rational design principles. Conformational control was induced by extending the tripod's arms by a 2,3-dimethylbenzene unit, leading to the efficient formation of a tetrapodal nanometer-sized boroxine cage. The new building block's versatility was demonstrated by performing cage metamorphosis upon adding an aromatic tetraol. This led to a quantitative boroxine-to-boronate transformation and a topological shift from tetrahedral to trigonal bipyramidal.

## Abstract 2

The conformational control of boronic acid building blocks allows efficient self-assembly into a tetrahedral boroxine cage. The nano-sized cage displays molecular metamorphosis, converting from a boroxine tetrapod to a boronate bipyramid.

## Graphical abstract

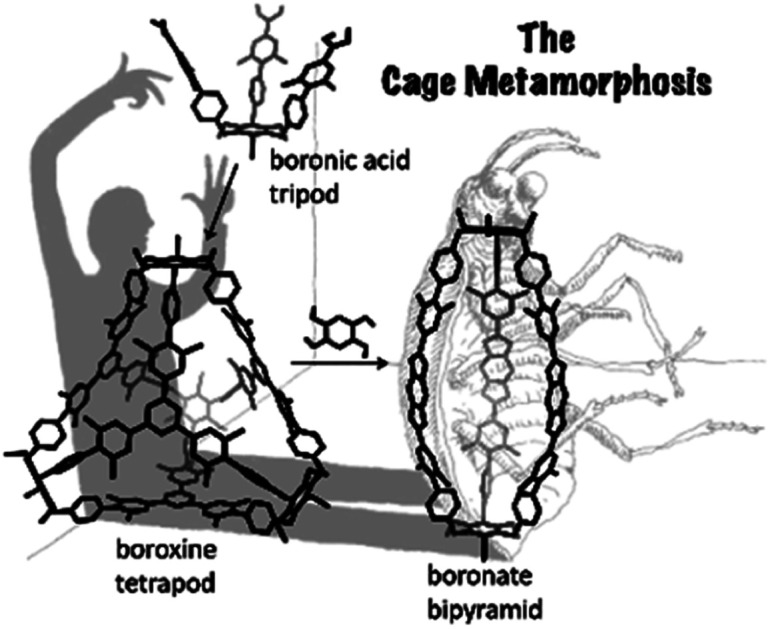

## Introduction

Covalent organic frameworks (COFs) have emerged as a groundbreaking class of porous materials with diverse applications, ranging from gas storage and separation to electronics and catalysis.^1^ In parallel, their molecular counterparts, molecular organic cages (MOCs), exhibit unique advantages over COFs, notably intrinsic porosity and solubility. These features enable convenient post-synthetic modifications and facilitate the crystallization process, leading to the formation of well-organized solid structures. Despite these promising attributes, the practical utilization of MOCs is still in its early stages, primarily due to the inherent difficulties associated with constructing such intricate architectures.

The general strategy for the design of these structures is based on a heuristic method known as the directional bonding approach.^2–5^ This approach implies that only the building blocks' geometry, directionality, and topicity are considered. The assembly of the building blocks is carried out using dynamic covalent chemistry (DCC), which enables error correction through reversibility, allowing access to stable covalent structures in high yields. However, the stability of the products is paid for with kinetic trapping, undesired side-products and unpredicted reaction outcomes. To minimize this, the building blocks are chosen to be highly rigid and close to the ideal geometry. However, two main limitations relate to this: (a) the ideal geometry cannot always be achieved due to the limitations of the organic-chemical space, and (b) rigid structures are overly sensitive towards geometric mismatches, preventing their adaptability to ideal geometries. As a result, the number of building blocks that can efficiently assemble MOCs is severely limited. Using intramolecular attractive or repulsive non-covalent interactions to control the conformation of flexible systems can effectively augment the variety of building blocks, thereby facilitating the exploration of new molecular organic cages (MOCs).

In this sense, the conformational control of molecules is a fundamental aspect of organic chemistry that supports the emergence of functions such as molecular recognition,^6^ catalysis,^7^ materials with optoelectronic properties,^8^ drug design,^9^ and more.

**Fig. 1:**
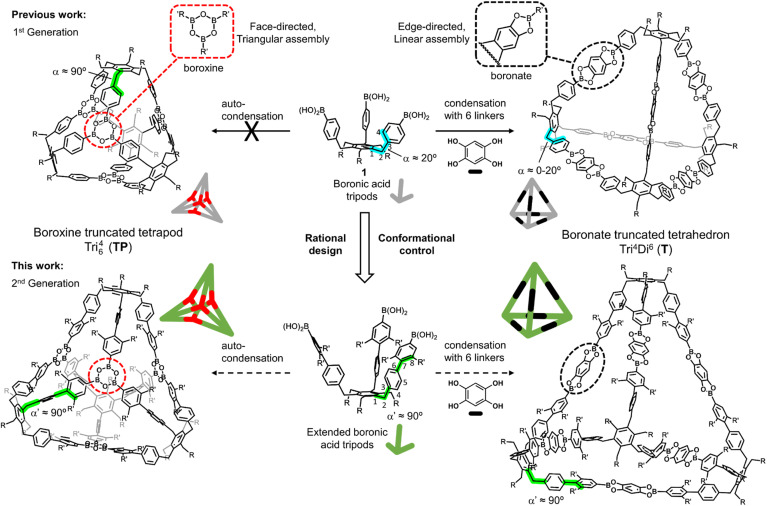
Our sterically-geared, tritopic boronic acid tripod 1 (1^st^ generation) and the extended version (2^nd^ generation) as potential platform for the face-directed assembly of a boroxine truncated tetrapodal (left) and the edge-directed assembly of a boronate truncated tetrahedral cage (right).

Based on our previous studies toward the synthesis of functionalized benzocyclotrimers,^10–12^ we found that the sterically geared, 1,3,5/2,4,6-alternate substitution pattern of the *C*_3_-symmetric boronic acid tripods, precursors 1 (R = Et, OAr), were ideal building blocks for the formation of boronate-truncated tetrahedrons with the topology Tri^4^Di^6^ (ref. 13) by condensation with benzene-1,2,4,5-tetraol (THB) through an edge-directed assembly.^14^ However, the same precursors could not form a boroxine-truncated tetrapod with the topology Tri_6_^4^ (ref. 15) by auto-condensation through a face-directed assembly (Fig. 1).^16^ An in-depth conformational analysis of the tripod precursors indicated that the dihedral angle *α*, defined by atoms 1–2–3–4, was about ±20°. However, because the boroxine rings have a planar trigonal arrangement and are located on the faces of the tetrahedron, the boroxine cage requires the dihedral angle *α* to be close to 90° (Fig. 1). Unfortunately, controlling and modulating this dihedral angle *α* is a non-trivial task. Herein we show that this problem could be circumvented using rational design principles. Conformational control could be induced by extending the tripod's arms. In this sense, we considered that a biphenyl system is the most suitable spacer to accomplish this. If the dihedral angle *α* maintains the value observed (a range of ±20°), the dihedral angle formed by the two aromatic rings of the biphenyl system could be modulated with substituents in the *ortho* positions (5 or 8), modifying the value of *α*′ between atoms 1–2–7–8 to approach 90° (Fig. 1). By extending the tripod's arms by a 2,3-dimethylbenzene unit, we obtained a versatile tripodal building block that can be used to form the previously inaccessible tetrapodal boroxine cage efficiently and further a novel bipyramidal boronate cage. By varying the substitution of the spacer units, we assessed the limits of this steric induction approach. Additionally, the versatility of this extended platform enables the system to undergo a structural transformation (cage metamorphosis) from boroxine to boronate MOCs in solution.

## Results and discussion

### Synthesis of the building blocks

**Scheme 1:**
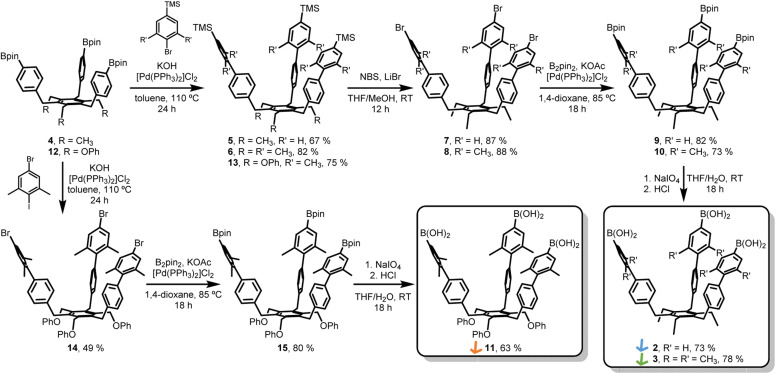
Synthesis of extended triboronic acid precursors 2, 3, and 11.

The synthesis of extended tripods 2 and 3 was planned and carried out (Scheme 1). The non-methylated derivative 2 was synthesized to understand the limits of this conformational control better. Starting from Bpin-tripod 4,^14^ Suzuki coupling with (4-bromophenyl)trimethylsilane or (4-bromo-3,5-dimethylphenyl)trimethylsilane afforded extended tripods 5 and 6 in good yields. The trimethylsilyl groups were converted into corresponding bromide using *N*-bromosuccinimide and LiBr in THF/methanol to furnish the Br-tripods 7 and 8. Miyaura borylation reaction with B_2_pin_2_ gave the extended Bpin-tripods 9 and 10 with good yields. Deprotection under standard oxidative conditions yielded the target triboronic acid tripods 2 and 3. Additionally, the OPh-analog 11 was synthesized to determine the influence of the tripod feet on the assembly process. Bpin-tripod 12 (ref. 14) was coupled with (4-bromo-3,5-dimethylphenyl)trimethylsilane under Suzuki conditions to obtain the TMS-tripod 13. However, the transformation of the trimethylsilyl groups into the bromides suffered from uncontrolled bromination of the OPh-feet. This problem could be circumvented by chemoselective Suzuki coupling of 12 with 5-bromo-1,3-dimethyl-2-iodobenzene to give Br-tripod 14 in a single step with moderate yield. The synthesis of triboronic acid 11 followed the route described above for 2 and 3. This shorter route using the chemoselective Suzuki-coupling was later also applied on the ethyl-substituted tripods 2 and 3 (Scheme 1).

### Cage synthesis and characterization

**Fig. 2:**
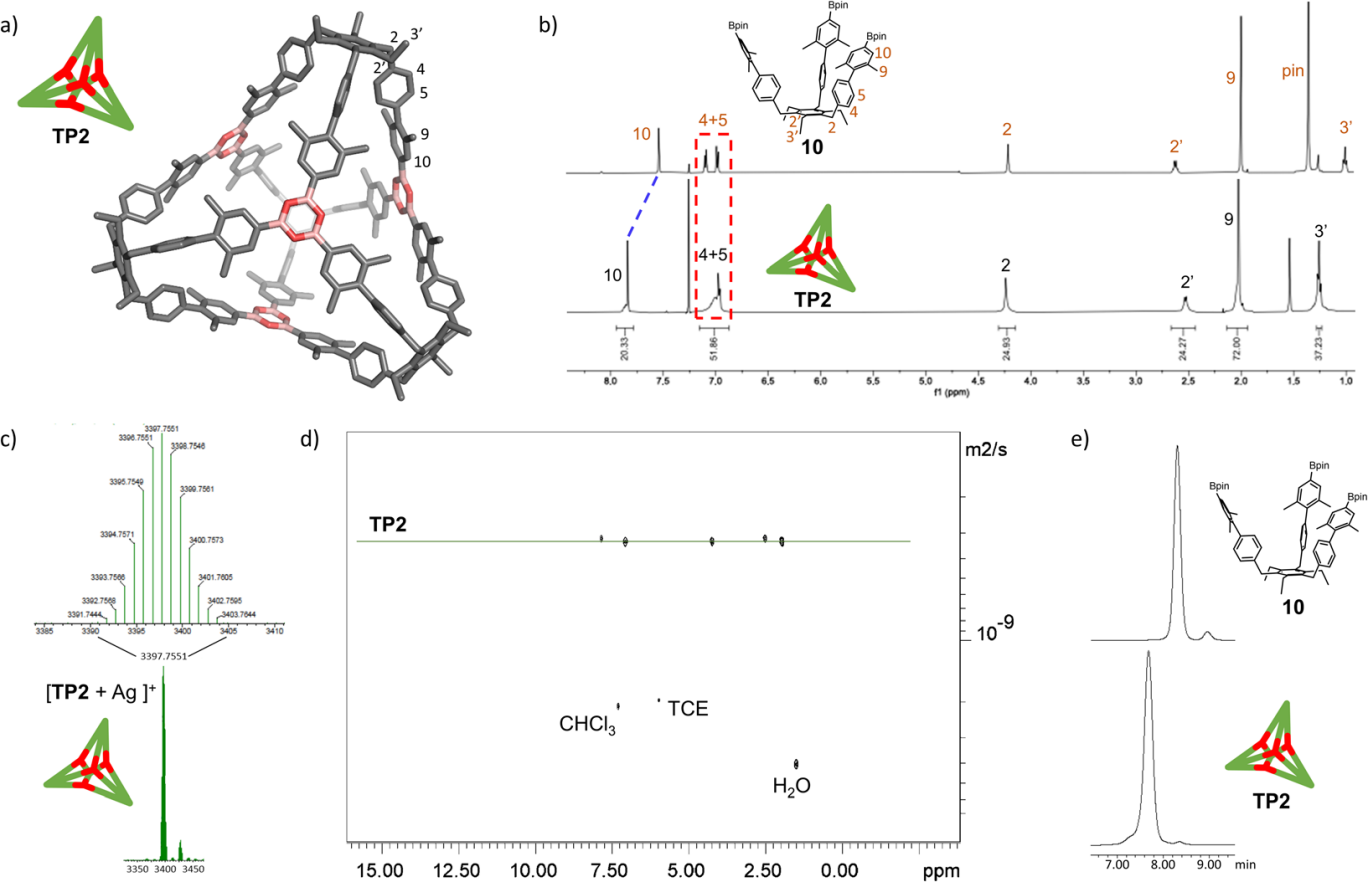
Characterization of boroxine cage TP2. (a) Molecular model of boroxine cage TP2. (b) ^1^H NMR spectra (500 MHz, CDCl_3_, RT) of B-pin precursor 10 and tetrapod cage TP2. (c) High-resolution MALDI-TOF-MS of cage TP2. (d) ^1^H-DOSY-NMR (500 MHz, CDCl_3_) of TP2. (e) GPC traces of B-pin precursor 10 and cage TP2 (92% purity). TCE = 1,1,2,2-tetrachloroethane.

With the extended tripods in hand, the next step was to assemble boroxine tetrapod TP1 (R = CH_3_, R′ = H) using boronic acid tripod 2. For this, we suspended 2 in CDCl_3_ using 2 equiv. of water for each boronic acid moiety and heated the mixture to 110 °C in a sealed pressure tube.^17^ After three days, the reaction mixture was still a suspension, and reaction control *via*^1^H-NMR showed only trace signals of a symmetric species. We prolonged the reaction times to seven days, but no substantial change was observed. Nevertheless, MALDI-TOF-MS analysis of the reaction mixture showed a weak signal corresponding to the target compound, and hence we attributed the signals in the ^1^H-NMR to traces of cage TP1 (see Section 4 in ESI‡). Given the low intensity of the ^1^H-NMR signals, we did not determine the yield and concluded that stronger conformational control is necessary to direct the building blocks to efficiently self-assemble. Therefore, the effect of the methyl groups on the conformational control was tested using 3 as the building block. Upon applying the conditions abovementioned, the insoluble starting material gradually dissolved and turned into a slightly turbid solution after a few hours. The ^1^H-NMR showed the formation of a symmetric product that matched the expected *T*_d_ symmetry. Additionally, a greater magnetic deshielding of the proton signal located in C-10 from the B-pin precursor 10 is in line with the formation of a boroxine functionality. Interestingly, the signals corresponding to the aromatic protons in C-4 and C-5 were broad and undefined, which we preliminarily attributed to slow rotation of the arms on the NMR time scale due to boroxine ring formation (Fig. 2b). MALDI-TOF-MS analysis of the crude mixture demonstrated the formation of the target compound TP2 (R = R′ = CH_3_) (Fig. 2c). A ^1^H-NMR-DOSY spectrum was recorded, showing the presence of one single species with a diffusion coefficient of *D*_TP2_ = 3.25 ± 0.06 × 10^−10^ m^2^ s^−1^, which, based on the Stokes–Einstein equation, corresponds to a volume of *V*_TP2_ = 8597 ± 481 Å^3^ (Fig. 2d).^18^ For comparison, the diffusion coefficient for B-pin precursor 10 was determined to be *D*_10_ = 4.73 ± 0.07 × 10^−10^ m^2^ s^−1^ (*V*_10_ = 2794 ± 132 Å^3^), and the volumetric ratio is, therefore, *V*_TP2_/*V*_10_ = 3.1. Considering that, we compare the B-pinacol precursor with the cage, this agrees well with our expectations. Additionally, the volume was determined based on a geometry-optimized molecular model of the cage (using the OPLS4 force field, see Section 9 in ESI‡). We obtained a volume of *V*_TP2_ = 8310 Å^3^ for the cage, which is in excellent agreement with the DOSY values. Unfortunately, all attempts to grow crystals suitable for X-ray analysis failed.

**Fig. 3:**
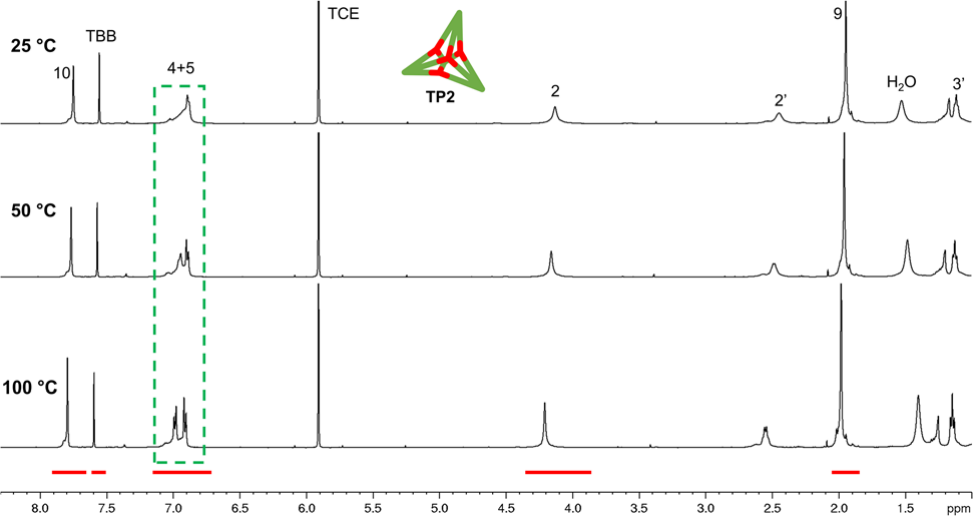
qHNMR spectra (500 MHz) at different temperature of cage TP2 in TCE-*d*_2_. The green dashed rectangle highlights the appearance of a two-spin system of type AB for the aromatic protons located at C-4 and C-5 as the temperature increases. The red bars indicate the fixed integration areas for yield calculation using TBB as the internal standard. TBB = 1,3,5-tribromobenzene, TCE = 1,1,2,2-tetrachloroethane.

The yield of the reaction was calculated by quantitative ^1^H-NMR (qHNMR)^19^ using 1,1,2,2-tetrachloroethane (TCE) as the internal standard. The yield for the self-assembly of TP2 was determined to be 53 ± 3%, which was in agreement with the fact that the reaction mixture never turned completely transparent. To rule out solubility issues, self-assembly of TP2 was carried out using twofold dilution (0.0025 M instead of 0.005 M) but the yield could only be improved to 59 ± 4%. By adding 4 Å molecular sieves to the reaction mixture, we were able to reach 63 ± 5% yield. This could indicate that the assembly's yield is determined by a dynamic equilibrium, which needs to be shifted more actively toward the cage for higher yields. To understand the conformational dynamics of TP2, we carried out high-temperature ^1^H-NMR studies. For this, we suspended building block 3 in TCE-*d*_2_ in a NMR tube. Heating to 110 °C provided a clear solution after two hours. Subsequently, a ^1^H-NMR spectrum at RT indicated the formation of TP2. Then, a ^1^H-NMR spectrum was recorded at 100 °C, and a two-spin system of type AB appears for the aromatic protons located in C-4 and C-5, indicating an increase in the rotation of the arms (green dashed rectangle in Fig. 3). Additionally, integration of the cage signals relative to the rest-proton signal of TCE-*d*_2_ and comparison of the integrals at 25 °C and 100 °C indicated that the yield increased with increasing temperature. This is in congruence with the fact that upon cooling to RT, a white precipitate starts to form, which is readily re-dissolved when the temperature is increased.^20^

To gain more insight into the temperature dependence of the reaction, we added 1,3,5-tribromobenzene (TBB) as an internal standard and performed qHNMR measurements at 25 °C, 50 °C, and 100 °C. The NMR tube was left to equilibrate at each temperature for 30 minutes. Analysis of the integrals shows that the yields increase from *Y*_25 °C_ = 64 ± 4% to *Y*_50 °C_ = 67 ± 5% and *Y*_100 °C_ = 89 ± 7% (Fig. 3).

The thermodynamics of the formation of boroxines from arylboronic acids was studied by Tokunaga and co-workers.^21,22^ It was found that the formation of boroxine comes with an enthalpic cost which is overcompensated by a larger entropy gain (release of water molecules to bulk solvent). Consequently, they observed that an increase in temperature leads to an increase of the boroxine species, which agrees with our observations for the assembly of TP2. In the solvent systems we employ (CDCl_3_ and TCE-*d*_2_), this equilibrium leads to cage assembly in roughly 50% yield determined by qHNMR at RT (after heating at 110 °C), while yield determination at 100 °C, shows a nearly quantitative formation of the cage. Since the yield of TP2 formation depends on the temperature and the presence of water, we assumed that it could be increased by freezing the equilibrium at high temperatures by removing water from the reaction mixture. To test this idea, we formed TP2 in TCE-*d*_2_ (18 h at 110 °C) and added 4 Å molecular sieves at 100 °C. The yield was determined at room temperature using TBB as the internal standard, giving 92 ± 7% of TP2 in 98% purity, according to the GPC trace (see Section 5 in ESI‡). This result demonstrates that the assembly of boroxine cage TP2 is a dynamic equilibrium that depends on the temperature and water content of the solvent, which can be actively modulated to reach a nearly quantitative yield.

The high yield of the assembly is due to the ideal geometry of the building block 3, where *γ*_3_ ≈ 24° (*γ* being the angle between the tripod arm and the perpendicular to the central aromatic ring) and 
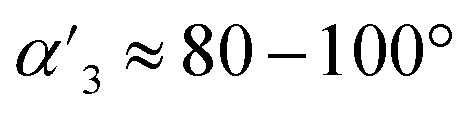
 match well with the geometric requirements *γ*_tetrapod_ = 19.5° and *α*_tetrapod_ = 90° for boroxine tetrapod assembly.

Furthermore, boroxine self-assembly was investigated with OPh tripod 11. After 36 hours at 110 °C in TCE-*d*_2_, the ^1^H-NMR at RT showed a mixture of two main compounds and MALDI-TOF-MS analysis confirmed the formation of TP3 (R = OPh, R′ = CH_3_). ^1^H-NMR analysis of the reaction mixture at 100 °C showed the presence of two species, which were also observed on the size-exclusion chromatography trace and using ^1^H-NMR-DOSY (see Section 4 in ESI‡). This excluded a conformational equilibrium and confirmed that two independent assemblies are present. We conducted several trials including longer reaction times (up to 2 weeks), iterative addition of water, 4 Å MS, different temperatures, and solvents; however, we were unable to shift the reaction toward the exclusive formation of TP3. In previous work we have demonstrated that non-extended tripod 1 (R = OPh) is conformationally “looser” than its counterpart 1 (R = Et).^14^ Due to the structural similarity, we assume that this conformational behaviour can be extrapolated to extended tripod 11. Thus, we hypothesize that conformationally “looser” 11 is less suited for efficiently forming boroxine cages.

### Dynamic behaviour

**Fig. 4:**
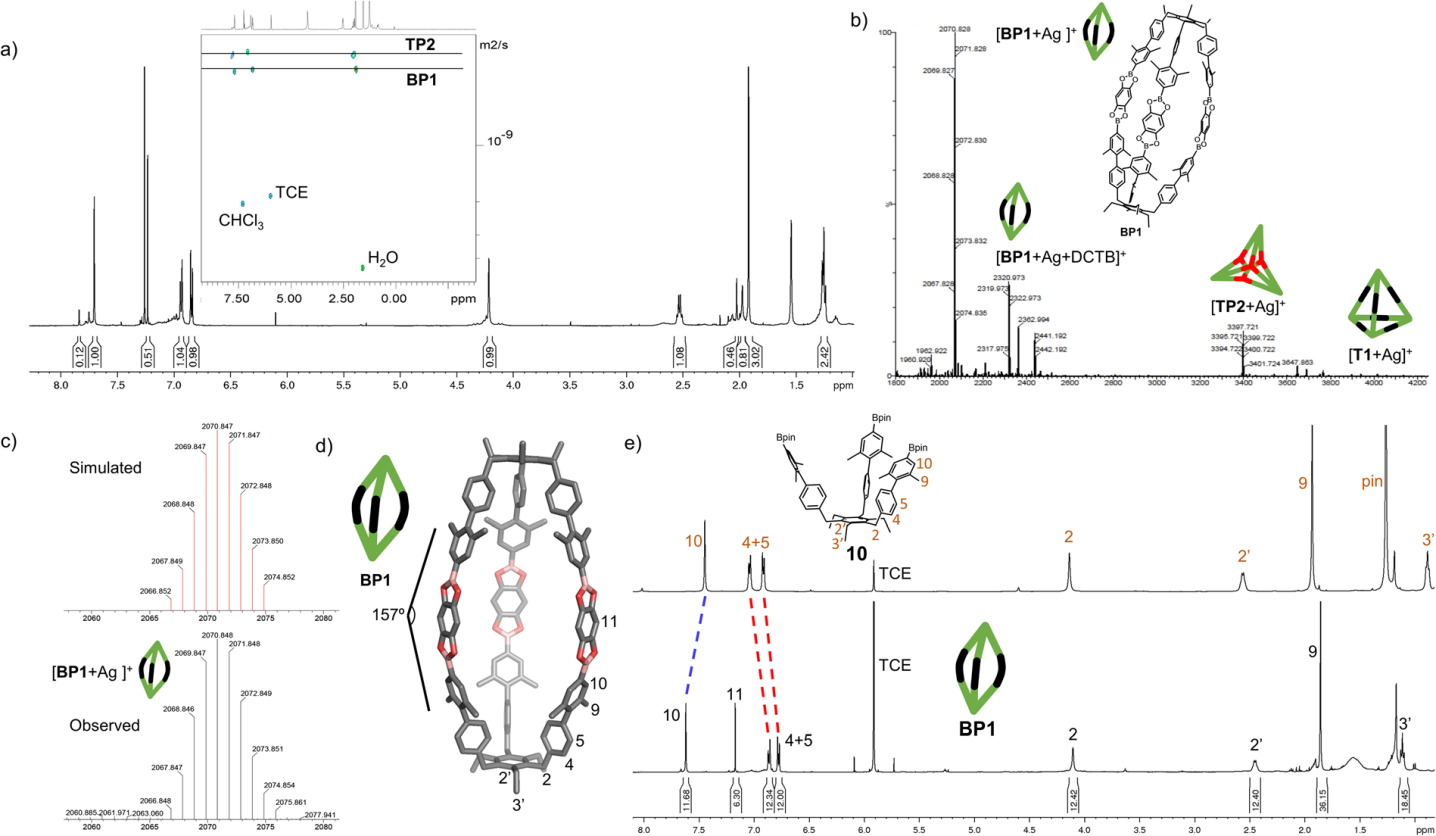
Formation and characterization of boronate cage BP1. (a) ^1^H NMR spectra (500 MHz, CDCl_3_, RT) at 72 hours of reaction. Inset shows ^1^H-DOSY-NMR confirming the presence of two species, the boroxine TP2 and boronate cage BP1. (b) MALDI-TOF-MS full spectrum at 72 hours of reaction. (c) High-resolution MALDI-TOF-MS of cage BP1, showing the observed and simulated isotopic patterns of the [BP1 + Ag]^+^ peak. (d) Molecular model (OPLS4 force field) of the bipyramidal cage BP1. (e) ^1^H NMR spectra (500 MHz, TCE-*d*_2_, RT) of the B-pin precursor 10 and bipyramidal cage BP1 after purification by preparative GPC (99% purity by analytical GPC). DCTB = *trans*-2-[3-(4-*tert*-butylphenyl)-2-methyl-2-propenylidene]malononitrile. TCE = 1,1,2,2-tetrachloroethane.

On the one hand, extended boronic acid tripod 3 was designed to be an ideal building block for forming boroxine tetrapod TP2. On the other hand, the assembly of boronate tetrahedra T is independent of the dihedral angle *α* if a linear linker such as benzene-1,2,4,5-tetraol (THB) is used (edge-directed assembly, Fig. 1). Hence, we set out to understand if extended building block 3 can be used as a bifunctional platform for forming both boroxine and boronate cages. Subjecting 3 and THB in a 4 : 6 ratio to the same conditions for the formation of cage TP2 (CDCl_3_, at 110 °C, sealed tube and 2 equiv. of water for boronic acid) provided a clean solution after 72 hours. The solution was analyzed by ^1^H-NMR and showed the appearance of new signals that fit a new symmetric assembly, along with small signals corresponding to the boroxine cage TP2 and a set of aromatic signals at 7.7–7.8 ppm that we were not able to assign. We attributed the unidentified signals to the presence of reaction intermediate(s), or soluble oligomers. The emergence of a new signal at 7.23 ppm, belonging to the aromatic protons of THB,^23^ indicated the incorporation of this unit into a new boronate structure (Fig. 4a). ^1^H-DOSY-NMR showed two sets of signals corresponding to TP2 (*D*_TP2_ = 3.29 ± 0.13 × 10^−10^ m^2^ s^−1^, *V*_TP2_ = 8356 ± 974 Å^3^), and to the new boronate assembly *D* = 4.00 ± 0.02 × 10^−10^ m^2^ s^−1^ (*V* = 4628 ± 73 Å^3^) (Fig. 4a). The volume obtained from the DOSY measurement of the boronate species is significantly smaller than that obtained for TP2. This result led us to suspect that the novel boronate cage is smaller than the expected tetrahedron with Tri^4^Di^6^ topology. The MALDI-TOF-MS analysis confirmed the formation of the TP2 boroxine. However, the highest intensity peak corresponds to the formation of a [2 + 3] cage with a Tri^2^Di^3^ topology. This bipyramidal cage BP1 (R = R′ = CH_3_) is assembled from two boronic acid tripods and three THB units (Fig. 4b and c). From the molecular modeling study, BP1 shows a rugby ball-like structure with distorted phenyl boronates, with an average strain angle of 157° (Fig. 4d). qHNMR (with TCE as the internal standard) showed that cage BP1 was formed with a yield of 39% and TP2 with a yield of 5%. After 96 hours of reaction, boroxine TP2 was not detected by ^1^H-NMR. The formation of Tri^2^Di^3^ cage BP1 was unforeseen. Based on the directional bonding approach, a tritopic building block with *γ*_3_ ≈ 24° combined with a linear connector (THB, 180°) is expected to give an edge-directed Tri^4^Di^6^ tetrahedron. Emergent behaviour in the form of unpredicted geometrical outcomes has previously been observed in other DCC systems.^24–28^ We attributed this result to the strain energy being overcompensated by the entropic gain of a smaller assembly.

Interestingly, when OPh precursor 11 was used for boronate formation, the reaction was not found to funnel cleanly toward the corresponding cage BP2 (R = OPh, R′ = CH_3_) but rather a complex mixture of differently sized products was obtained, as demonstrated by size-exclusion chromatography (see Section 6 in ESI‡). Nevertheless, MALDI-TOF-MS indicated that BP2 is present in the mixture. This result emphasizes the crucial role of the tripodal feet in the assembly of these structures.

Considering that the formation of boroxine TP2 is significantly faster than boronate BP1, we can assume that when building block 3 is suspended with THB in CDCl_3_ or TCE-*d*_2_ at 110 °C, boroxine TP2 is quickly self-assembled and subsequently transformed into BP1 over days. We denominated this behaviour cage metamorphosis, since the transformation happens from a boroxine cage to a boronate cage. This involves a topological shift from Tri_6_^4^ to Tri^2^Di^3^ and a change of the geometrical shape from a face-assembled tetrapod to an edge-assembled bipyramidoid. Metamorphosis has been achieved in boroxine cages through the addition of pyridine, since they form a 1 : 1 adduct, producing a change in the geometry of the boron-centers from trigonal planar to tetrahedral.^29^ In addition, the interconversion between a tetrahedral Tri^4^Tri^4^ cage and a small Tri^1^Tri^1^ assembly based on imine condensation has been described recently.^30^

However, as far as we are aware, metamorphosis which includes a shift in the topology and a change of the functionality (boroxine to boronate) has not yet been reported. However, to verify the occurrence of the metamorphosis, several matters required resolution: first, it was found that when boroxine TP2 was self-assembled and kinetically “locked” *via* the addition of 4 Å MS, the addition of THB did not lead to the formation of boronate BP1. After 72 hours at 110 °C, only traces of the boronate cage were formed. This result shows that cage metamorphosis cannot be induced solely by the presence of the phenol groups of THB and that partial dissociation of the boroxine rings by water is necessary to induce the change. This is in agreement with observations by Dichtel and co-workers, where the hydrolysis of competing boroxine species was found to be the rate-limiting step in the formation of boronic esters from the same starting materials.^31^

**Fig. 5:**
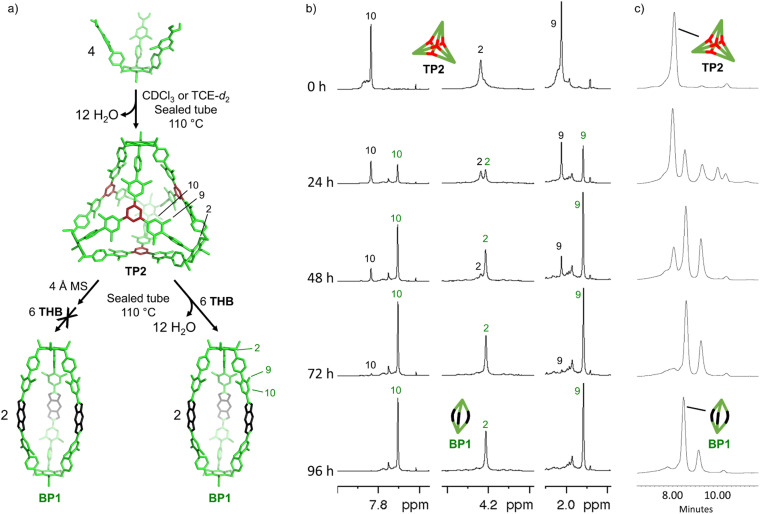
Monitoring the metamorphosis reaction. (a) Schematic representation of the cage metamorphosis. (b) Sections of ^1^H NMR spectra (500 MHz, CDCl_3_, RT) and (c) GPC traces of the metamorphosis at different times. Time 0 hour shows the ^1^H NMR and GPC trace of TP2 assembled under reversible conditions (no molecular sieves, after 72 hours at 110 °C in CDCl_3_). Subsequently to *t* = 0 hour, THB was added.

Second, TP2 was self-assembled under reversible conditions (no molecular sieves, 72 hours at 110 °C in CDCl_3_), confirmed by ^1^H-NMR reaction control (*Y*_TP2_ = 56 ± 7%), and subsequently, THB was added to induce metamorphosis (Fig. 5a). Partial metamorphosis to boronate BP1 was observed after 24 hours (*Y*_TP2_ = 40 ± 4% and *Y*_BP1_ = 35 ± 1%), together with the formation of undefined intermediates. The reaction reached *Y*_BP1_ = 39 ± 3% and *Y*_TP2_ = 10 ± 1% after 48 hours. After 72 hours, boroxine TP2 disappears almost completely, and boronate BP1 reaches 49 ± 3% yield at 96 hours (Fig. 5b). Additionally, the reaction was followed by size-exclusion chromatography. We observed a decrease of boroxine TP2, the appearance of boronate BP1, and minor intermediate species. After 96 hours, these intermediates decline, ultimately resulting in the prevalence of boronate BP1 as the principal product of the metamorphosis (Fig. 5c).

The thermodynamics of the condensation of arylboronic acids with aromatic diols was investigated by Northrop and co-workers.^32^ Similar to boroxine formation, boronate formation is an entropically driven process, and the driving force is the liberation of water molecules to the bulk solvent. At RT, boronate condensation of phenylboronic acid with catechol was found to be exergonic 
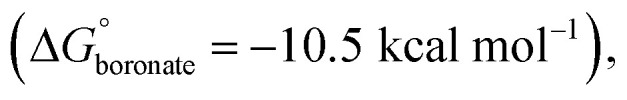
 while auto-condensation of phenylboronic acid to the respective boroxine was described by Tokunaga to be endergonic 
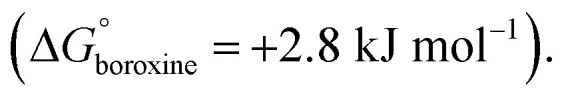
^21^ This energetic difference explains why cage metamorphosis is a spontaneously occurring process. The conversion from TP2 to BP1 is accompanied by the release of 12 additional water molecules (from 12 to 24 molecules) and the formation of two cages of BP1 for each cage of TP2 (Fig. 5a). Additionally, the donation of electron density from oxygen to boron in boronate esters can increase their stability, making them more stable than boroxine rings.

## Conclusions

In conclusion, we designed boronic acid building blocks using rational design principles. Precise transfer of geometric information *via* conformational control was induced by extending the tripod's arms by a 2,3-dimethylbenzene unit enabling the efficient formation of previously inaccessible tetrapodal boroxine cages. Removing the methyl substituents on the benzene unit resulted in the formation of only trace amounts of boroxine cage. This proves the importance of sufficiently controlling the tripods' conformation for effective boroxine assembly. Variable-temperature NMR studies revealed that boroxine cage assembly constitutes a dynamic equilibrium, which readily responds to external stimuli like the amount of water or temperature. Finally, cage metamorphosis from a boroxine tetrapod to a boronate trigonal bipyramid was achieved by adding an aromatic tetraol. The driving force for this process was explained by the entropic gain correlated with the release of additional water molecules and the increased stability of boronates compared to boroxines.

## Data availability

The experimental details and datasets supporting this article are available in the ESI.‡

## Author contributions

MR: conceptualization; data curation; formal analysis; investigation; methodology; visualization; writing – original draft. SDH: investigation; methodology, writing – review and editing. AHD: validation; writing – review and editing. TM: conceptualization; formal analysis; funding acquisition; methodology; project administration; supervision; writing – original draft.

## Conflicts of interest

There are no conflicts to declare.

## Supplementary Material

SC-014-D3SC02920D-s001
