# Supplementary material for: Conformational control enables boroxine-to-boronate cage metamorphosis
Source: Chem Sci. 2023 Oct 5;14(45):12953–60. doi: 10.1039/d3sc02920d (PMC10664459; doi:10.1039/d3sc02920d)
Supplement: SC-014-D3SC02920D-s001 [file SC-014-D3SC02920D-s001.pdf]

Electronic Supplementary Material (ESI) for Chemical Science.
This journal is © The Royal Society of Chemistry 2023

Electronic Supplementary Information

Conformational Control Enables Boroxine-to-Boronate Cage Metamorphosis

Manuel Rondelli,[ab] Samuel Delgado-Hernández,[ac] Antonio H. Daranas[ad] and Tomás Martín*[ad]

[a] Instituto de Productos Naturales y Agrobiología, Consejo Superior de Investigaciones Científicas (IPNA-CSIC), Avda. Astrofísico Francisco Sánchez, 3, 38206 La Laguna, Tenerife, Spain
[b] Doctoral and Postgraduate School, University of La Laguna, Avda. Astrofísico Francisco Sánchez, 38203 La Laguna, Tenerife, Spain
[c] Departamento de Química. Unidad Departamental de Química Analítica, Universidad de La Laguna (ULL), Tenerife 38206, Spain.
[d] Instituto Universitario de Bio-Orgánica "Antonio González", Universidad de La Laguna, Avda. Astrofísico Francisco Sánchez, 2, 38206 La Laguna, Tenerife, Spain

*Email: tmartin@ipna.csic.es

Table of Contents

1. General Methods 3

2. Synthetic procedure 4

3. NMR Spectra 13

4. Synthesis and Characterization of boroxine Cages 26

5. Variable temperature NMR Studies of cage TP2. 41

6. Synthesis and Characterization of boronate Cages 49

7. Cage Metamorphosis 57

8. DOSY-NMR spectra and Volume Approximation 64

9. Molecular Modeling and Volume Approximation 71

10. References 73

1. General Methods

All reactions were performed under N_2 atmosphere in oven-dried glassware with magnetic stirring. For all reactions requiring heat, heated magnetic stirrers were used as a heat source, and Heat-On blocks were used to transfer heat to the round bottom flasks. Unless otherwise indicated, all reagents were purchased from commercial suppliers and used without any further purification. All solvents were purified by standard techniques or by a solvent purification system (PureSolv). Organic solutions were concentrated under reduced pressure on a rotary evaporator or an oil pump. Reactions were monitored through thin layer chromatography (TLC) on silica gel-precoated aluminum plates. Compounds were visualized by use of UV light, or by different stain solutions such as: vanillin with acetic and sulfuric acid in ethanol, potassium permanganate in sodium hydroxide aqueous solution, Ninhydrin solution or 2.5% phosphomolybdic acid in ethanol, and subsequent heating. Anhydrous sodium sulfate was used for drying solutions. Column chromatography were performed on silica gel, 60 Å and 0.2-0.5 mm. NMR spectra were recorded at 298 K on a Bruker NEO 500 spectrometer with a 5 mm broadband BBO cryo-probe equipped with a z-gradient unit with a maximum nominal gradient strength of 65.7 G cm^{-1}. ^{1}H and ^{13}C NMR chemical shifts were referenced to the $CHCl_3$/$CDCl_3$ solvent peak (δ_H 7.26 ppm and δ_C 77.0 ppm), acetone/acetone-*d6* (δ_H 2.05 ppm and δ_C 29.92 ppm) or 1,1,2,2-tetrachloroethane (TCE)/TCE-*d2* (δ_H 5.91 ppm and δ_C 74.20 ppm). The multiplicity of signals is abbreviated as follows: singlet (s), doublet (d), triplet (t), quartet (q), quintet (qui) and multiplet (m). Quantitative ^{1}H-NMR measurements were done using calibrated 90 degrees pulses and relaxation delays of 15 s using 94.7 mM of TCE (in $CDCl_3$) or 103.6 mM of tribromobenzene (TBB) (in TCE-d_2) as the internal standard. DOSY experiments were performed at 298K using the double pulse gradient stimulated echo pulse sequence as implemented in the Bruker library (diffDste) using variable gradient pulse amplitudes. Gradients were varied using a linear scheme that started from 3.3 to 65.7 G/cm in 16 steps. Diffusion delays of 1 ms (little delta), 50 ms (big delta) and relaxation delays of 3 s were used. All DOSY spectra were processed using the diffusion analysis of dynamics center module available in Bruker Topspin 4.1 software. Low- and high-resolution mass spectra were recorded with TOF analyzer mass spectrometers (Waters LCT premier XE) by using electrospray ionization (ESI). Waters SYNAPT XS ion mobility Q-TOF mass spectrometer were used to acquired MALDI spectra. Infrared (FT-IR) spectra are reported in wavenumbers (cm^{-1}). Analytical and preparative HPLC were performed on Waters 600 E, UV detection monitored at 254 nm, using a Phenogel™ column (5µm 500 Å, 300 x 7.8 mm) and Ultrastyragel® column (500 Å, 19 x 300 mm) respectively, with CH_2Cl_2 as solvent at 1 mL/min. Anhydrous $CDCl_3$ was prepared by adding dry 4 Å molecular sieves to a freshly opened bottle of $CDCl_3$ from VWR chemicals and leaving it at least for 48 hours before use.

2. Synthetic procedure

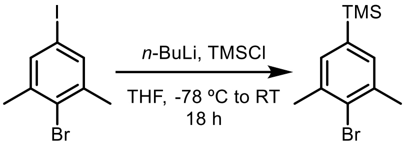

(4-Bromo-3,5-dimethylphenyl)trimethylsilane. Following the literature procedure,[1] 2-bromo-5-iodo-1,3-dimethylbenzene (2.13 g, 6.85 mmol, 1.0 equiv) was dissolved in anhydrous THF (60 mL, 0.11 M), and the solution was cooled to -78 °C. After the addition of *n*-BuLi (1.9 M, 3.61 mL, 6.85 mmol, 1.0 equiv), the reaction was stirred for 30 min, quenched with TMSCl (1.04 mL, 8.22 mmol, 1.2 equiv), and stirred overnight at RT. The reaction mixture was extracted with Et_2O three times, washed with water, brine, and dried over Na_2SO_4. The crude product was purified by column chromatography (Hexane) to give the target compound (1.61 g, 92%) as a white solid.
^{1}H NMR (400 MHz, $CDCl_3$) δ 7.20 (s, 2H), 2.43 (s, 6H), 0.26 (s, 9H).
^{13}C NMR (101 MHz, $CDCl_3$) δ 139.0, 137.7, 133.2, 129.0, 24.0, -1.0.

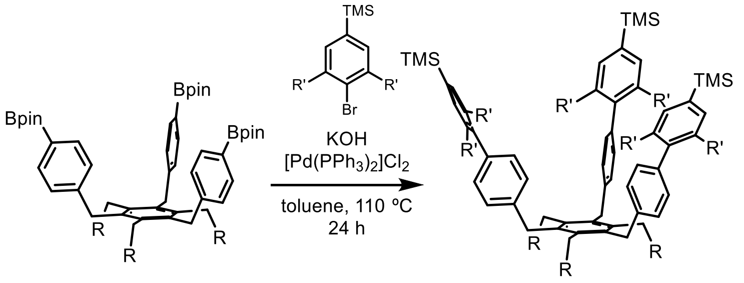

4, R = CH_3
12, R = OPh

5, R = CH_3, R' = H, 67 %
6, R = R' = CH_3, 82 %
13, R = OPh, R' = CH_3, 75 %

General procedure for the Suzuki-coupling of tris(dioxaborolanes). A flame-dried round bottom flask equipped with a reflux condenser was charged with tris(dioxaborolanes) (1.00 equiv), halobenzene (3.3 equiv), anhydrous KOH (9.0 equiv), and $[Pd(PPh_3)_2]Cl_2$ (0.01 equiv).The flask was evacuated and backfilled with N_2 three times, and anhydrous toluene (0.2 M) was added. The resulting mixture was heated to reflux for 24 h. The mixture was allowed to cool to RT, the solvent was removed *in vacuo*, and extracted three times with CH_2Cl_2. The combined organic layers were dried over anhydrous Na_2SO_4, filtered, and concentrated *in vacuo*. The crude product was purified by flash chromatography on silica gel to yield tris(trimethylsilane) extended tripods.

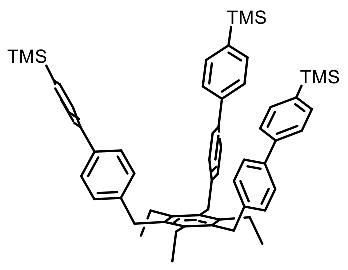

5

(((2,4,6-Triethylbenzene-1,3,5-triyl)tris(methylene))tris([1,1'-biphenyl]-4',4-diyl))-tris-(trimethylsilane) (5). General procedure for the Suzuki-coupling was applied to tris(pinacol-boronate) **4**[2] (500 mg, 619 µmol, 1.0 equiv) using (4-bromophenyl)trimethylsilane (398 µL, 2.04 mmol, 3.3 equiv), KOH (311 mg, 5.55 mmol, 9.0 equiv), and $[Pd(PPh_3)_2]Cl_2$ (2.16 mg, 3.08 µmol, 0.01 equiv) in 7.0 mL anhydrous toluene. Purification by flash chromatography on silica gel (Hexane:EtOAc = 98:2) gave tris(trimethylsilane) **5** (362 mg, 67 % yield) as a white solid.

^{1}H NMR (400 MHz, $CDCl_3$) δ 7.56 (s, 12H), 7.50 (d, *J* = 8.2 Hz, 6H), 7.10 (d, *J* = 8.0 Hz, 6H), 4.20 (s, 6H), 2.53 (q, *J* = 6.9 Hz, 6H), 1.13 (t, *J* = 7.5 Hz, 9H), 0.29 (s, 27H).

^{13}C NMR (101 MHz, $CDCl_3$) δ 141.6 (d, *J* = 2.3 Hz), 140.7, 139.0, 138.8, 134.0, 128.3, 127.2, 126.5, 34.5, 24.0, 15.4, -0.9.

HRMS (ESI) *m/z* calcd. for $C_{60}H_{72}Si_3{}^{109}Ag$ $[M+Ag]^+$ 985.3989, found 985.3980.

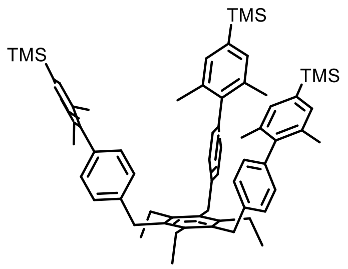

6

(((2,4,6-Triethylbenzene-1,3,5-triyl)tris(methylene))tris(2,6-dimethyl-[1,1'-biphen-yl]-4',4-diyl))tris(trimethylsilane) (6). General procedure for the Suzuki-coupling was applied to tris(pinacol-boronate) **4** (371 mg, 458 µmol, 1.0 equiv) using (4-bromo-3,5-dimethylphenyl)trimethylsilane (389 mg, 1.51 mmol, 3.3 equiv), KOH (231 mg, 4.12 mmol, 9.0 equiv) and $[Pd(PPh_3)_2]Cl_2$ (3.21 mg, 4.58 µmol, 0.01 equiv) in 2.3 mL anhydrous toluene. Purification by flash chromatography on silica gel (Hexane:EtOAc = 95:5) gave tris(trimethylsilane) **6** (361 mg, 82 % yield) as a white solid.

^{1}H NMR (400 MHz, $CDCl_3$) δ 7.22 (s, 6H), 7.09 (d, *J* = 7.8 Hz, 6H), 7.00 (d, *J* = 8.0 Hz, 6H), 4.22 (s, 6H), 2.62 (q, *J* = 7.4 Hz, 6H), 2.00 (s, 18H), 1.01 (t, *J* = 7.4 Hz, 9H), 0.27 (s, 27H).

^{13}C NMR (101 MHz, $CDCl_3$) δ 142.6, 141.4, 140.1, 138.8, 138.4, 135.5, 134.5, 132.4, 129.0, 128.1, 34.8, 24.0, 21.0, 15.2, -0.9.

HRMS (ESI) *m/z* calcd. for $C_{66}H_{84}Si_3{}^{109}Ag$ $[M+Ag]^+$ 1069.4928, found 1069.4939.

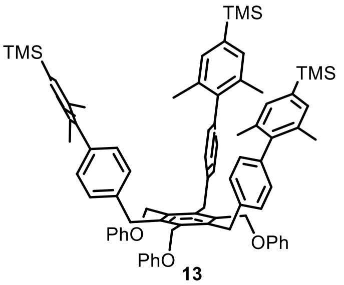

13

(((2,4,6-Tris(phenoxymethyl)benzene-1,3,5-triyl)tris(methylene))tris(2,6-dimethyl-[1,1'-biphenyl]-4',4-diyl))tris(trimethylsilane) (13). General procedure for the Suzuki-coupling was applied to tris(pinacol-boronate) **12**[2] (617 mg, 591 µmol, 1.0 equiv) using (4-bromo-2,3-dimethylphenyl)trimethylsilane (501 mg, 1.95 mmol, 3.3 equiv), KOH (298 mg, 5.32 mmol, 9.0 equiv) and $[Pd(PPh_3)_2]Cl_2$ (4.15 mg, 5.91 µmol, 0.01 equiv) in 15 mL anhydrous toluene. Purification by flash chromatography on silica gel (Hexane:EtOAc = 9:1) gave tris(trimethylsilane) **13** (528 mg, 75 % yield) as a white solid.

^{1}H NMR (400 MHz, $CDCl_3$) δ 7.24 – 7.19 (m, 6H), 7.14 (d, *J* = 7.9 Hz, 6H), 7.08 – 7.01 (m, 6H), 6.93 (t, *J* = 7.4 Hz, 3H), 6.79 (d, *J* = 8.1 Hz, 6H), 5.08 (s, 6H), 4.44 (s, 6H), 2.02 (t, *J* = 2.2 Hz, 18H), 0.31 (t, *J* = 2.1 Hz, 27H).

^{13}C NMR (101 MHz, $CDCl_3$) δ 158.9, 142.4, 142.2, 139.0, 138.8, 135.5, 134.7, 132.5, 129.5, 129.1, 128.3, 121.1, 114.7, 65.0, 35.3, 21.0, -0.9.

HRMS (ESI) *m/z* calcd. for $C_{81}H_{89}Si_3O_3{}^{109}Ag$ $[M+Ag]^+$ 1301.5243, found 1301.5261.

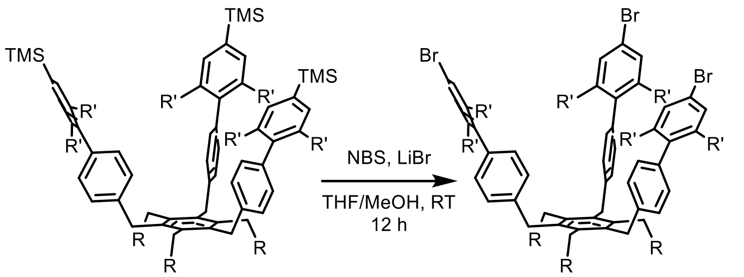

5, R = CH_3, R' = H
6, R = R' = CH_3
7, R = CH_3, R' = H, 87 %
8, R = R' = CH_3, 88 %

General procedure for the bromination of tris(trimethylsilanes). To a solution of tris(trimethylsilane) (1.0 equiv) in MeOH (0.05 M) was added LiBr (9.0 equiv). Next, a solution of NBS (9.0 equiv) in THF (0.05 M) was added, and the resulting mixture was stirred overnight. Upon completion, the reaction was stopped with water, and extracted with CH_2Cl_2 (three times). The combined organic layers were dried over Na_2SO_4, filtrated, and concentrated *in vacuo.* Purification by flash column chromatography on silica gel afforded the product as a white solid.

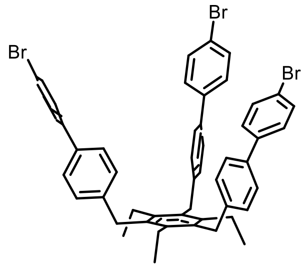

7

4',4''',4'''''-((2,4,6-Triethylbenzene-1,3,5-triyl)tris(methylene))tris(4-bromo-1,1'-biphenyl) (7). General procedure for the bromination was applied to tris(trimethylsilane) **5** (210 mg, 239 µmol, 1.0 equiv) using LiBr (187 mg, 2.15 mmol, 9.0 equiv), NBS (383 mg, 2.15 mmol, 9.0 equiv). Purification by flash chromatography on silica gel (Hexane:EtOAc = 98:2) gave tris(bromide) **7** (186 mg, 87 % yield) as a white solid.

^{1}H NMR (400 MHz, $CDCl_3$) δ 7.57 – 7.50 (m, 6H), 7.49 – 7.40 (m, 12H), 7.13 – 7.07 (m, 6H), 4.20 (s, 6H), 2.51 (q, *J* = 7.5 Hz, 6H), 1.14 (t, *J* = 7.5 Hz, 9H).

^{13}C NMR (101 MHz, $CDCl_3$) δ 141.6, 141.1, 140.0, 137.6, 133.9, 132.0, 128.7, 128.4, 127.0, 121.4, 34.4, 24.0, 15.4.

HRMS (ESI) *m/z* calcd. for $C_{51}H_{45}{}^{79}Br2^{81}Br^{109}Ag$ $[M+Ag]^+$ 1007.0098, found 1005.0113.

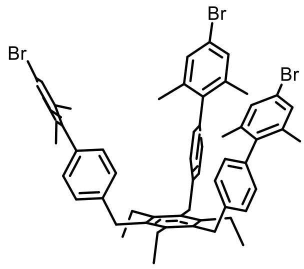

8

4',4''',4'''''-((2,4,6-Triethylbenzene-1,3,5-triyl)tris(methylene))tris(4-bromo-2,6-dimethyl-1,1'-biphenyl) (8). General procedure for the bromination was applied to tris(trimethylsilane) **6** (335 mg, 348 µmol, 1.0 equiv) using LiBr (272 mg, 3.14 mmol, 9.0 equiv), NBS (558 mg, 3.14 mmol, 9.0 equiv). Purification by flash chromatography on silica gel (Hexane:EtOAc = 98:2) gave tris(bromide) **8** (300 mg, 88 % yield) as a white solid.

^{1}H NMR (400 MHz, $CDCl_3$) δ 7.23 (s, 6H), 7.15 – 7.06 (m, 6H), 6.97 (d, *J* = 8.1 Hz, 6H), 4.23 (s, 1H), 2.61 (q, *J* = 7.3 Hz, 6H), 1.96 (d, *J* = 2.2 Hz, 18H), 1.06 – 0.98 (m, 9H).

^{13}C NMR (101 MHz, $CDCl_3$) δ 141.5, 140.9, 140.5, 138.5, 137.4, 134.4, 130.1, 128.9, 128.3, 120.6, 34.8, 24.0, 20.8, 20.7, 15.2.

HRMS (ESI) *m/z* calcd. for $C_{57}H_{75}{}^{79}Br_2{}^{81}Br^{109}Ag$ $[M+Ag]^+$ 1089.1037, found 1089.1025.

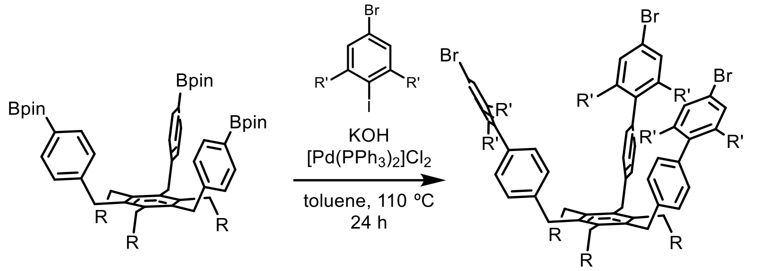

General procedure for the chemoselective Suzuki coupling of tris(dioxaborolanes). A flame-dried round bottom flask equipped with a reflux condenser was charged with tris(dioxaborolanes) (1.0 equiv), 1-bromo-4-iodobenzene derivative (3.3 equiv), anhydrous KOH (9.0 equiv) and $[Pd(PPh_3)_2]Cl_2$ (0.01 equiv).The flask was evacuated and backfilled with N_2 three times, and anhydrous toluene (0.2 M) was added. The resulting mixture was heated to reflux for 24 h. The mixture was allowed to cool to RT, the solvent removed *in vacuo*, and extracted three times with CH_2Cl_2. The combined organic layers were dried over anhydrous Na_2SO_4, filtered, and concentrated *in vacuo*. The crude product was purified by flash chromatography on silica gel to yield the corresponding tris(bromide) as a white solid.

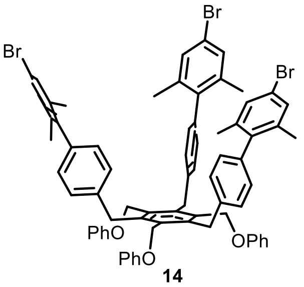

14

4',4''',4'''''-((2,4,6-Tris(phenoxymethyl)benzene-1,3,5-triyl)tris(methylene))tris(4-bromo-2,6-dimethyl-1,1'-biphenyl) (14). General procedure for the chemoselective Suzuki coupling was applied to tris(dioxaborolanes) **12** (351 mg, 336 μmol, 1.0 equiv) using 5-bromo-2-iodo-1,3-dimethylbenzene (345 mg, 1.11 mmol, 3.3 equiv), KOH (170 mg, 3.02 mmol, 9.0 equiv), and $[Pd(PPh_3)_2]Cl_2$ (2.36 mg, 3.36 μmol, 0.01 equiv) in 5.0 mL anhydrous toluene. Purification by flash chromatography on silica gel (Hexane:EtOAc = 95:5) gave tris(bromide) **14** (200 mg, 49 % yield) as a white solid.

^{1}H NMR (400 MHz, $CDCl_3$) δ 7.24 (s, 6H), 7.22 – 7.17 (m, 6H), 7.12 (d, *J* = 7.7 Hz, 6H), 6.99 – 6.95 (m, 6H), 6.92 (t, *J* = 7.4 Hz, 3H), 6.76 (d, *J* = 8.1 Hz, 6H), 5.04 (s, 6H), 4.41 (s, 6H), 1.94 (d, *J* = 2.4 Hz, 18H).

^{13}C NMR (101 MHz, $CDCl_3$) δ 158.8, 142.1, 140.7, 139.2, 138.5, 137.8, 134.8, 130.1, 129.5, 129.1, 128.4, 121.2, 120.7, 114.6, 65.0, 35.3, 20.8.

HRMS (ESI) *m/z* calcd. for $C_{72}H_{63}{}^{79}Br2^{81}Br^{109}Ag$ $[M+Ag]^+$ 1323.1354, found 1323.1394.

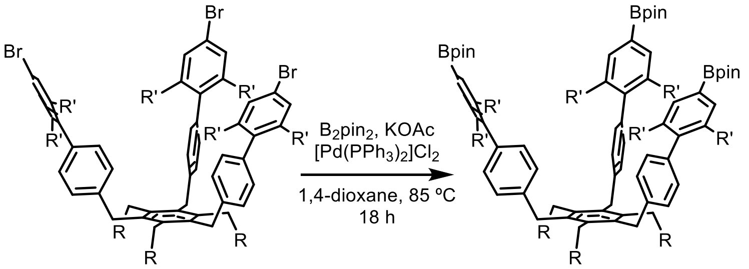

7, R = CH_3, R' = H
8, R = R' = CH_3
14, R = OPh, R' = CH_3

9, R = CH_3, R' = H, 82 %
10, R = R' = CH_3, 73 %
15, R = OPh, R' = CH_3, 80 %

General procedure for the Suzuki-Miyaura borylation of extended tris(bromides). Following the literature procedure,[3] a flame-dried round bottom flask equipped with a reflux condenser was charged with tris(bromides) (1.00 equiv), B_2pin_2 (9.0 equiv), anhydrous KOAc (4.5 equiv), and $[Pd(PPh_3)_2]Cl_2$ (0.1 equiv). The flask was evacuated and backfilled with N_2 three times, and anhydrous 1,4-dioxane (0.2 M) was added. The resulting mixture was heated to 85 °C for 24 h. The mixture was allowed to cool to RT, diluted with H_2O, and extracted three times with EtOAc. The combined organic layers were dried over anhydrous Na_2SO_4, filtered, and concentrated *in vacuo*. The crude product was purified by flash chromatography on silica gel to yield the corresponding extended tris(dioxaborolanes) as a white solid.

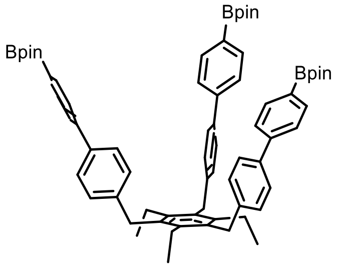

9

2,2',2''-(((2,4,6-Triethylbenzene-1,3,5-triyl)tris(methylene))tris([1,1'-biphenyl]-4',4-diyl))tris(4,4,5,5-tetramethyl-1,3,2-dioxaborolane) (9). General procedure for the Suzuki-Miyaura borylation was applied to tris(bromide) **7** (40.0 mg, 44.6 µmol, 1.0 equiv) using B_2pin_2 (102 mg, 401 µmol, 9.0 equiv), KOAc (19.7 mg, 201 µmol, 4.5 equiv), and $[Pd(PPh_3)_2]Cl_2$ (3.13 mg, 4.46 µmol, 0.1 equiv) in 1.0 mL anhydrous 1,4-dioxane. Purification by flash chromatography on silica gel (Hexane:EtOAc = 85:15) gave tris(dioxaborolane) **9** (38 mg, 82 % yield) as a white solid.

^{1}H NMR (400 MHz, $CDCl_3$) δ 7.91 – 7.81 (m, 6H), 7.60 – 7.57 (m, 6H), 7.56 – 7.51 (m, 6H), 7.10 (d, *J* = 8.0 Hz, 6H), 4.20 (s, 6H), 2.51 (q, *J* = 7.4 Hz, 6H), 1.36 (s, 36H), 1.14 (t, *J* = 7.4 Hz, 9H).

^{13}C NMR (101 MHz, $CDCl_3$) δ 143.8, 141.6, 141.0, 138.6, 135.4, 134.0, 128.3, 127.3, 126.4, 83.9, 34.5, 25.0, 24.0, 15.4. *the ^{13}C-signal corresponding to Ar-*C*-Bpin was not detected.

HRMS (ESI) *m/z* calcd. for $C_{69}H_{81}{}^{11}B_3O_6{}^{109}Ag$ $[M+Ag]^+$ 1146.5436, found 1146.5421.

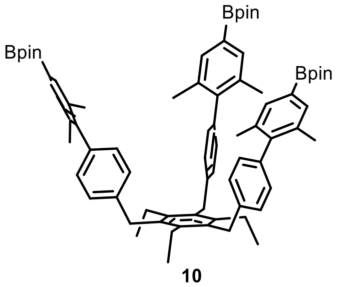

10

2,2',2''-(((2,4,6-Triethylbenzene-1,3,5-triyl)tris(methylene))tris(2,6-dimethyl-[1,1'-biphenyl]-4',4-diyl))tris(4,4,5,5-tetramethyl-1,3,2-dioxaborolane) (10). General procedure for the Suzuki-Miyaura borylation was applied to tris(bromide) **8** (315 mg, 321 μmol, 1.0 equiv) using B_2pin_2 (733 mg, 2.89 mmol, 9.0 equiv), KOAc (142 mg, 1.44 μmol, 4.5 equiv), and $[Pd(PPh_3)_2]Cl_2$ (22.5 mg, 32.1 μmol, 0.1 equiv) in 1.6 mL anhydrous 1,4-dioxane. Purification by flash chromatography on silica gel (Hexane:EtOAc = 9:1) gave tris(dioxaborolane) **10** (265 mg, 73 % yield) as a white solid.
^{1}H NMR (400 MHz, $CDCl_3$) δ 7.56 (s, 6H), 7.12 (d, *J* = 7.9 Hz, 6H), 7.00 (d, *J* = 8.1 Hz, 6H), 4.23 (s, 6H), 2.64 (q, *J* = 7.4 Hz, 6H), 2.01 (s, 18H), 1.37 (s, 36H), 1.02 (t, *J* = 7.4 Hz, 9H).
^{13}C NMR (101 MHz, $CDCl_3$) δ 144.8, 141.1, 139.9, 138.0, 135.4, 134.1, 133.4, 128.4, 127.8, 127.0, 83.5, 83.3, 24.6, 20.4, 14.9.
HRMS (ESI) *m/z* calcd. for $C_{75}H_{93}{}^{11}B_3O_6Na$ $[M+Na]^+$ 1145.7149, found 1145.7234.

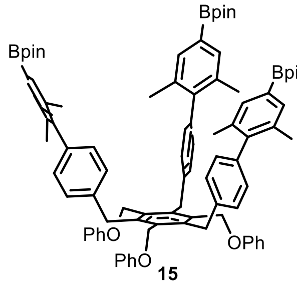

15

2,2',2''-(((2,4,6-Tris(phenoxymethyl)benzene-1,3,5-triyl)tris(methylene))tris(2,6-di-methyl-[1,1'-biphenyl]-4',4-diyl))tris(4,4,5,5-tetramethyl-1,3,2-dioxaborolane) (15). General procedure for the Suzuki-Miyaura borylation was applied to tris(bromide) **14** (231 mg, 190 μmol, 1.0 equiv) using B_2pin_2 (435 mg, 1.71 mmol, 9.0 equiv), KOAc (84.0 mg, 865 μmol, 4.5 equiv), and $[Pd(PPh_3)_2]Cl_2$ (13.4 mg, 19.0 μmol, 0.1 equiv) in 10.0 mL anhydrous 1,4-dioxane. Purification by flash chromatography on silica gel (Hexane:EtOAc = 8:2) gave tris(dioxaborolane) **15** (207 mg, 80 % yield) as a white solid.
^{1}H NMR (400 MHz, $CDCl_3$) δ 7.56 (s, 1H), 7.21 (t, 1H), 7.12 (d, *J* = 7.8 Hz, 1H), 7.00 (d, *J* = 8.0 Hz, 1H), 6.92 (t, *J* = 7.3 Hz, 1H), 6.76 (d, *J* = 8.2 Hz, 1H), 5.04 (s, 1H), 4.41 (s, 1H), 1.99 (s, 2H), 1.37 (s, 6H).
^{13}C NMR (101 MHz, $CDCl_3$) δ 158.8, 144.9, 142.2, 138.9, 138.7, 135.7, 134.7, 133.8, 129.5, 128.9, 128.3, 121.1, 114.6, 83.9, 65.0, 35.3, 25.0, 20.7.
HRMS (ESI) *m/z* calcd. for $C_{90}H_{99}{}^{11}B_3O_9Na$ $[M+Na]^+$ 1379.7466, found 1379.7504.

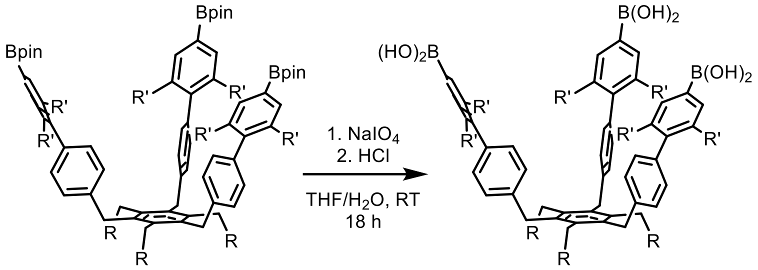

9, R = CH_3, R' = H
10, R = R' = CH_3
15, R = OPh, R' = CH_3

2, R = CH_3, R' = H, 73 %
3, R = R' = CH_3, 78 %
11, R = OPh, R' = CH_3, 63 %

General procedure for the deprotection of tris(pinacolboronates). According to the literature procedure,[4] tris(pinacolboronate) was dissolved in a 4:1 mixture of THF/H_2O (0.09 M:0.35 M) and $NaIO_4$ (12.0 equiv) was added. The resulting mixture was stirred at RT for 30 min following addition of HCl (1.0 M, 3.0 equiv), and additional stirring for 18 hours. Upon completion of the reaction, water was added to dissolve the precipitated salts, and the aqueous phase was extracted three times with EtOAc. The combined organic layers were dried over anhydrous Na_2SO_4, filtered, and concentrated *in vacuo*. The crude product was purified by flash chromatography on silica gel to yield the tris(boronic acids) as off-white solids. The NMR-spectra were recorded in acetone-d_6 with one drop of D_2O to facilitate solvation and hydrolyze boroxines.

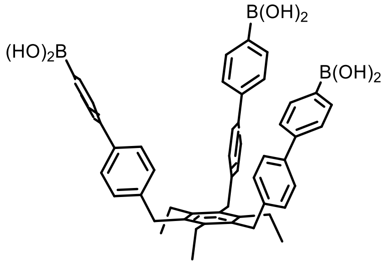

2

(((2,4,6-Triethylbenzene-1,3,5-triyl)tris(methylene))tris([1,1'-biphenyl]-4',4-diyl))-triboronic acid (2). General procedure for the deprotection of tris(pinacolboronates) was applied to tris(dioxaborolane) **9** (103 mg, 99.2 µmol, 1.0 equiv) using $NaIO_4$ (255 mg, 1.19 mmol, 12 equiv), HCl (297 µL, 1.0 M, 297 µmol, 3.0 equiv) in 1.4 mL THF/H_2O. Purification by flash chromatography on silica gel (CH_2Cl_2:MeOH = 95:5) gave triboronic acid **2** (73 mg, 73 % yield) as an off-white solid.

^{1}H NMR (400 MHz, Acetone-d_6) δ 7.91 (d, *J* = 8.0 Hz, 6H), 7.73 – 7.54 (m, 12H), 7.36 – 7.07 (m, 6H), 4.26 (s, 6H), 2.56 (t, *J* = 7.5 Hz, 6H), 1.10 (t, *J* = 7.3 Hz, 9H).

^{13}C NMR (101 MHz, Acetone-d_6) δ 143.1, 142.1, 141.8, 139.3, 135.6, 134.8, 129.1, 127.7, 126.5, 34.8, 24.4, 15.6. *the ^{13}C-signal corresponding to Ar-*C*-Bpin was not detected.

HRMS (ESI) *m/z* calcd. for $C_{51}H_{51}{}^{11}B_3O_6{}^{109}Ag$ $[M+Ag]^+$ 901.3012, found 901.3035.

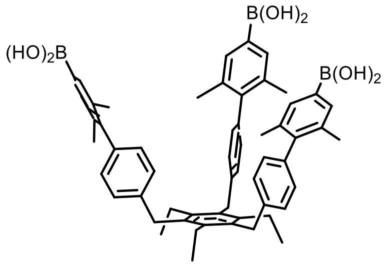

3

(((2,4,6-Triethylbenzene-1,3,5-triyl)tris(methylene))tris(2,6-dimethyl-[1,1'-biphenyl-]-4',4-diyl))triboronic acid (3). General procedure for the deprotection of tris(pinacolboronates) was applied to tris(dioxaborolane) **10** (374 mg, 333 µmol, 1.0 equiv) using $NaIO_4$ (854 mg, 4.00 mmol, 12 equiv), HCl (999 µL, 1.0 M, 999 µmol, 3.0 equiv) in 4.0 mL THF/H_2O. Purification by flash chromatography on silica gel (CH_2Cl_2:MeOH = 95:5) gave triboronic acid **3** (228 mg, 78 % yield) as an off-white solid.
^{1}H NMR (400 MHz, Acetone-d_6) δ 7.55 (s, 6H), 7.17 (d, J = 7.8 Hz, 6H), 7.01 (d, J = 8.0 Hz, 6H), 4.28 (s, 6H), 2.66 (q, J = 7.3 Hz, 18H), 1.94 (s, 3H), 1.02 (t, J = 7.4 Hz, 9H).
^{13}C NMR (101 MHz, Acetone-d_6) δ 144.5, 142.0, 141.0, 139.4, 135.3, 135.3, 134.1, 129.5, 128.9, 35.1, 24.4, 20.9, 15.5. *the ^{13}C-signal corresponding to Ar-*C*-Bpin was not detected.
HRMS (ESI) *m/z* calcd. for $C_{57}H_{63}{}^{13}B_3O_6{}^{107}Ag$ [M+Ag]$^+$ 983.3955, found 983.3967.

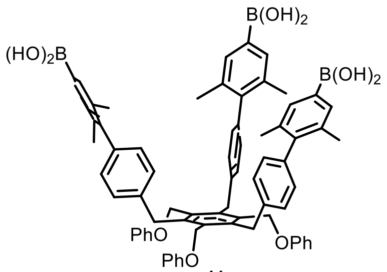

11

(((2,4,6-Tris(phenoxymethyl)benzene-1,3,5-triyl)tris(methylene))tris(2,6-dimethyl-[1,1'-biphenyl]-4',4-diyl))triboronic acid (11). General procedure for the deprotection of tris(pinacolboronates) was applied to tris(dioxaborolane) **15** (157 mg, 116 µmol, 1.0 equiv) using $NaIO_4$ (297 mg, 1.39 mmol, 12 equiv), HCl (347 µL, 1.0 M, 347 µmol, 3.0 equiv) in 1.5 mL THF/H_2O. Purification by flash chromatography on silica gel (CH_2Cl_2:MeOH = 95:5) gave triboronic acid **11** (34 mg, 63 % yield) as an off-white solid.
^{1}H NMR (400 MHz, Acetone-d_6) δ 7.56 (s, 6H), 7.38 – 7.10 (m, 12H), 6.99 (d, J = 8.0 Hz, 6H), 6.88 (t, J = 7.3 Hz, 3H), 6.85 – 6.79 (m, 6H), 5.18 (s, 6H), 4.49 (s, 6H), 1.92 (s, 18H).
^{13}C NMR (101 MHz, Acetone-d_6) δ 159.7, 144.3, 142.9, 139.8, 139.6, 135.6, 135.3, 134.1, 130.2, 129.5, 129.1, 121.7, 115.3, 65.7, 35.5, 20.9.
HRMS (MALDI) *m/z* calcd. for $C_{72}H_{69}{}^{11}B_3O_9{}^{107}Ag$ [M+Ag]$^+$ 1217.4272, found 1217.4282.

3. NMR Spectra

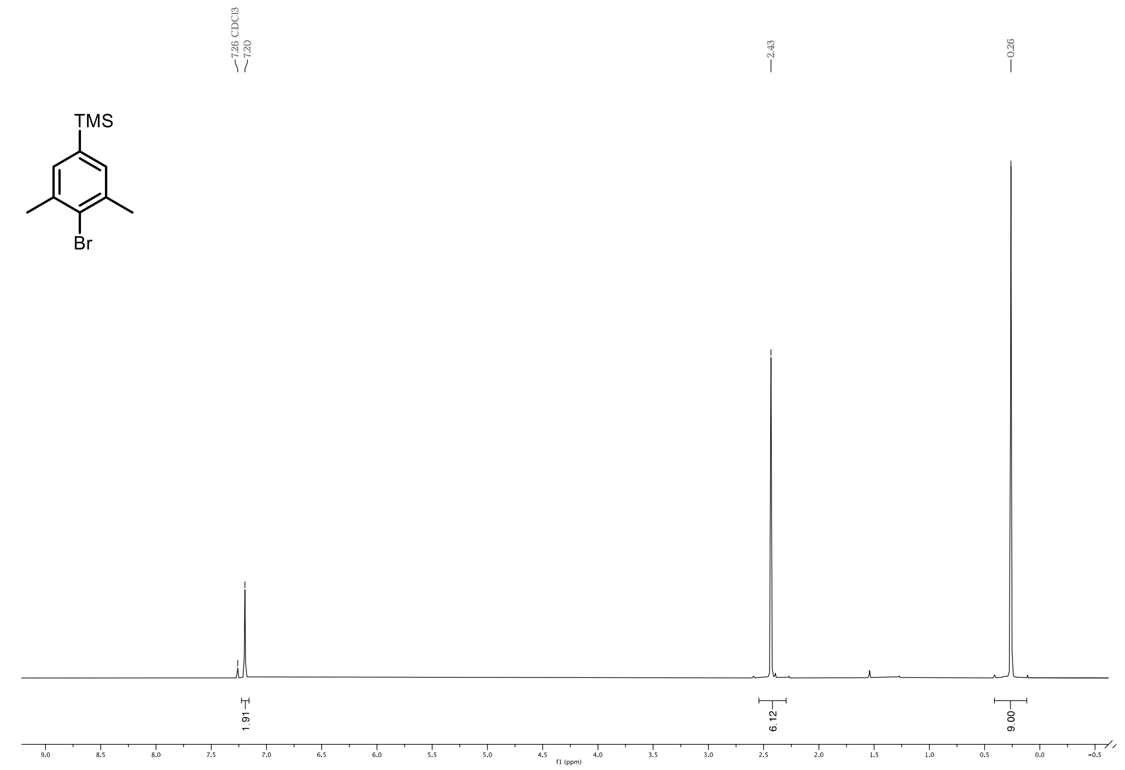

Fig. S1: ^{1}H-NMR (400 MHz, $CDCl_3$) spectrum of (4-bromo-3,5-dimethylphenyl)trimethylsilane.

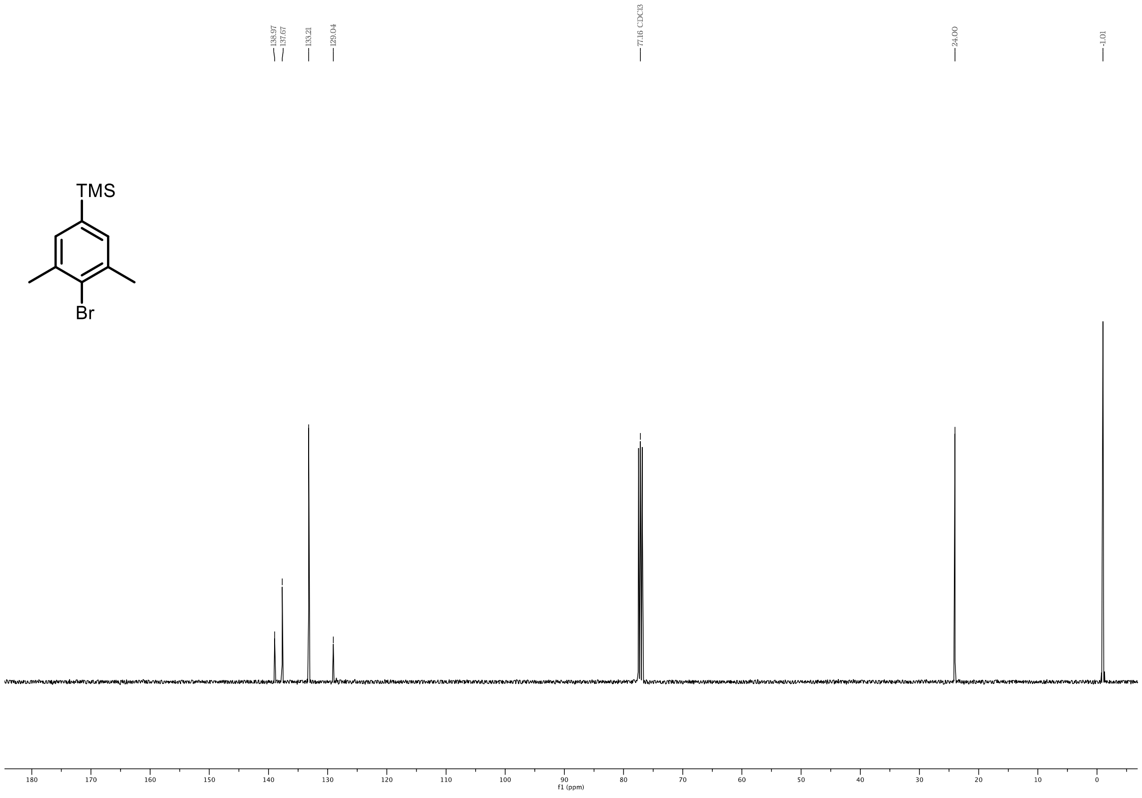

Fig. S2: ^{13}C-NMR (101 MHz, $CDCl_3$) spectrum of (4-bromo-3,5-dimethylphenyl)trimethylsilane.

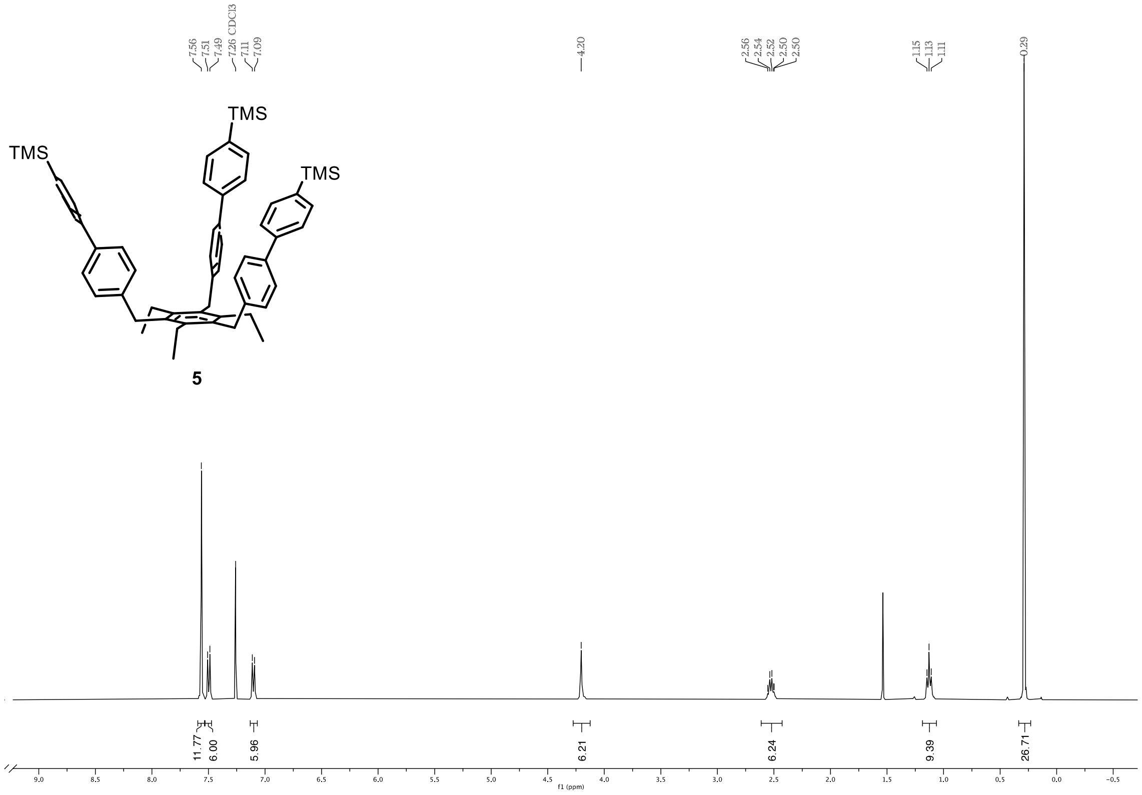

Fig. S3: ^{1}H-NMR (400 MHz, $CDCl_3$) spectrum of (((2,4,6-triethylbenzene-1,3,5-triyl)tris(methylene))tris([1,1'-biphenyl]-4',4-diyl))-tris(trimethylsilane) (**5**).

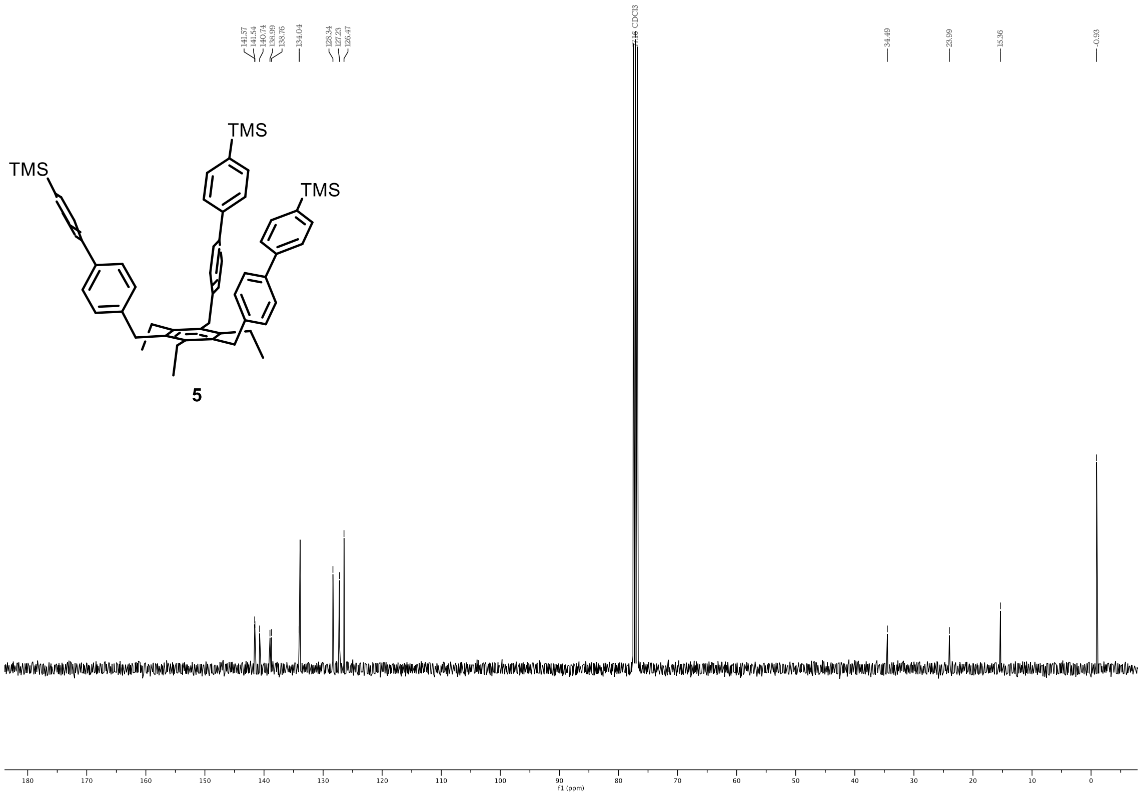

Fig. S4: ^{13}C-NMR (101 MHz, $CDCl_3$) spectrum of (((2,4,6-triethylbenzene-1,3,5-triyl)tris(methylene))tris([1,1'-biphenyl]-4',4-diyl))-tris(trimethylsilane) (**5**).

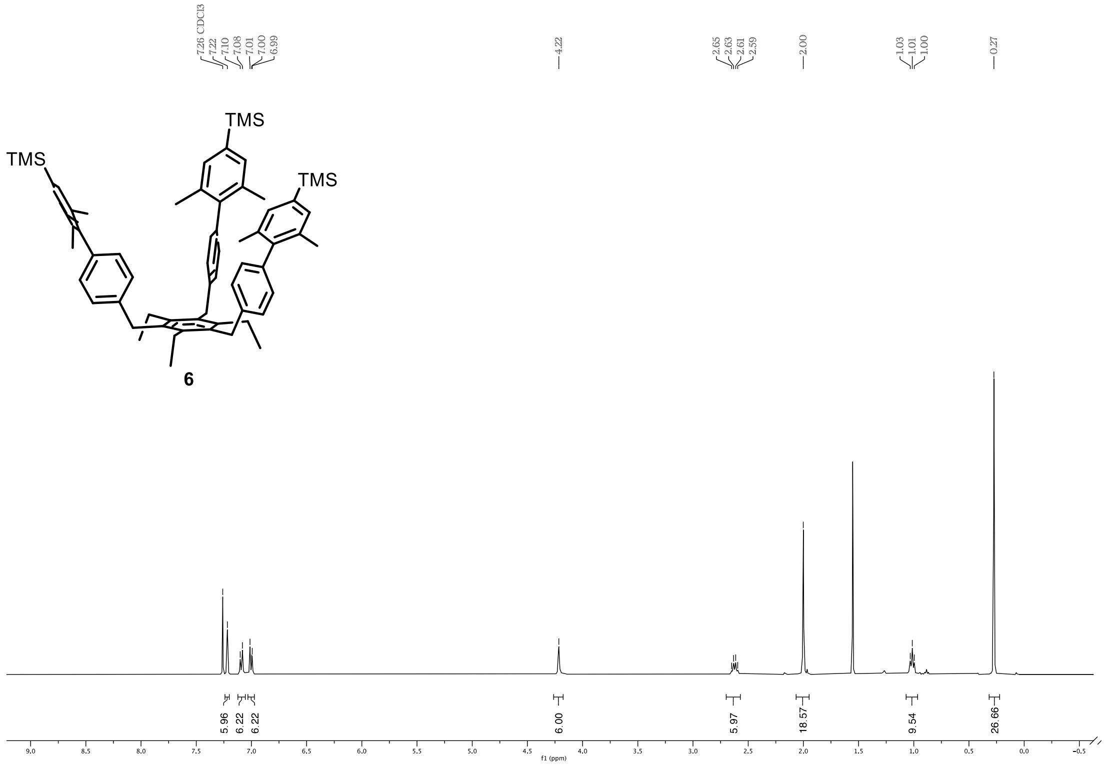

Fig. S5: ^{1}H-NMR (400 MHz, $CDCl_3$) spectrum of (((2,4,6-triethylbenzene-1,3,5-triyl)tris(methylene))tris(2,6-dimethyl-[1,1'-biphenyl]-4',4-diyl))tris(trimethylsilane)(**6**).

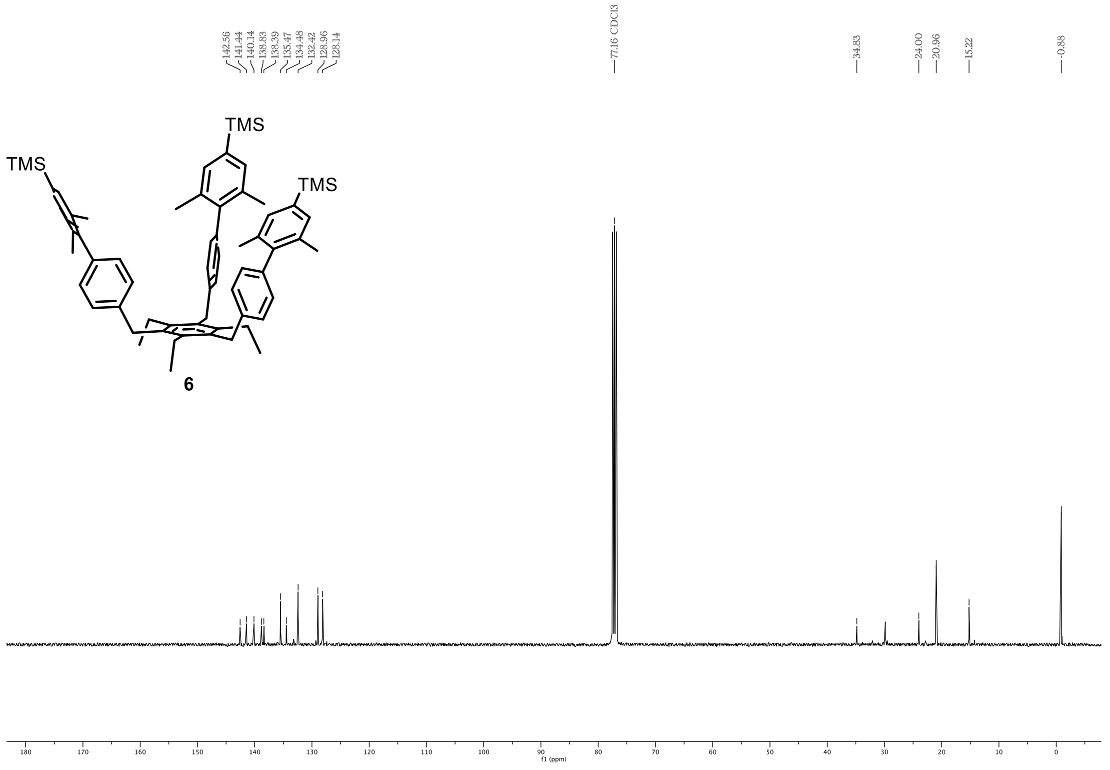

Fig. S6: ^{13}C-NMR (101 MHz, $CDCl_3$) spectrum of (((2,4,6-triethylbenzene-1,3,5-triyl)tris(methylene))tris(2,6-dimethyl-[1,1'-biphenyl]-4',4-diyl))tris(trimethylsilane)(**6**).

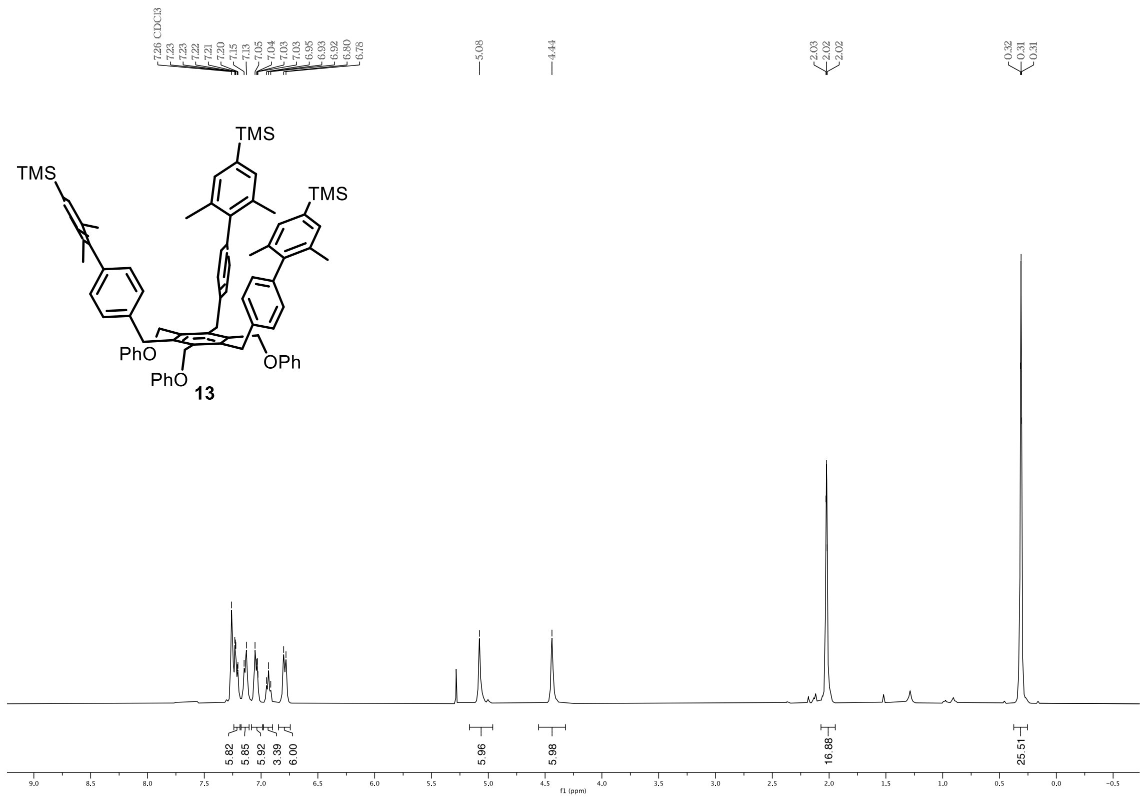

Fig. S7: ^{1}H-NMR (400 MHz, $CDCl_3$) spectrum of (((2,4,6-tris(phenoxymethyl)benzene-1,3,5-triyl)tris(methylene))tris(2,6-dimethyl-[1,1'-biphenyl]-4',4-diyl))tris(trimethylsilane) (**13**).

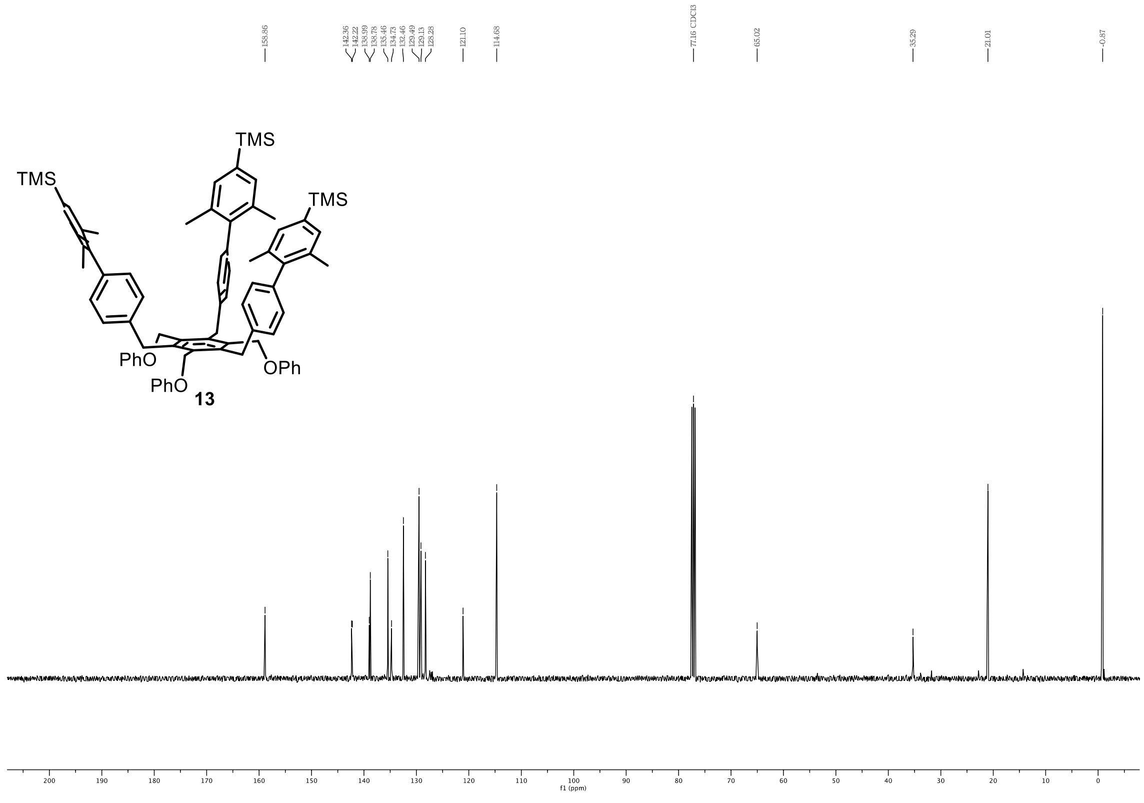

Fig. S8: ^{13}C-NMR (101 MHz, $CDCl_3$) spectrum of (((2,4,6-tris(phenoxymethyl)benzene-1,3,5-triyl)tris(methylene))tris(2,6-dimethyl-[1,1'-biphenyl]-4',4-diyl))tris(trimethylsilane) (**13**).

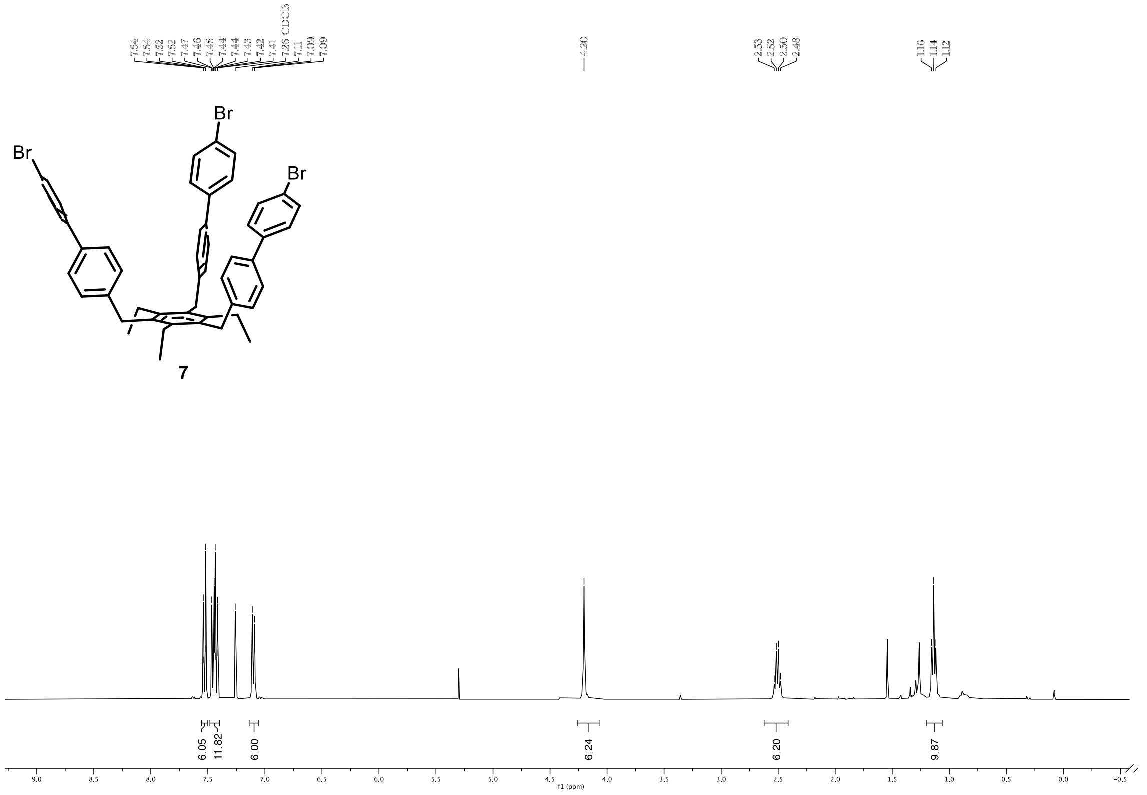

Fig. S9: ^{1}H-NMR (400 MHz, $CDCl_3$) spectrum of 4',4''',4'''''-((2,4,6-triethylbenzene-1,3,5-triyl)tris(methylene))tris(4-bromo-1,1'-bi-phenyl) (**7**).

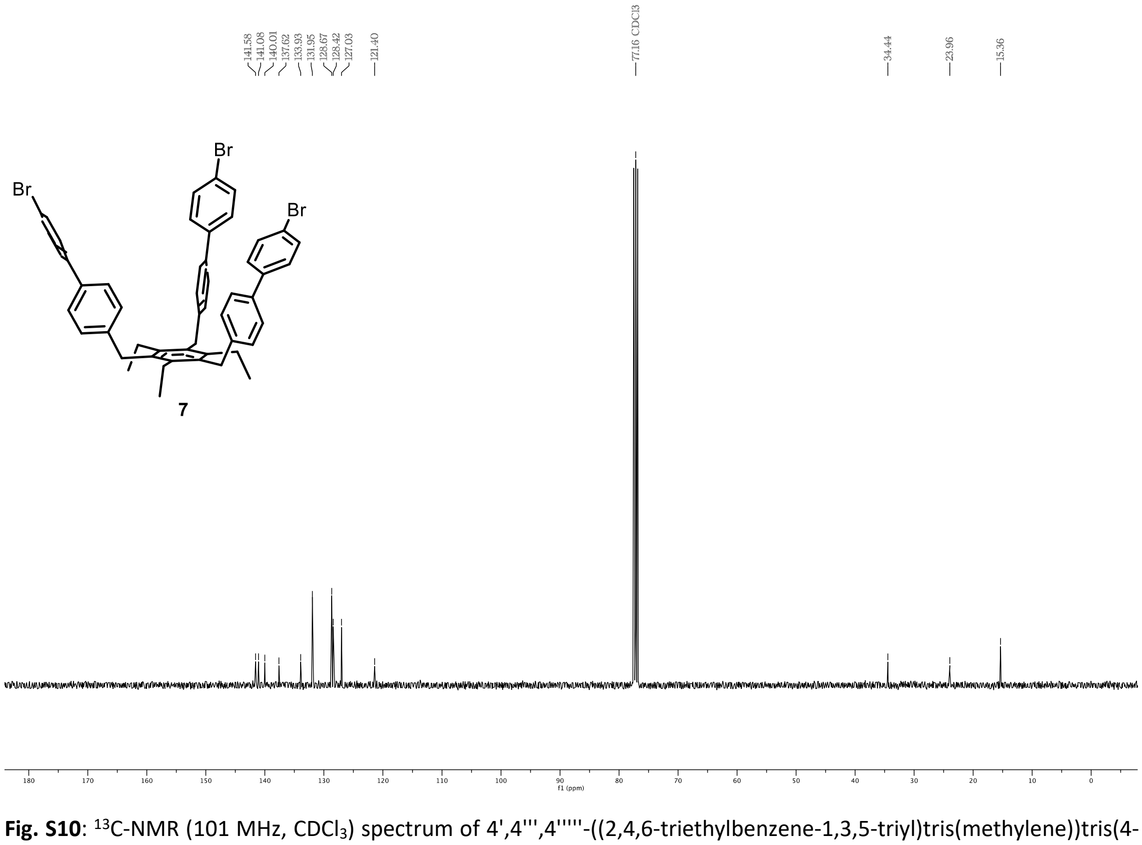

Fig. S10: ^{13}C-NMR (101 MHz, $CDCl_3$) spectrum of 4',4''',4'''''-((2,4,6-triethylbenzene-1,3,5-triyl)tris(methylene))tris(4-bromo-1,1'-bi-phenyl) (**7**).

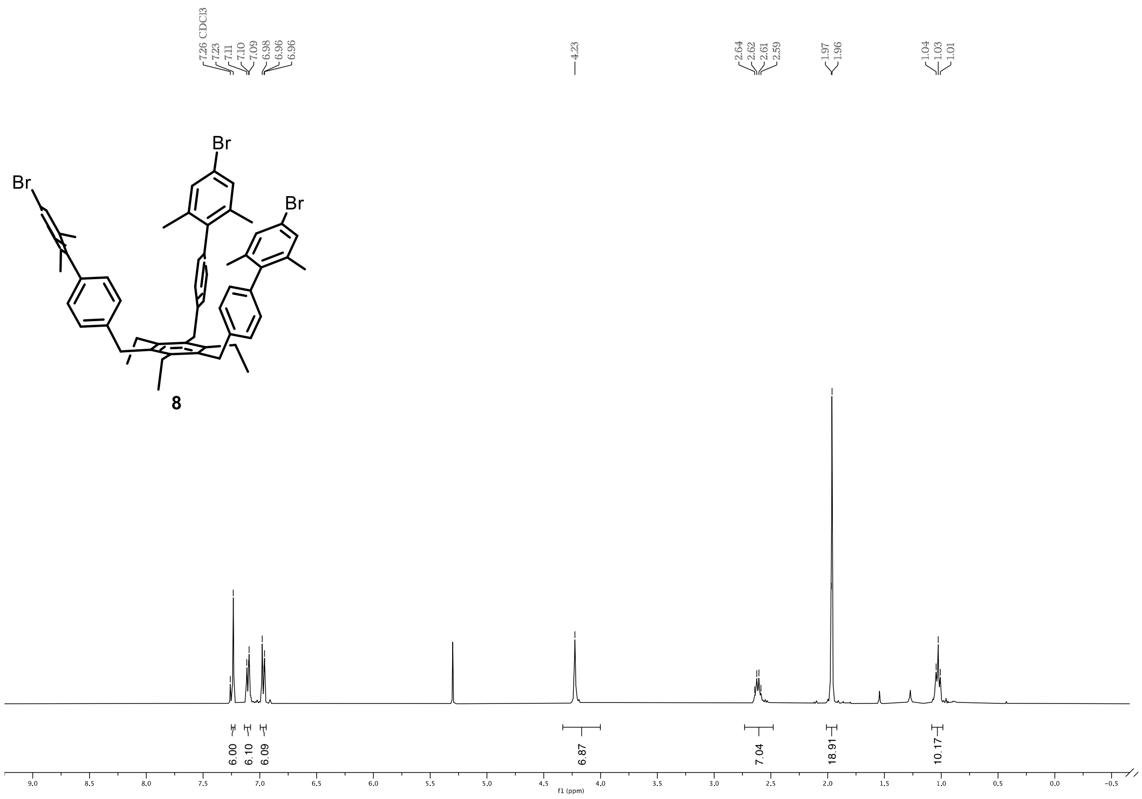

Fig. S11: ^{1}H-NMR (400 MHz, $CDCl_3$) spectrum of 4',4''',4'''''-((2,4,6-triethylbenzene-1,3,5-triyl)tris(methylene))-tris(4-bromo-2,6-di-methyl-1,1'-biphenyl) (**8**).

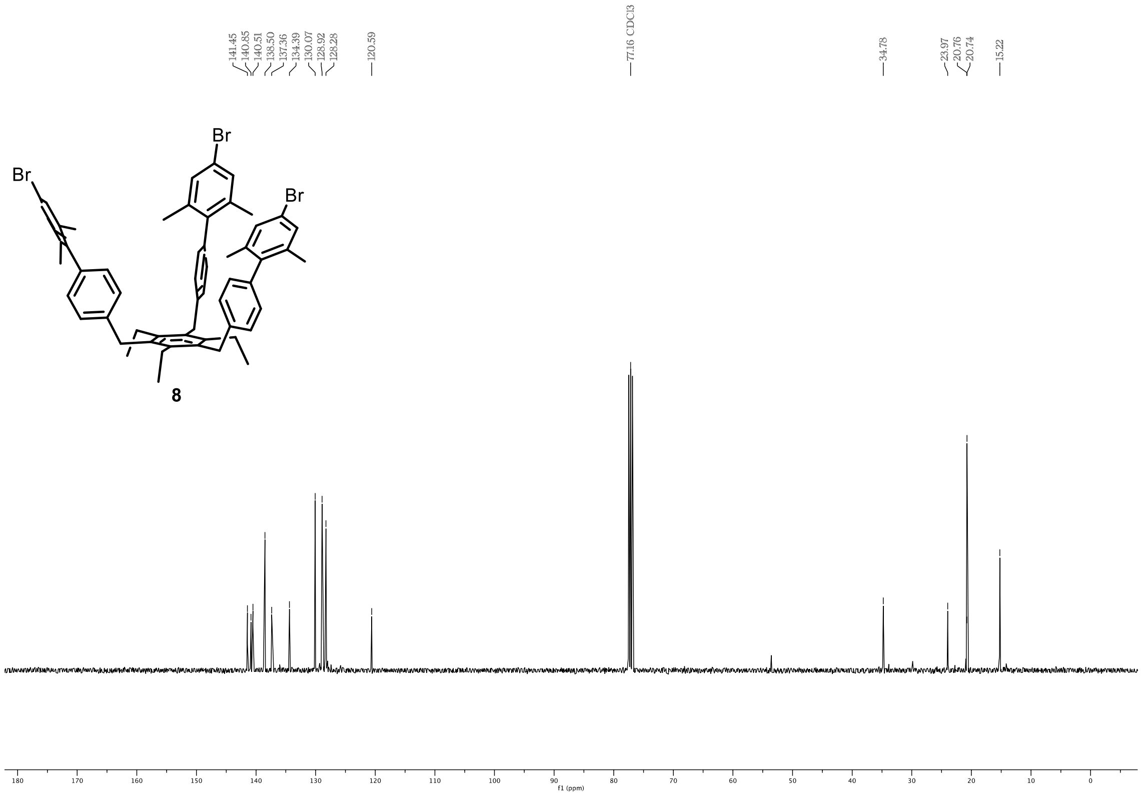

Fig. S12: ^{13}C-NMR (101 MHz, $CDCl_3$) spectrum of 4',4''',4'''''-((2,4,6-triethylbenzene-1,3,5-triyl)tris(methylene))-tris(4-bromo-2,6-di-methyl-1,1'-biphenyl) (**8**).

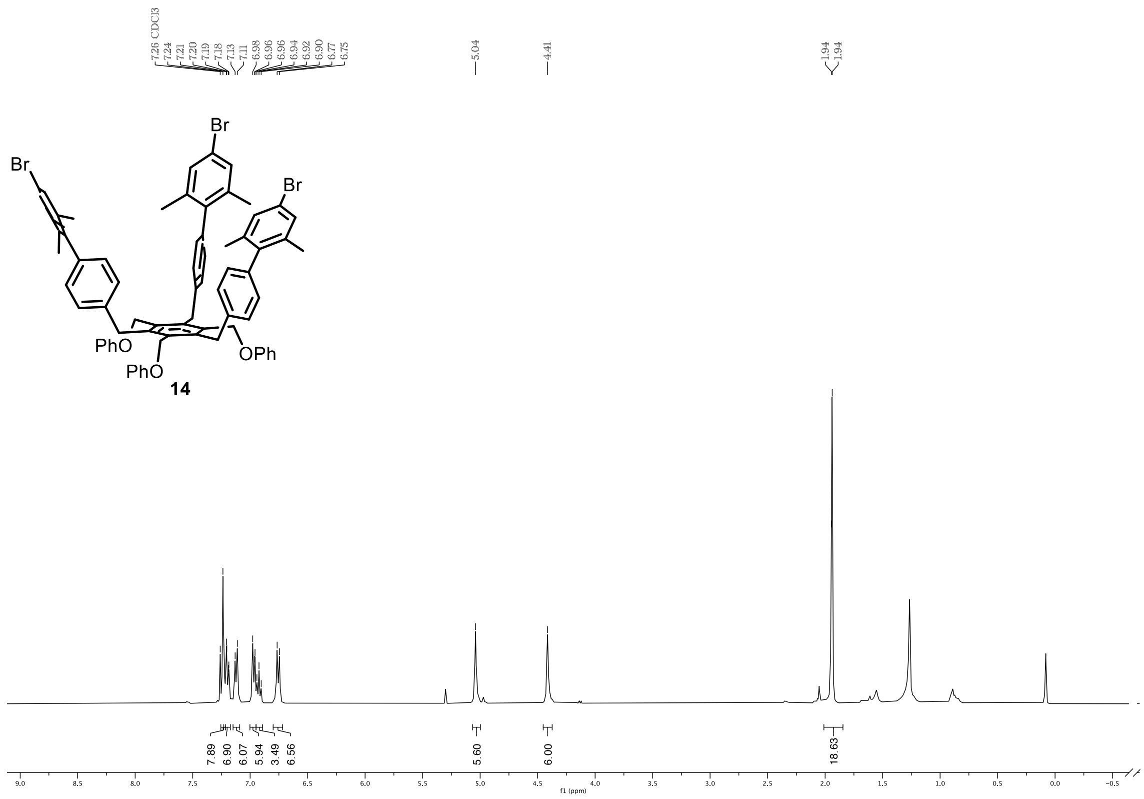

Fig. S13: ^{1}H-NMR (400 MHz, $CDCl_3$) spectrum of 4',4''',4'''''-((2,4,6-tris(phenoxymethyl)benzene-1,3,5-triyl)tris(methylene))tris(4-bromo-2,6-dimethyl-1,1'-biphenyl) (**14**).

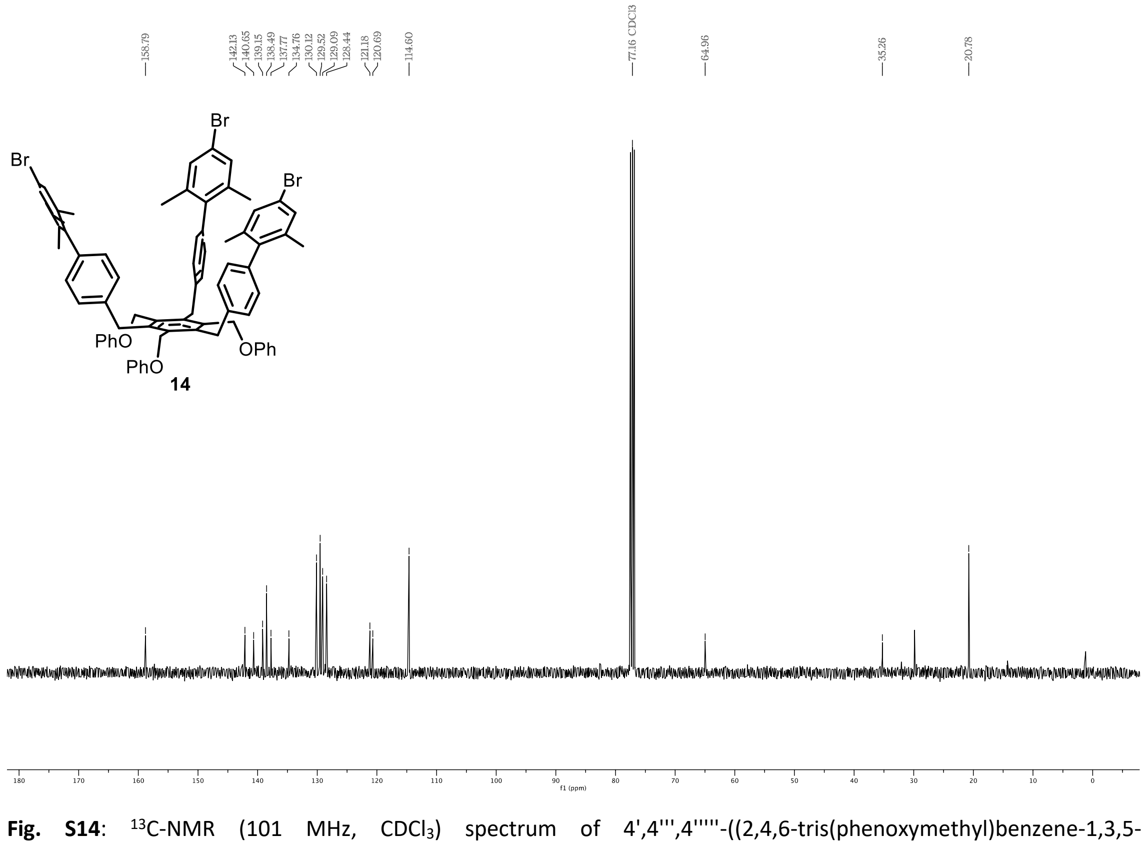

Fig. S14: ^{13}C-NMR (101 MHz, $CDCl_3$) spectrum of 4',4''',4'''''-((2,4,6-tris(phenoxymethyl)benzene-1,3,5-triyl)tris(methylene))tris(4-bromo-2,6-dimethyl-1,1'-biphenyl) (**14**).

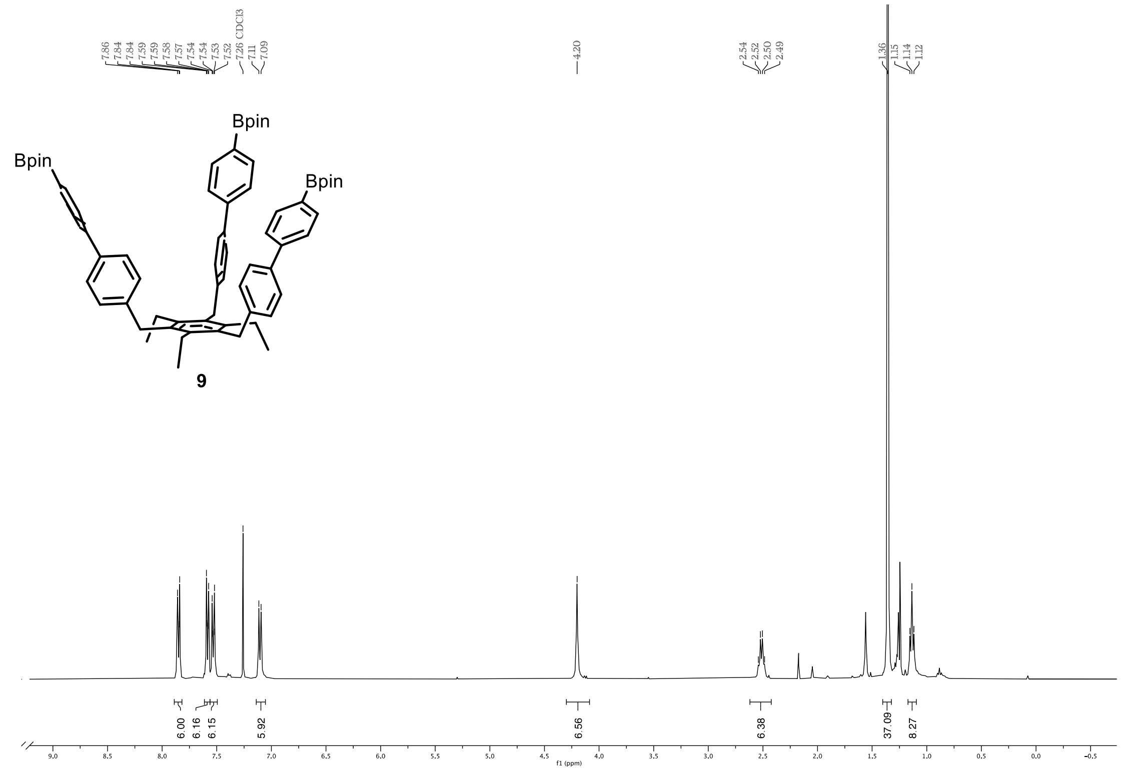

Fig. S15: ^{1}H-NMR (400 MHz, $CDCl_3$) spectrum of 2,2',2''-(((2,4,6-triethylbenzene-1,3,5-triyl)tris(methylene))tris-([1,1'-biphenyl]-4',4-diyl))tris(4,4,5,5-tetramethyl-1,3,2-dioxaborolane) (**9**).

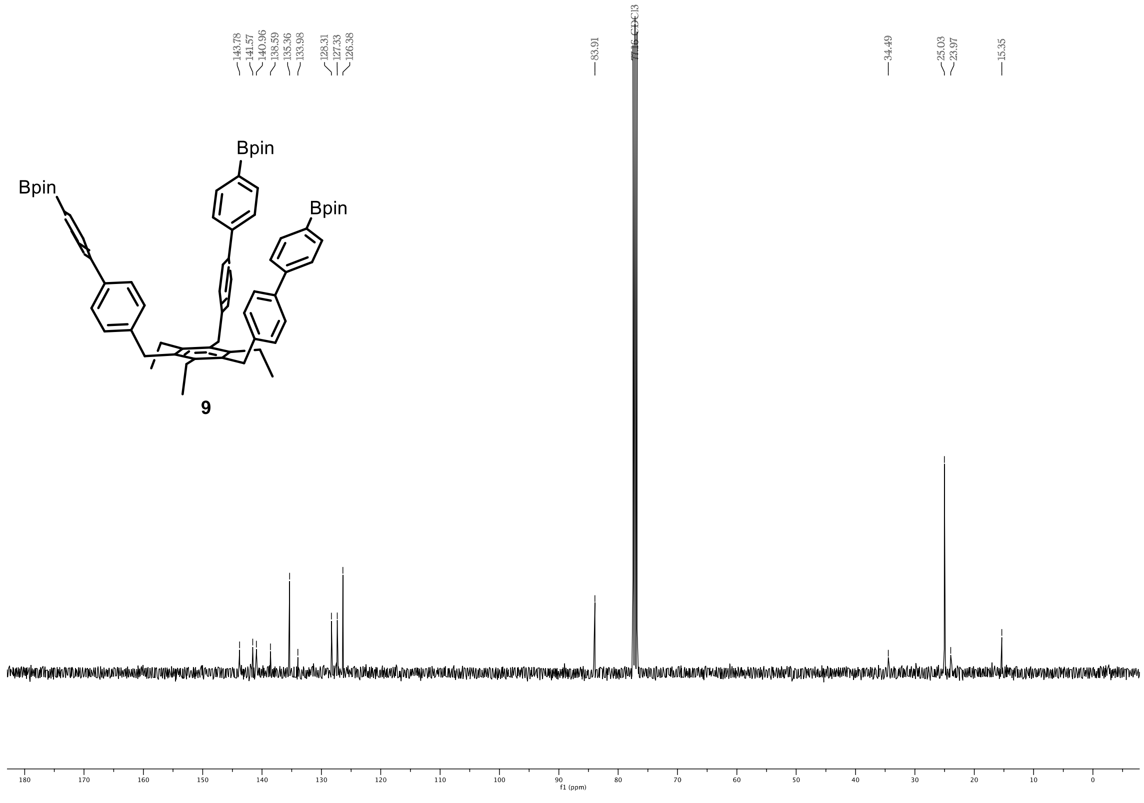

Fig. S16: ^{13}C-NMR (101 MHz, $CDCl_3$) spectrum of 2,2',2''-(((2,4,6-triethylbenzene-1,3,5-triyl)tris(methylene))tris-([1,1'-biphenyl]-4',4-diyl))tris(4,4,5,5-tetramethyl-1,3,2-dioxaborolane) (**9**).

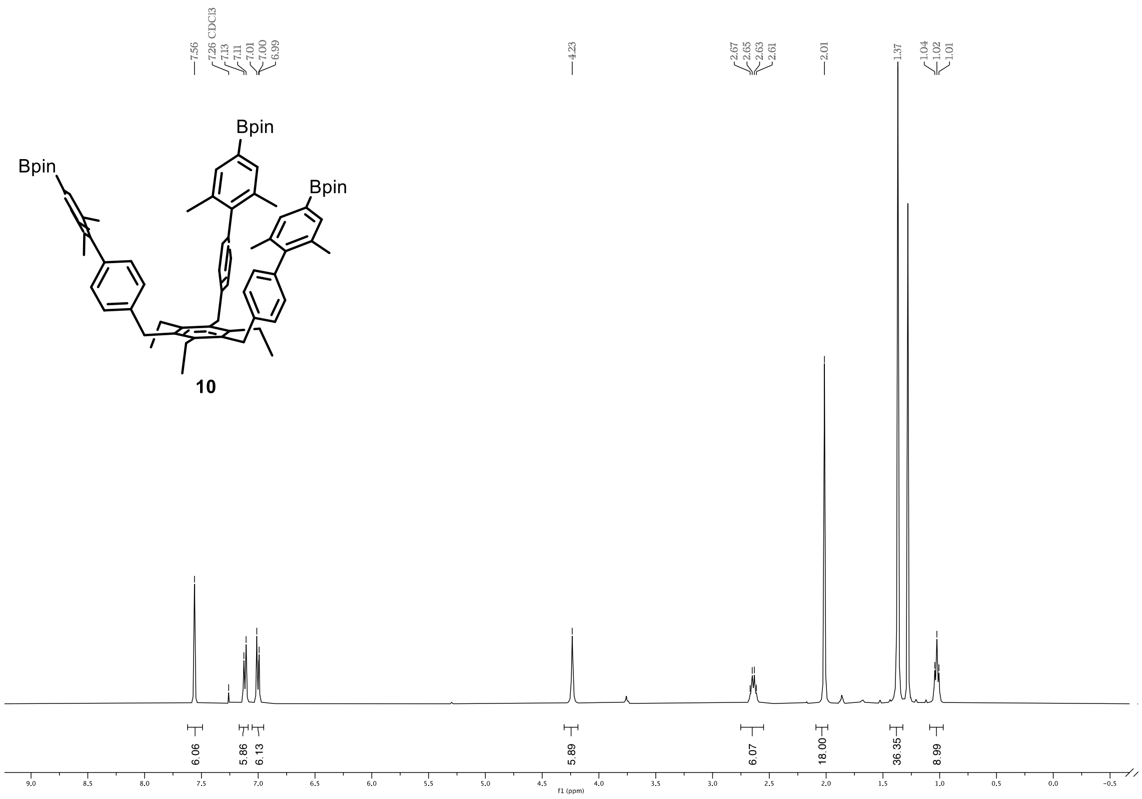

Fig. S17: ^{1}H-NMR (400 MHz, $CDCl_3$) spectrum of 2,2',2''-(((2,4,6-triethylbenzene-1,3,5-triyl)tris(methylene))tris-(2,6-dimethyl-[1,1'-biphenyl]-4',4-diyl))tris(4,4,5,5-tetramethyl-1,3,2-dioxaborolane) (**10**).

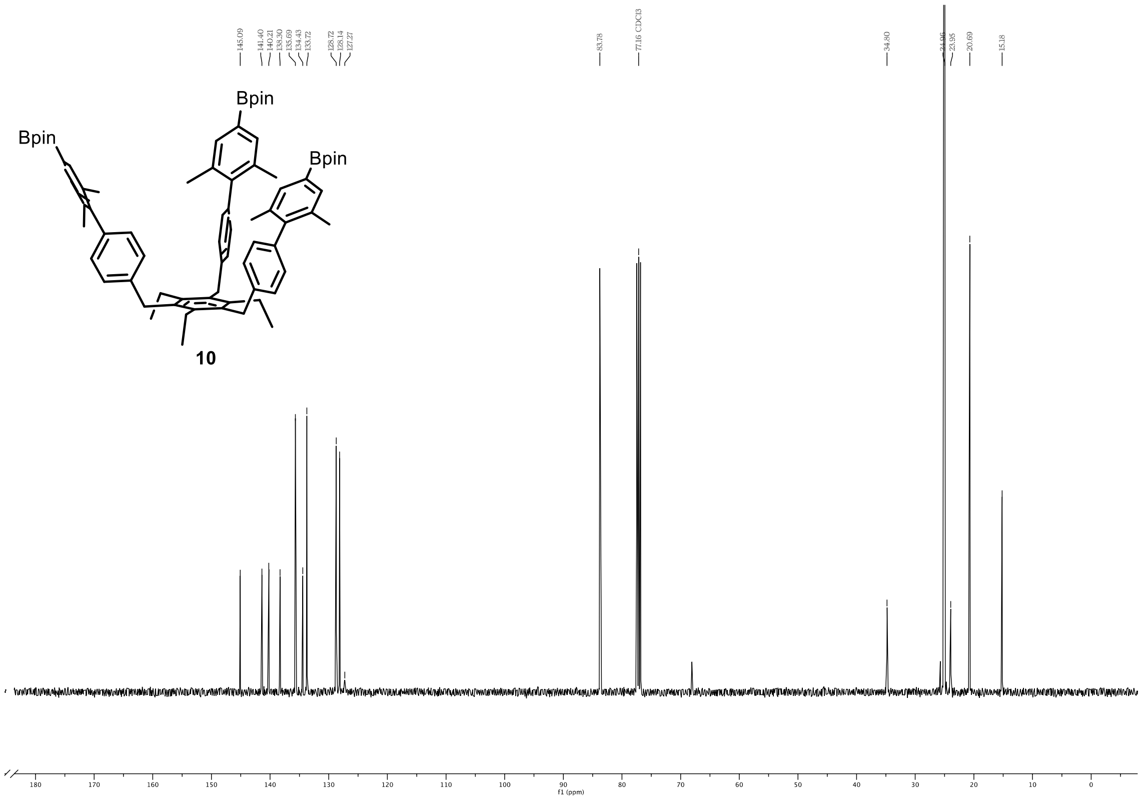

Fig. S18: ^{13}C-NMR (101 MHz, $CDCl_3$) spectrum of 2,2',2''-(((2,4,6-triethylbenzene-1,3,5-triyl)tris(methylene))tris-(2,6-dimethyl-[1,1'-biphenyl]-4',4-diyl))tris(4,4,5,5-tetramethyl-1,3,2-dioxaborolane) (**10**).

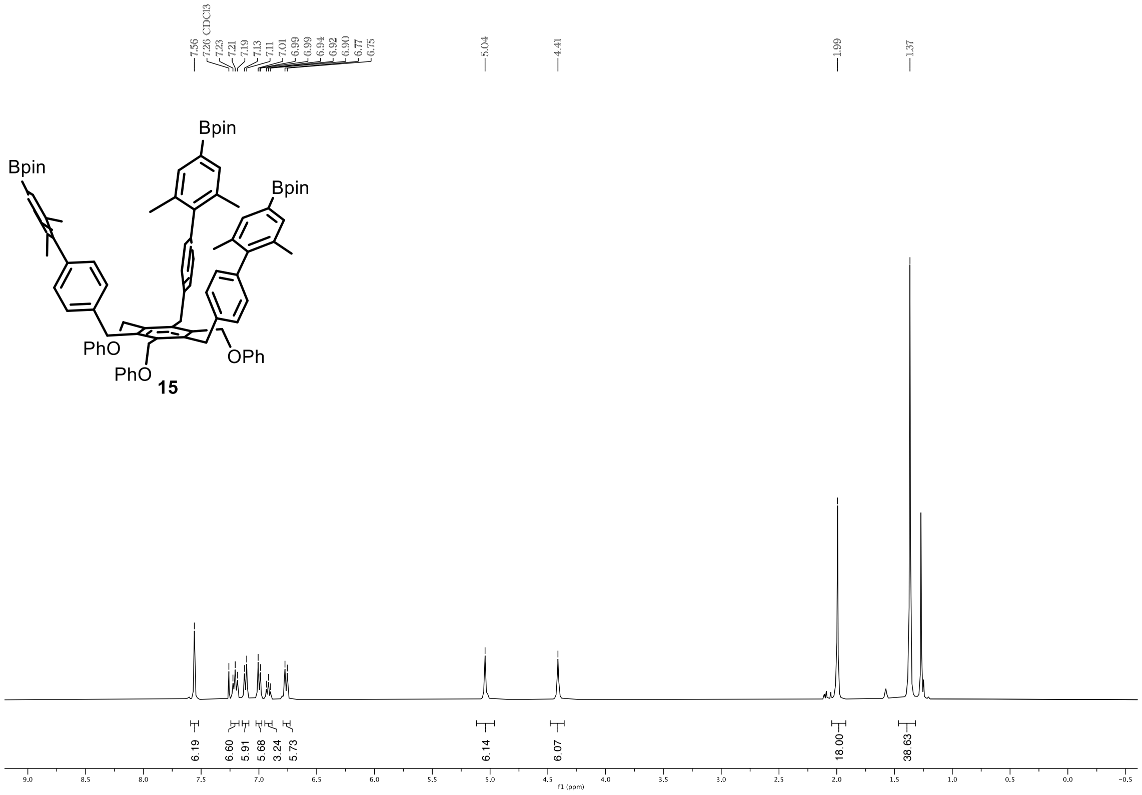

Fig. S19: ^{1}H-NMR (400 MHz, $CDCl_3$) spectrum of 2,2',2''-(((2,4,6-tris(phenoxymethyl)benzene-1,3,5-triyl)tris(methylene))tris(2,6-dimethyl-[1,1'-biphenyl]-4',4-diyl))tris(4,4,5,5-tetramethyl-1,3,2-dioxaborolane) (**15**).

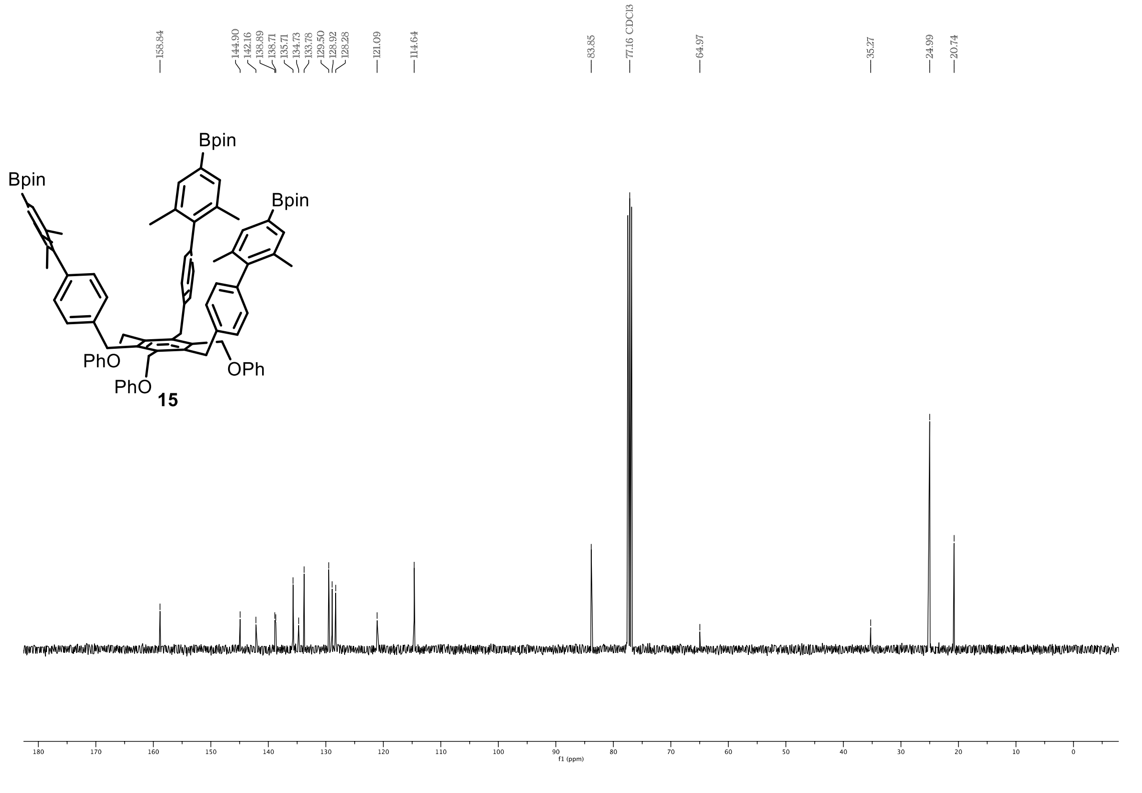

Fig. S20: ^{13}C-NMR (101 MHz, $CDCl_3$) spectrum of 2,2',2''-(((2,4,6-tris(phenoxymethyl)benzene-1,3,5-triyl)tris(methylene))tris(2,6-dimethyl-[1,1'-biphenyl]-4',4-diyl))tris(4,4,5,5-tetramethyl-1,3,2-dioxaborolane) (**15**).

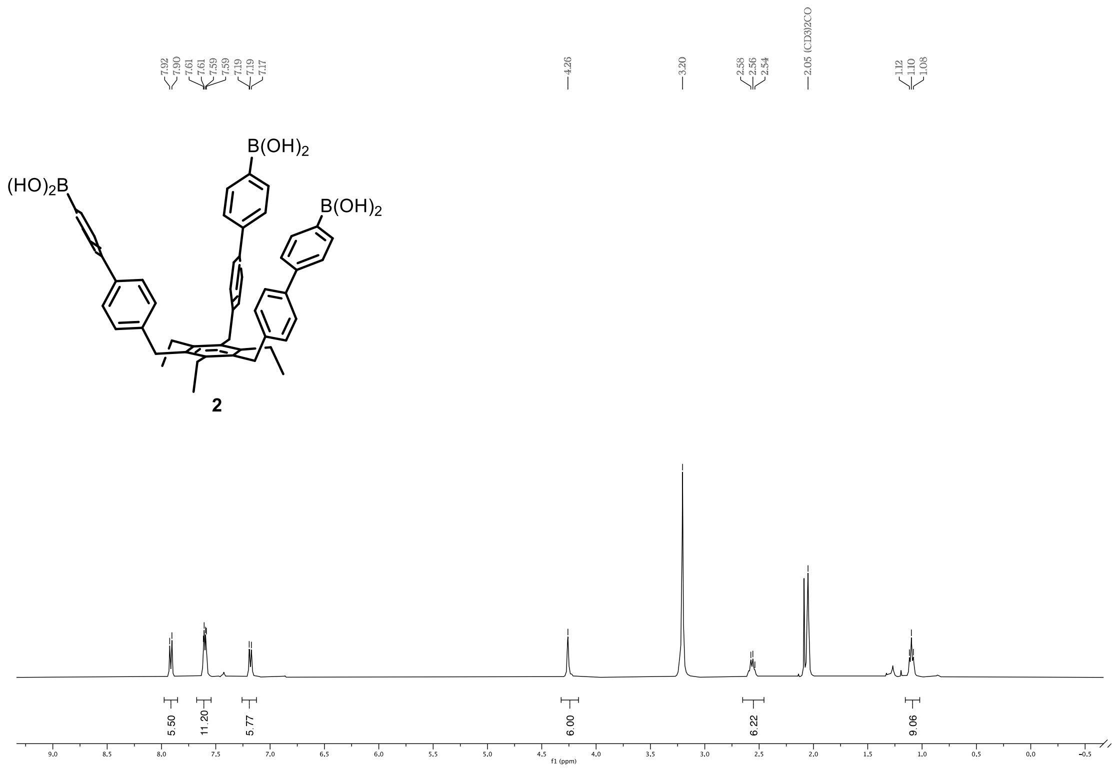

Fig. S21: ^{1}H-NMR (400 MHz, acetone-d_6) spectrum of (((2,4,6-triethylbenzene-1,3,5-triyl)tris(methylene))tris([1,1'-biphenyl]-4',4-diyl))-triboronic acid (**2**) in acetone-d_6 with few drops of D_2O.

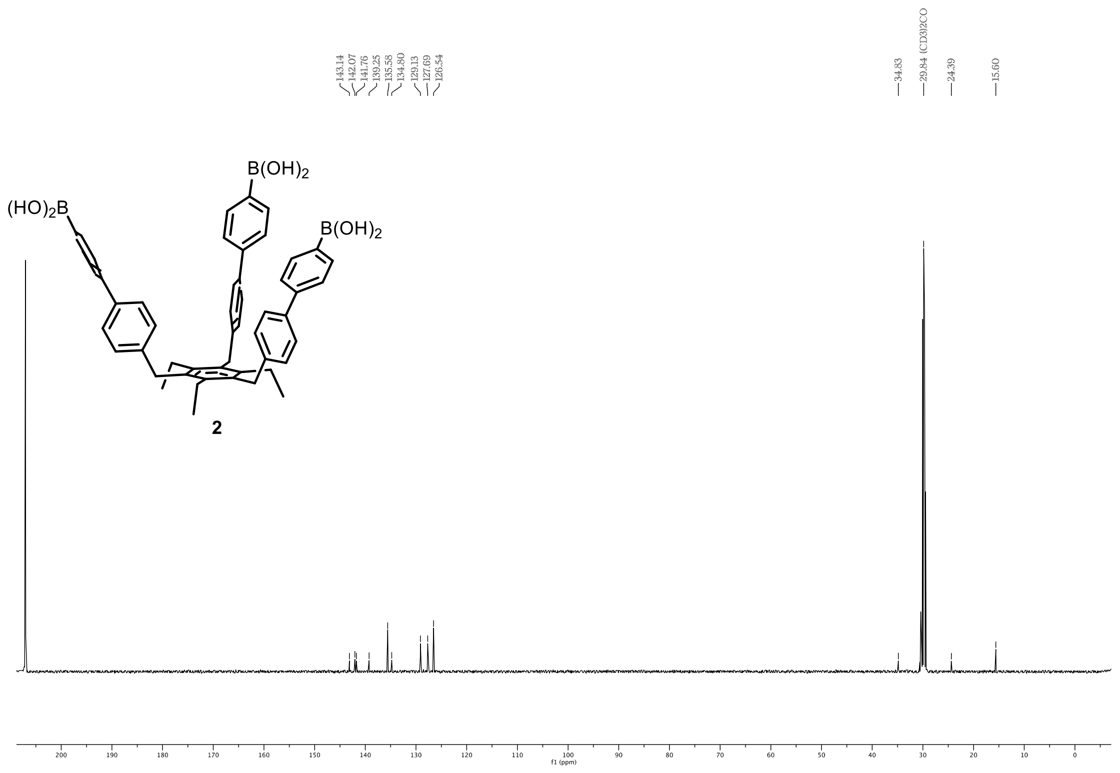

Fig. S22: ^{13}C-NMR (101 MHz, acetone-d_6) spectrum of (((2,4,6-triethylbenzene-1,3,5-triyl)tris(methylene))tris-([1,1'-biphenyl]-4',4-diyl))-triboronic acid (**2**) in acetone-d_6 with few drops of D_2O.

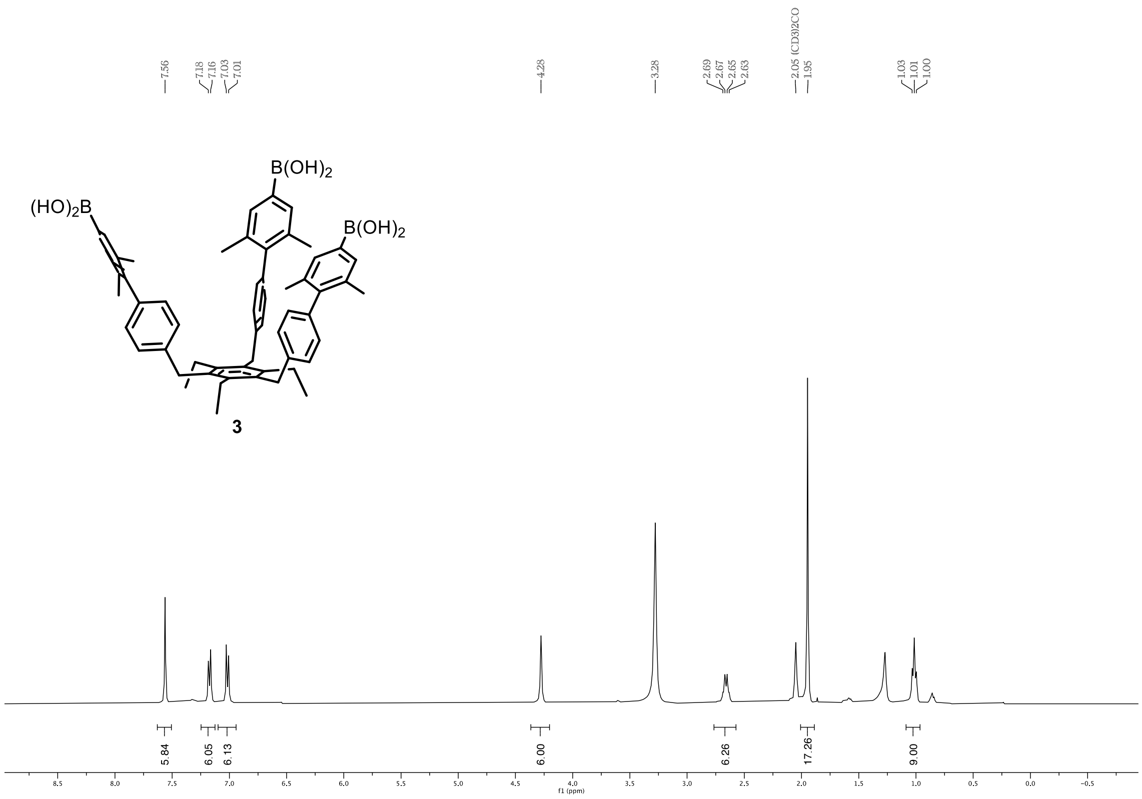

Fig. S23: ^{1}H-NMR (400 MHz, acetone-d$_6$) spectrum of (((2,4,6-triethylbenzene-1,3,5-triyl)tris(methylene))tris(2,6-dimethyl-[1,1'-biphen-yl]-4',4-diyl))triboronic acid (**3**) in acetone-d$_6$ with few drops of D_2O.

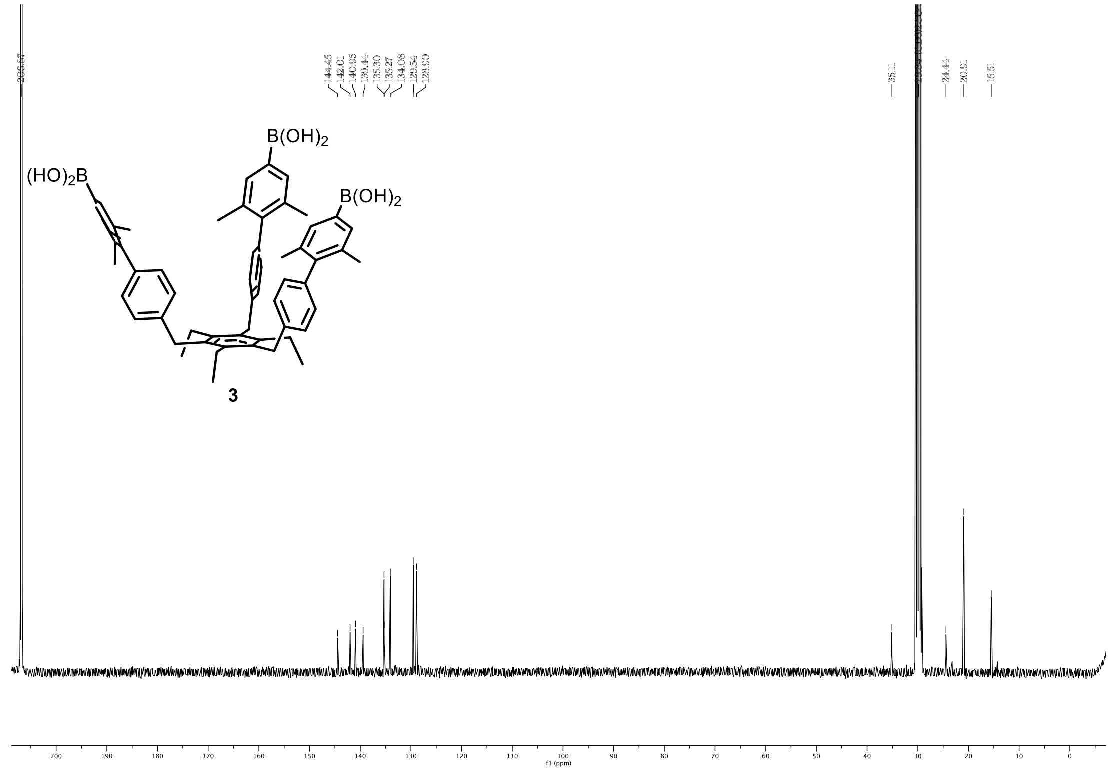

Fig. S24: ^{13}C-NMR (101 MHz, acetone-d$_6$) spectrum of (((2,4,6-triethylbenzene-1,3,5-triyl)tris(methylene))tris(2,6-dimethyl-[1,1'-biphen-yl]-4',4-diyl))triboronic acid (**3**) in acetone-d$_6$ with few drops of D_2O.

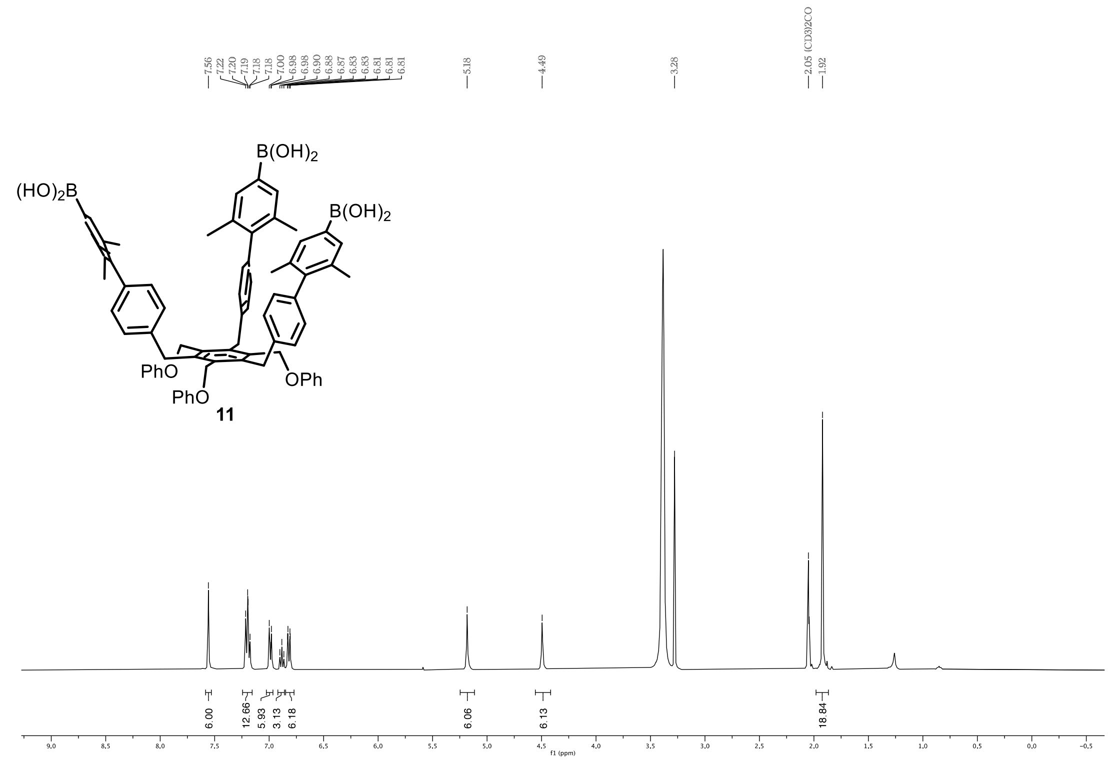

Fig. S25: ^{1}H-NMR (400 MHz, acetone-d_6) spectrum of 2,2',2''-(((2,4,6-tris(phenoxymethyl)benzene-1,3,5-triyl)tris(methylene))tris(2,6-dimethyl-[1,1'-biphenyl]-4',4-diyl))tris(4,4,5,5-tetramethyl-1,3,2-dioxaborolane) (**11**) in acetone-d_6 with few drops of D_2O.

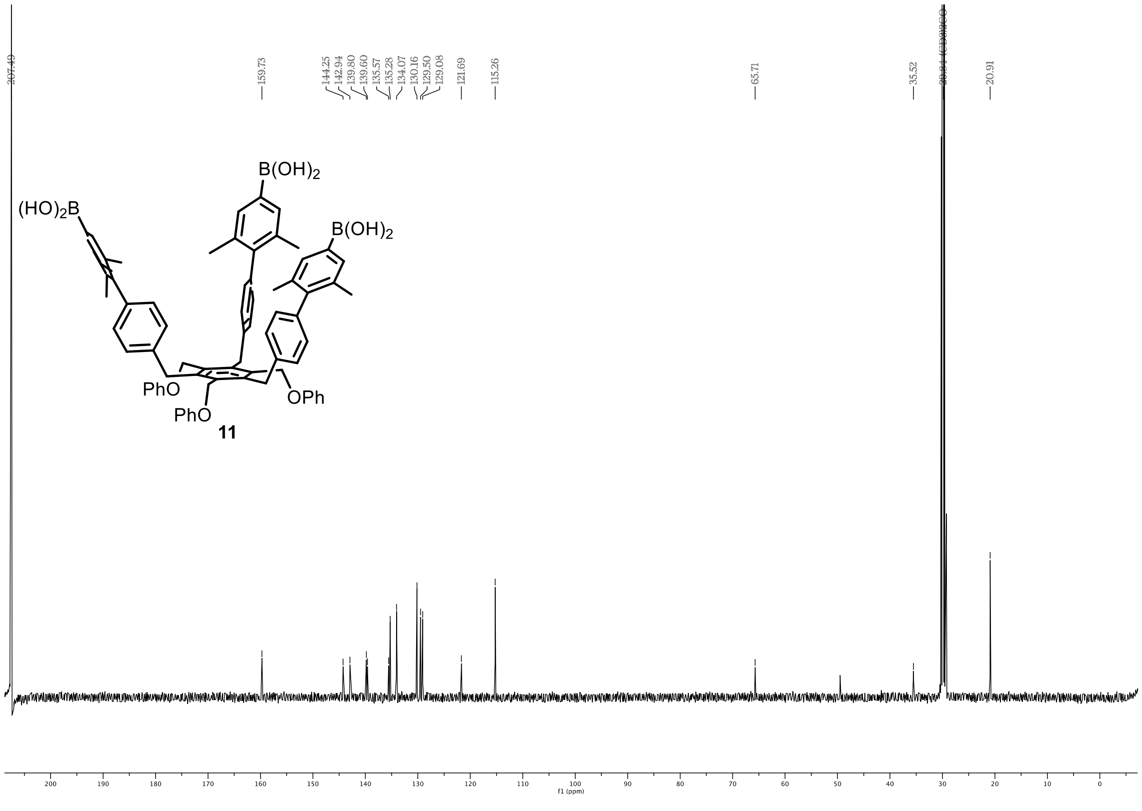

Fig. S26: ^{13}C-NMR (101 MHz, acetone-d_6) spectrum of 2,2',2''-(((2,4,6-tris(phenoxymethyl)benzene-1,3,5-triyl)tris(methylene))tris(2,6-dimethyl-[1,1'-biphenyl]-4',4-diyl))tris(4,4,5,5-tetramethyl-1,3,2-dioxaborolane) (**11**) in acetone-d_6 with few drops of D_2O.

4. Synthesis and Characterization of boroxine Cages

General procedure for the formation of boroxine cages in a sealed tube: A two-necked pressure tube equipped with a magnetic stirring bar was charged with triboronic acid (1.0 equiv) and H_2O (2.0 equiv per boronic acid moiety), and was evacuated and back-filled with N_2 for five times. Subsequently, anhydrous $CDCl_3$ was added under positive N_2 pressure. The tube was sealed tight, and heated to 110 °C. The reaction was monitored by extracting a 0.5 mL aliquot and direct analysis via ^1H-NMR. Upon completion, the yield was determined by adding 1,1,2,2-tetrachloroethane (TCE) as an internal standard and recording a quantitative proton NMR spectrum.

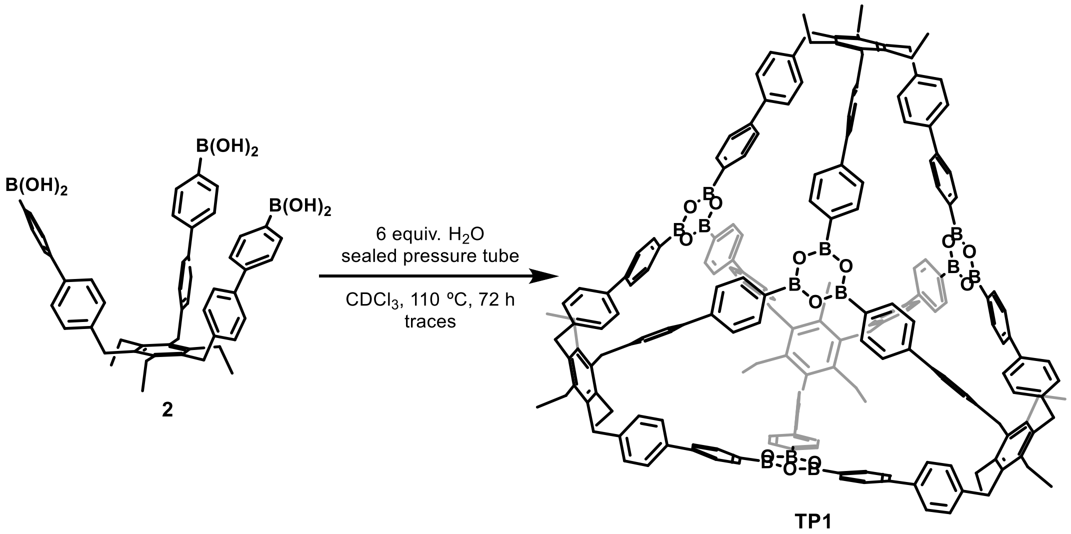

Boroxine Cage TP1. General procedure for the formation of boroxine cages was applied to triboronic acid precursor **2** (7.60 mg, 9.59 µmol, 1.0 equiv, 1.5 equiv) using 1.9 mL anhydrous $CDCl_3$ (0.005 M) with H_2O (1.0 µL H_2O, 55.6 µmol, 6 equiv). Reaction control was carried out after three and seven days.

HRMS (MALDI-TOF+, DCTB, AgTFA) *m/z* calcd. for $^{12}C_{202}{}^{13}C_2H_{180}{}^{10}B_4{}^{11}B_8O_{12}{}^{109}Ag$ [M+Ag]$^+$ 3060.3851, found 3060.3972.

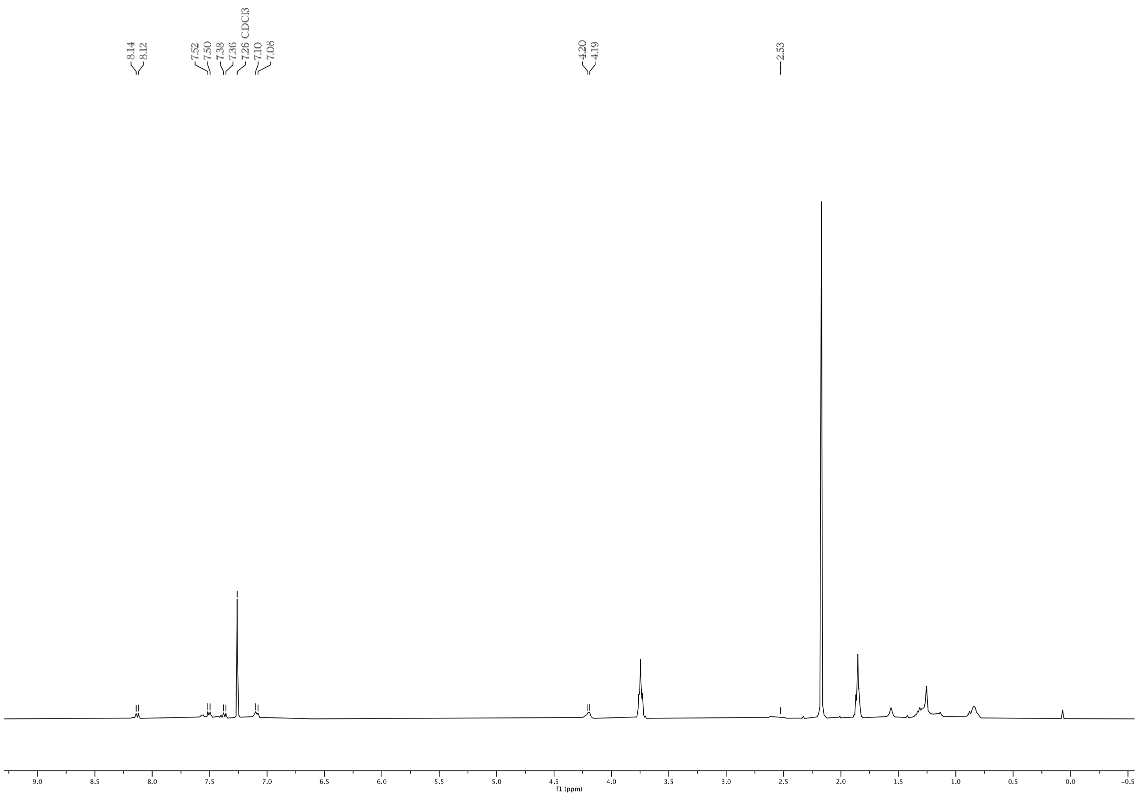

Fig. S27: ^{1}H-NMR (400 MHz, $CDCl_3$) spectrum of the trial of boroxine cage formation **TP1** via auto-condensation of **2b** recorded after 7 days.

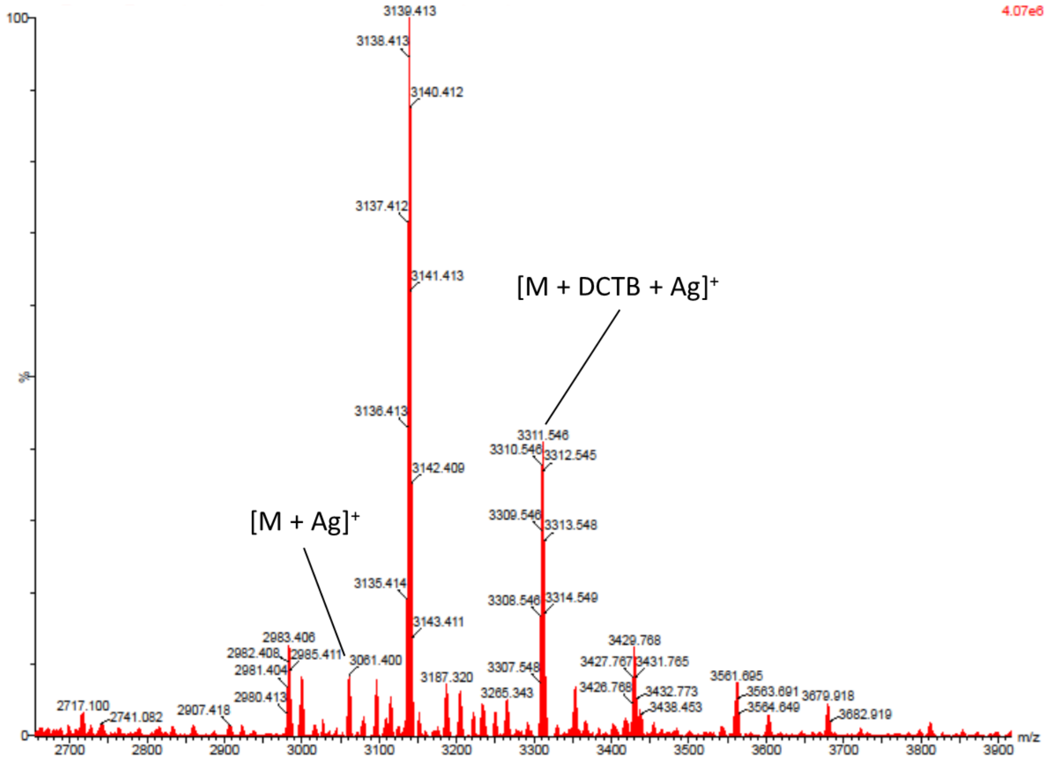

Fig. S28: HR-MALDI-TOF-MS spectrum of boroxine cage **TP1**.

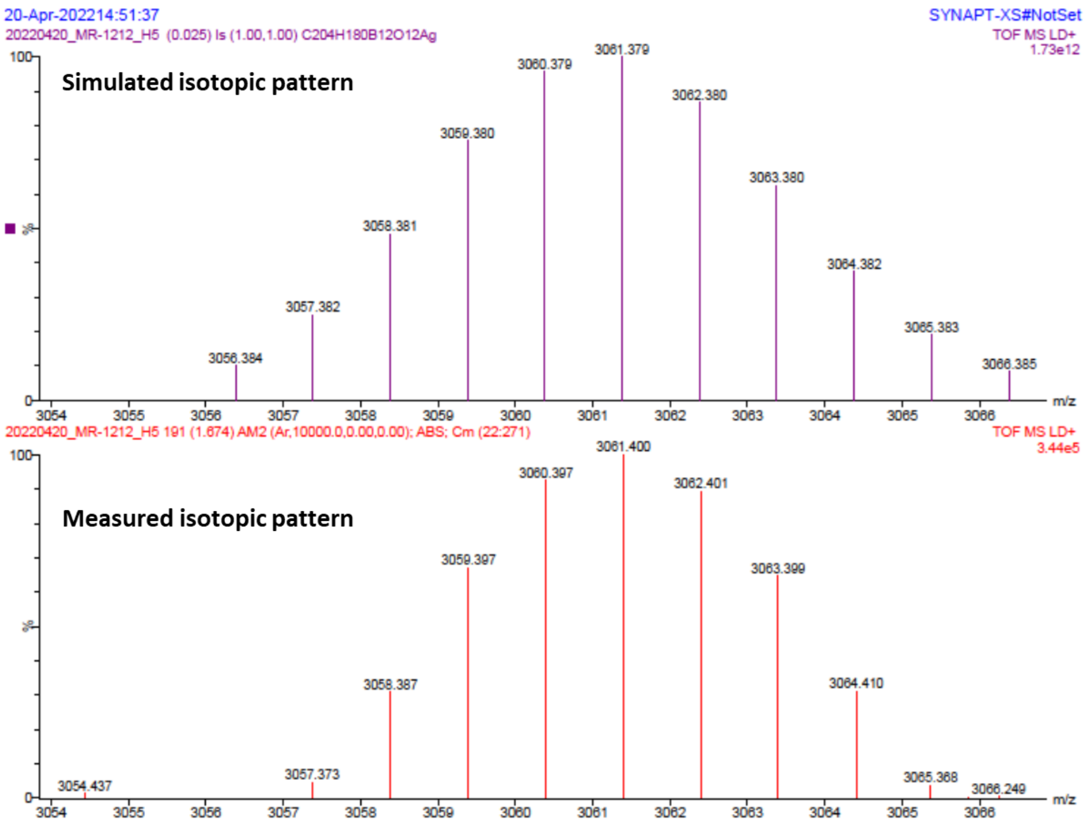

Fig. S29: Simulated (top) and measured (bottom) isotopic pattern for **TP1** with MALDI-TOF-MS.

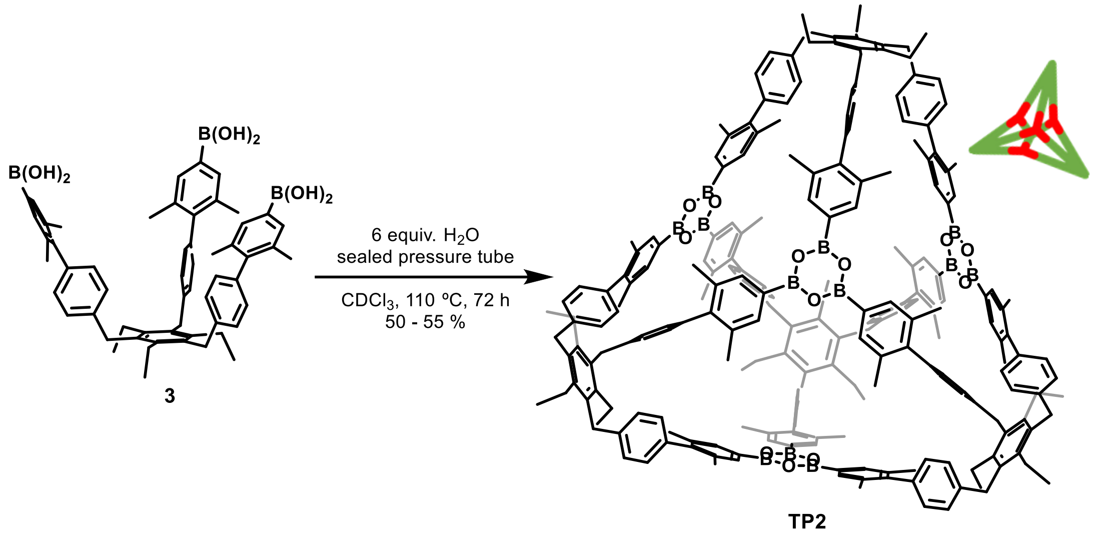

Boroxine Cage TP2 at 0.005 M. General procedure for the formation of boroxine cages was applied to triboronic acid precursor **3** (6.40 mg, 7.30 µmol, 1.0 equiv) using 1.5 mL anhydrous $CDCl_3$ (0.005 M) with H_2O (0.79 µL, 43.8 µmol, 6 equiv). The yield (Y = 53 ± 3 %) was determined after three days using TCE as the internal standard.

When the reaction was performed at 0.0025 M, a yield of Y = 59 ± 4 % was obtained.

Boroxine Cage TP2 with molecular sieves. General procedure for the formation of boroxine cages was applied to triboronic acid precursor **3** (13.4 mg, 15.3 µmol, 1.0 equiv) using 3.0 mL anhydrous $CDCl_3$ (0.005 M) with H_2O (1.65 µL, 91.7 µmol, 6 equiv). After two nights, two spatula tips of activated 4 Å molecular sieves was added. The yield (Y = 63 ± 5 %) was determined after three days using TCE as the internal standard.

^{1}H NMR (500 MHz, $CDCl_3$) δ 7.84 (s, 24H, *H*-10), 7.08 – 6.91 (m, 48H, *H*-4/5), 4.24 (s, 24H, *H*-2), 2.60 – 2.41 (m, 24H, *H*-2'), 2.02 (s, 72H, *H*-9), 1.27 (t, *J* = 6.6 Hz, 36H, *H*-3').

^{13}C NMR (126 MHz, $CDCl_3$) δ 146.3, 141.6, 139.6, 138.6, 135.7, 134.5, 134.5, 128.4, 127.9, 74.3, 34.2, 23.7, 20.8, 15.6.[5] *the ^{13}C-signal corresponding to Ar-*C*-Bpin was not detected.

^{1}H DOSY-NMR (500 MHz, $CDCl_3$): $D = 3.25 \pm 0.06 \times 10^{-10}$ m^2/s

HRMS (MALDI-TOF+, DCTB, AgTFA) *m/z* calcd. for $C_{228}H_{228}{}^{10}B^{11}B_{11}O_{12}{}^{109}Ag$ $[M+Ag]^+$ 3397.7551, found 3397.7556.

IR (ATR, FT) *ν* (cm^{-1}) 731, 906, 1003, 1215, 1342, 1398, 1513, 1603, 2926, 2960.

UV/vis (Cyclohexane) λ_{max} (nm) 216, 241, 249.

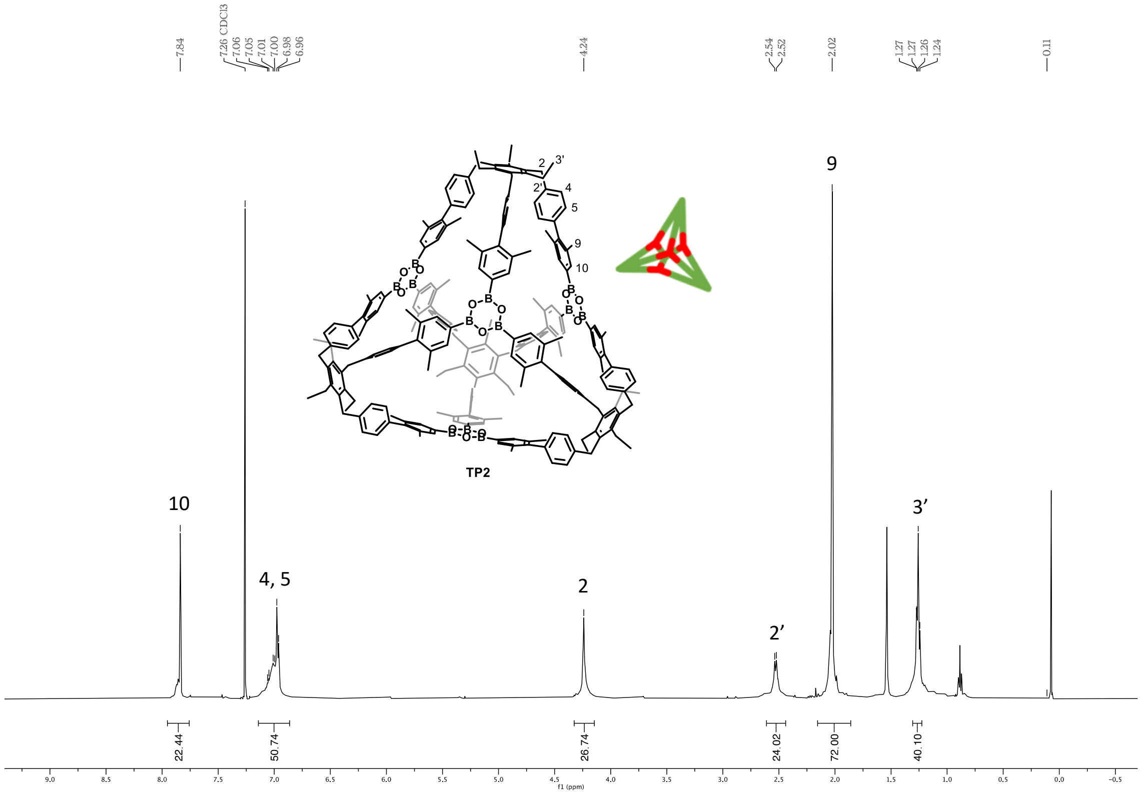

Fig. S30: ^{1}H-NMR (500 MHz, $CDCl_3$) spectrum of **TP2** via auto-condensation of **3**.

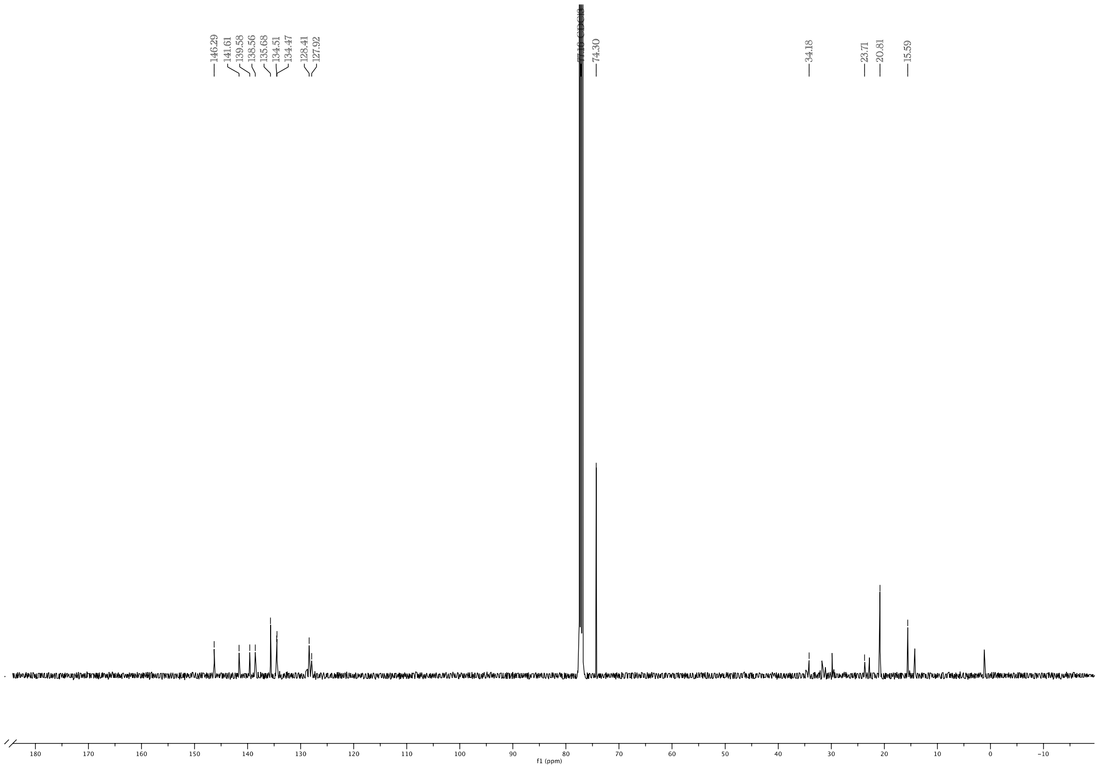

Fig. S31: ^{13}C-NMR (126 MHz, $CDCl_3$) spectrum of **TP2** via auto-condensation of **3**.

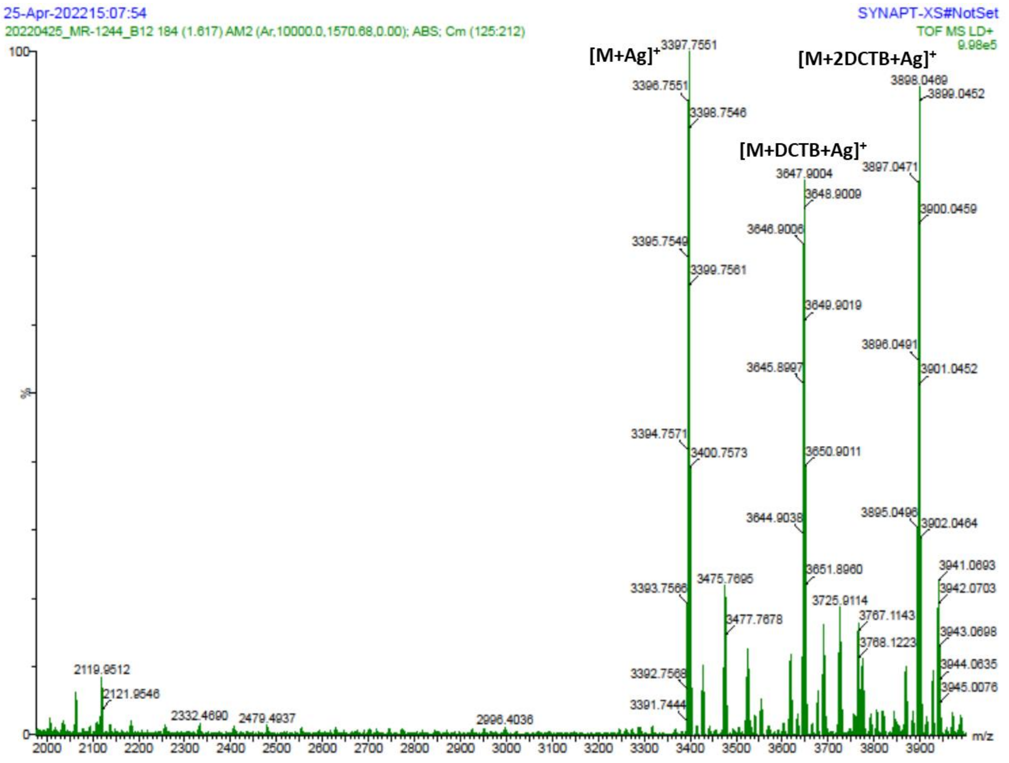

Fig. S32: HR-MALDI-TOF-MS spectrum of boroxine cage **TP2**.[6]

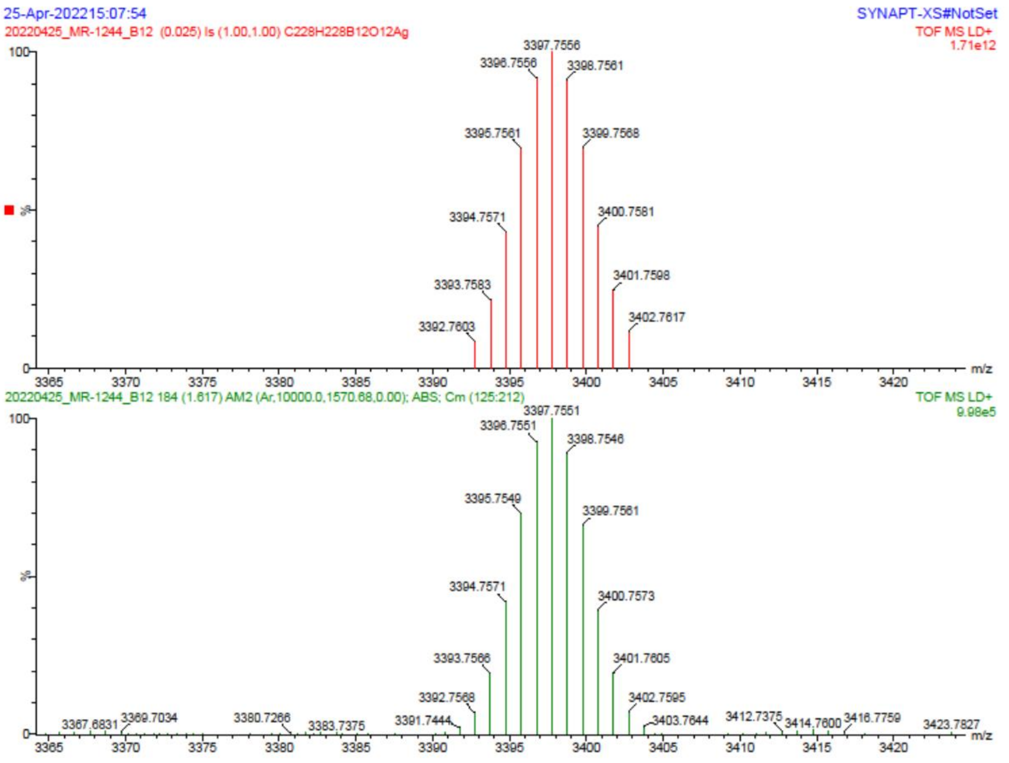

Fig. S33: Simulated (top) and measured (bottom) isotopic pattern for **TP2** with MALDI-TOF-MS.

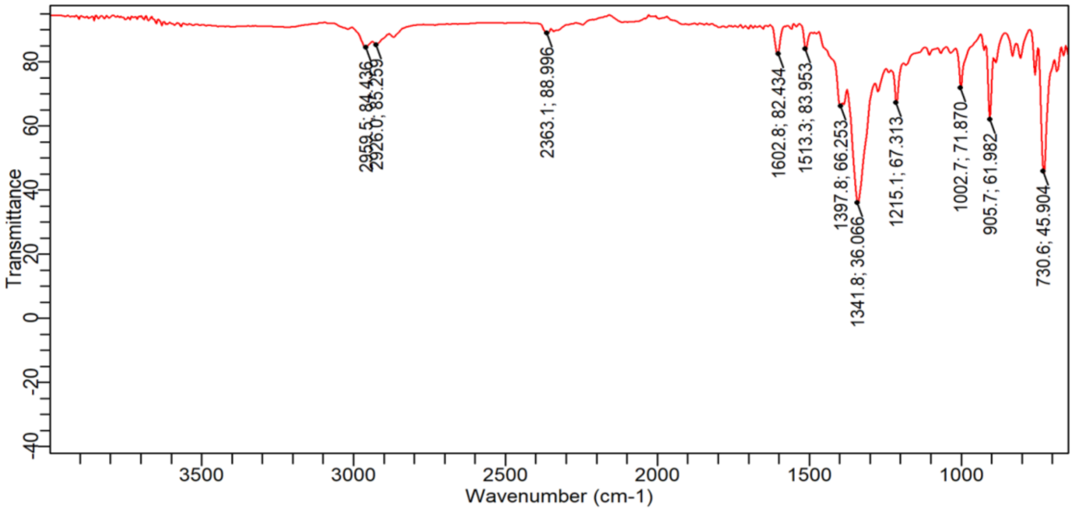

Fig. 34: FT-IR spectrum of **TP2** (ATR, diamond).

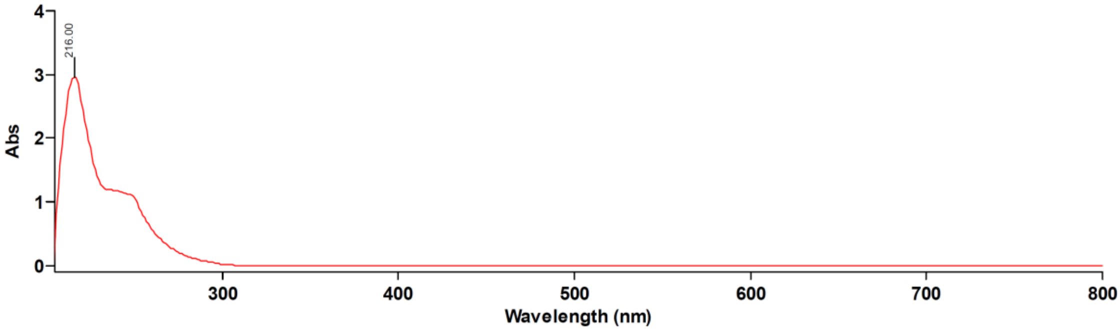

Fig. S35: UV/vis spectrum of **TP2** in cyclohexane.

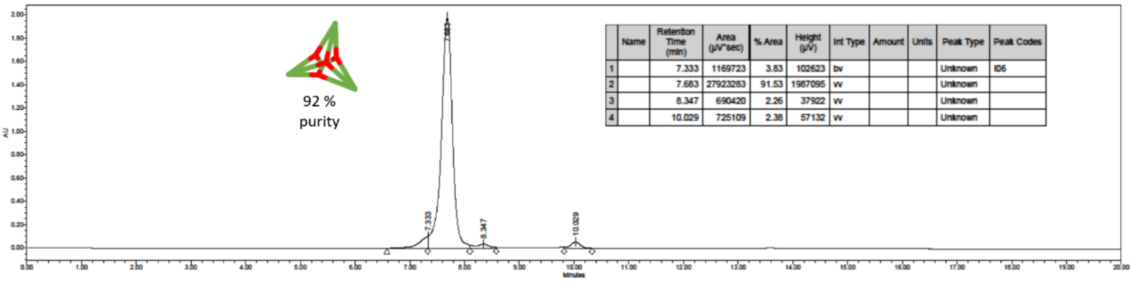

	Name	Retention Time (min)	Area (µV*sec)	% Area	Height (µV)	Int Type	Amount	Units	Peak Type	Peak Codes
1		7.333	1169723	3.83	102623	bv			Unknown	I06
2		7.683	27923283	91.53	1987095	vv			Unknown	
3		8.347	690420	2.26	37922	vv			Unknown	
4		10.029	725109	2.38	57132	vv			Unknown	

Fig. S36: GPC trace for the formation of cage **TP2** (Phenogel™ 5µm 500 Å, 300 x 7.8 mm, CH_2Cl_2 as solvent at 1 mL/min).

Yield determination of **TP2** was carried out using 1,1,2,2-tetrachloroethane (9.47 µmol) as the internal standard. The error ΔY is calculated by the standard deviation of the yields (y) of the different integrals.

Table S1: Yield determination for cage **TP2** based on quantitative ^{1}H-NMR measurements using TCE as the internal standard.

conditions	Signal [ppm]	Integral (I)	Nuclei (N)	I/N	$n_{aliquot}$ [mmol]	n_{tot} [mmol]	$n_{100\%}$ [mmol]	y [%]	ΔY [%]
**0.005 M**	7.84	0.76	24	0.03167	0.0002999	0.0008758	0.00183	48	
	6.97	1.71	48	0.03563	0.0003375	0.0009854	0.00183	54	
	4.24	0.86	24	0.03583	0.0003394	0.0009912	0.00183	54	
	2.02	2.57	72	0.03569	0.0003381	0.0009873	0.00183	54	
							**$Y_{0.005\,M}$:**	**53**	**3**
**0.0025 M**	7.84	0.43	24	0.017916667	0.00017	0.0007739	0.00143	54	
	6.97	0.98	48	0.020416667	0.00019	0.0008819	0.00143	62	
	4.24	0.45	24	0.01875	0.00018	0.0008099	0.00143	57	
	2.02	1.47	72	0.020416667	0.00019	0.0008819	0.00143	62	
							**$Y_{0.0025\,M}$:**	**59**	**4**
**Addition of 4 Å MS**	7.84	0.87	24	0.03625	0.0003434	0.0020603	0.00382	54	
	6.97	2.20	48	0.04583	0.0004342	0.0026050	0.00382	68	
	4.24	1.21	24	0.05042	0.0004776	0.0028655	0.00382	75	
	2.02	2.70	72	0.03750	0.0003552	0.0021314	0.00382	56	
							**Y_{MS}:**	**63**	**5**

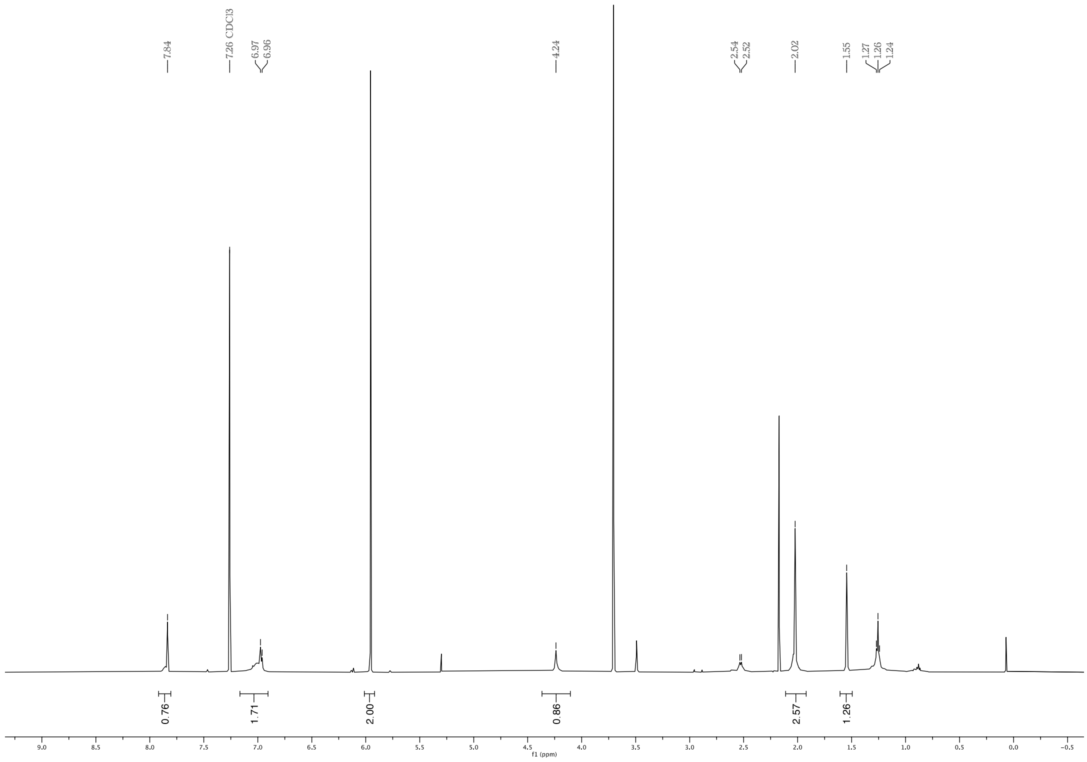

Fig. S37: Quantitative ^{1}H-NMR (500 MHz) spectrum of the self-assembly of **TP2** at 0.005 M using TCE as the IS. Only the signals used for quantification and the signal corresponding to the IS (TCE) and H_2O were integrated.

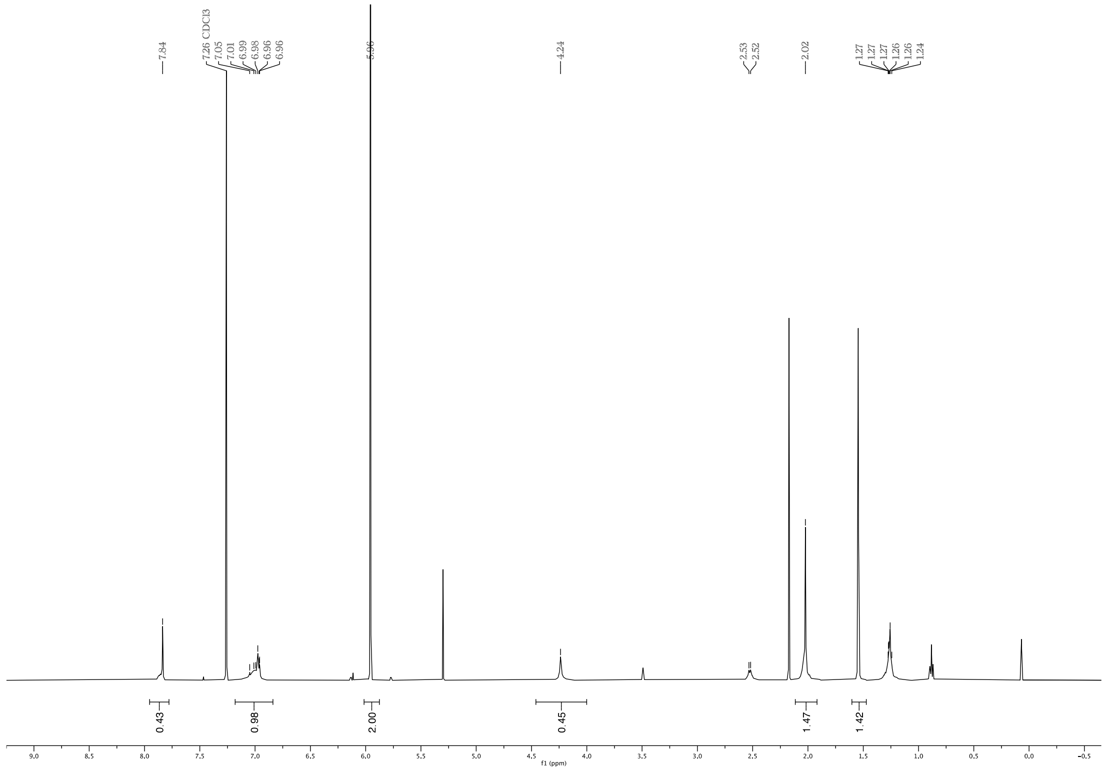

Fig. S38: Quantitative ^{1}H-NMR (500 MHz) spectrum of the self-assembly of **TP2** at 0.0025 M using TCE as the IS. Only the signals used for quantification and the signal corresponding to the IS (TCE) and H_2O were integrated.

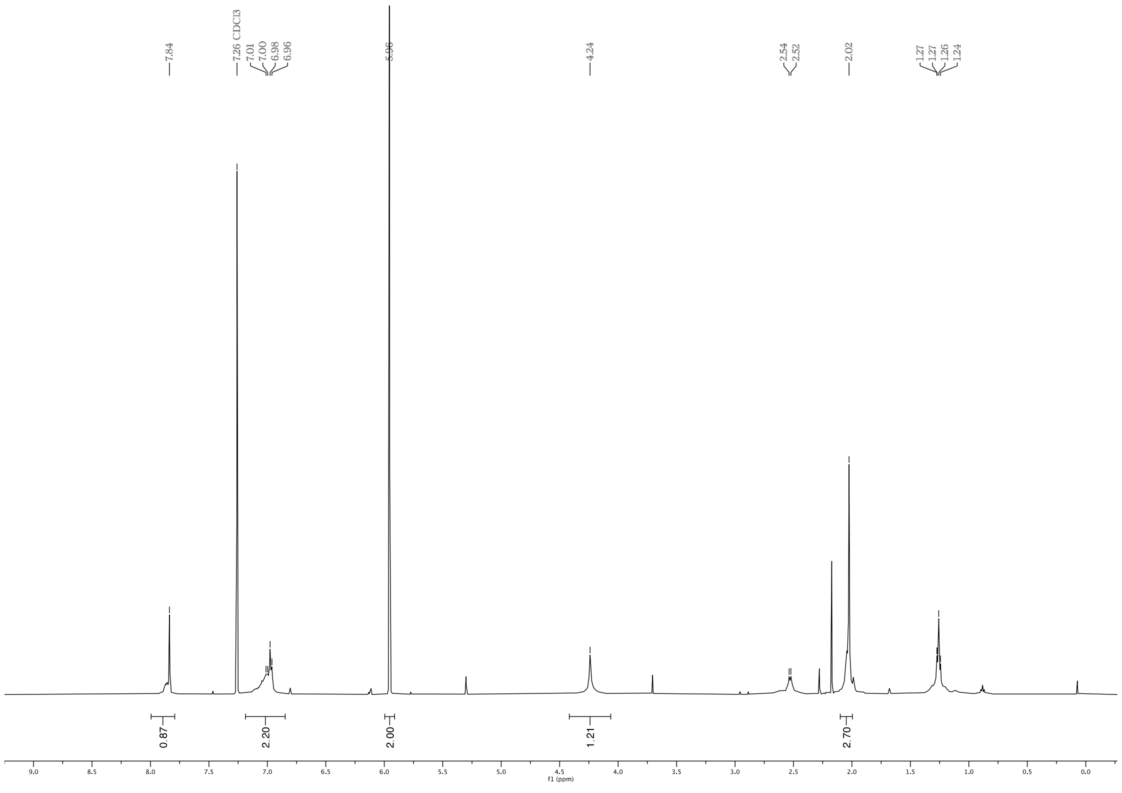

Fig. S39: Quantitative ^{1}H-NMR (500 MHz) spectrum of the self-assembly of **TP2** at 0.005 M with addition of 4 Å MS and using TCE as the IS. Only the signals used for quantification and the signal corresponding to the IS (TCE) and H_2O were integrated.

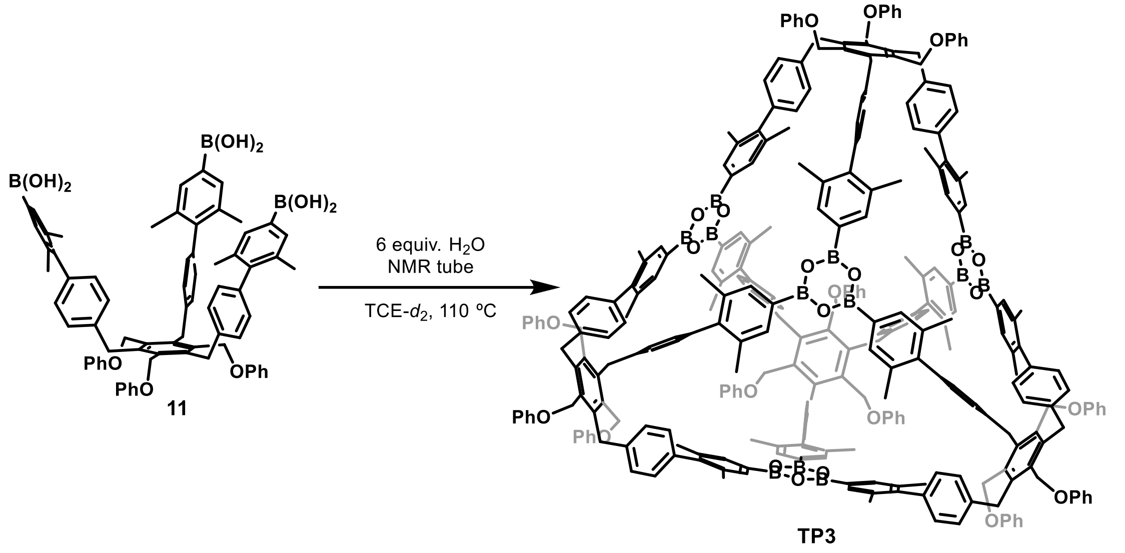

Boroxine Cage TP3. A NMR tube was charged with triboronic acid **11** (4.7 mg, 4.23 µmol, 1.0 equiv), H_2O (0.5 µL, 25 µmol, 6 equiv) and 20 µL of IS (0.1 M of 1,3,5-tribromobenzene in TCE-d_2) and anhydrous TCE-d_2 (0.75 mL, 0.005 M). Reaction control after 18 hours showed the presence of multiple species. After 36 hours no substantial change was observed and high temperature ^{1}H-NMR studies were performed to rule out a conformational equilibrium. Spectra were recorded at 100 °C, 120 °C and 140 °C, where the probe was left to equilibrate at the corresponding temperature for 10 minutes. Since the experiment indicated the existence of two distinct assemblies, an excess of water (2.5 µL, 140 µmol, 130 equiv) was added, the tube was shaken vigorously and heated to 80 °C for 72 hours. Clean formation of **TP3** was not observed.
Subsequently, 1 spatula tip of 4 Å molecular sieves was added and the reaction was heated to 80 °C for 18 hours. Again, convergence to **TP3** was not observed.
Finally, the tube was heated to 110 °C for 72 hours, however without obtaining the target compound exclusively.
Additionally, synthesis of **TP3** was explored using $CDCl_3$ (sealed pressure tube at 110 ºC), THF-d_8 (without and with 4 Å MS at 50 ºC). However, convergence toward cage **TP3** as the only species was not observed.

HRMS (MALDI+, DCTB, AgTFA) *m/z* calcd. for $C_{288}H_{252}{}^{11}B_{12}O_{24}{}^{109}Ag$ [M+Ag]$^+$ 4334.8867, found 4334.8843.

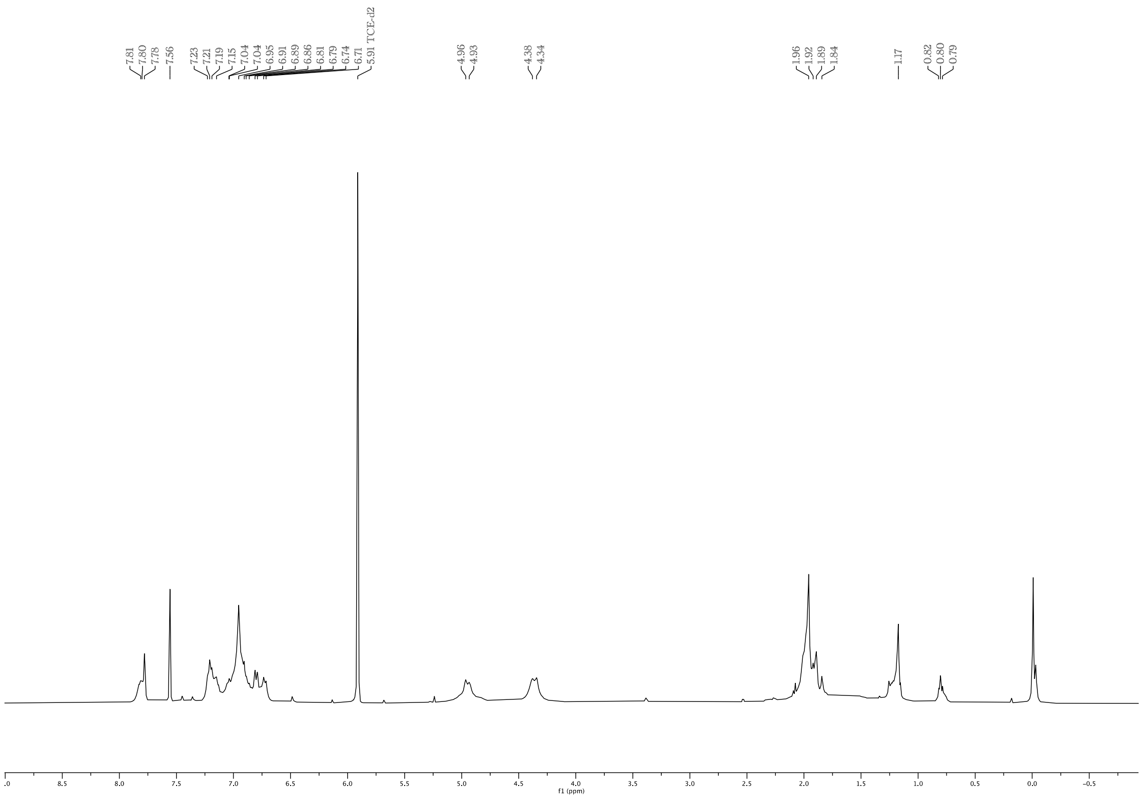

Fig. S40: ^{1}H-NMR (400 MHz) spectrum for the formation of cage **TP3** at 25 °C in TCE-d_2 after 18 hours.

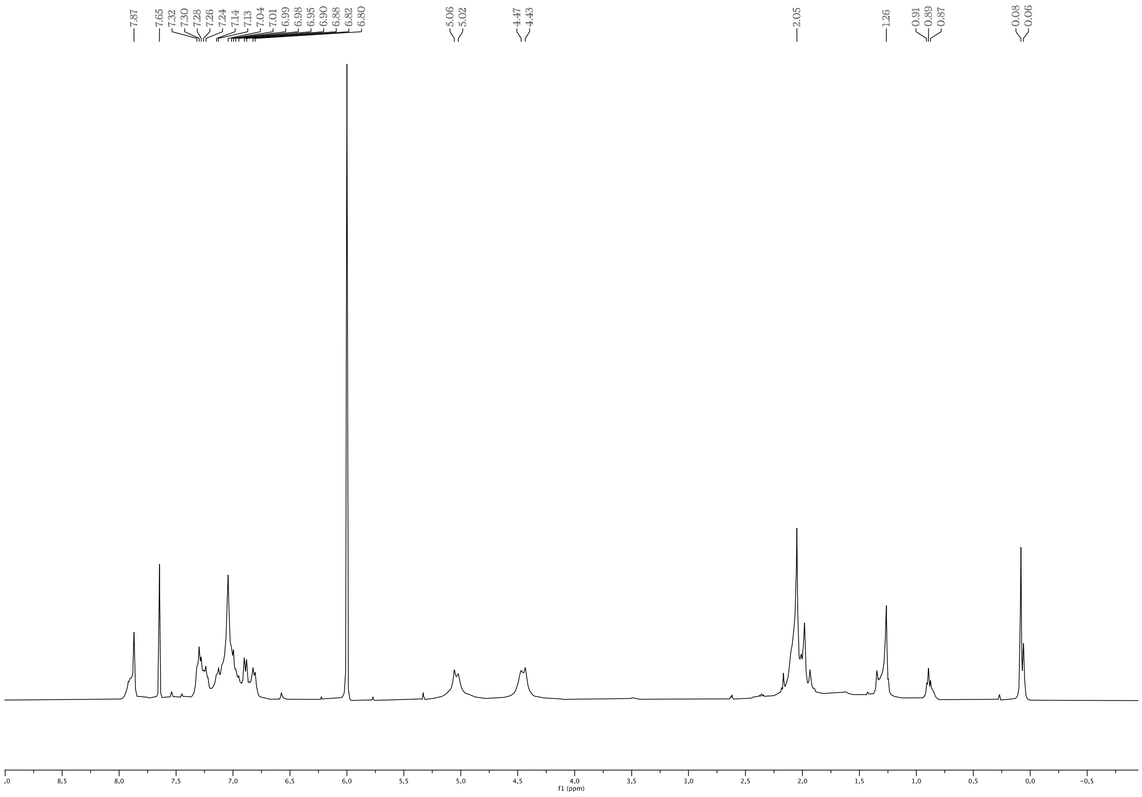

Fig. S41: ^{1}H-NMR (400 MHz) spectrum for the formation of cage **TP3** at 25 °C in TCE-d_2 after 36 hours.

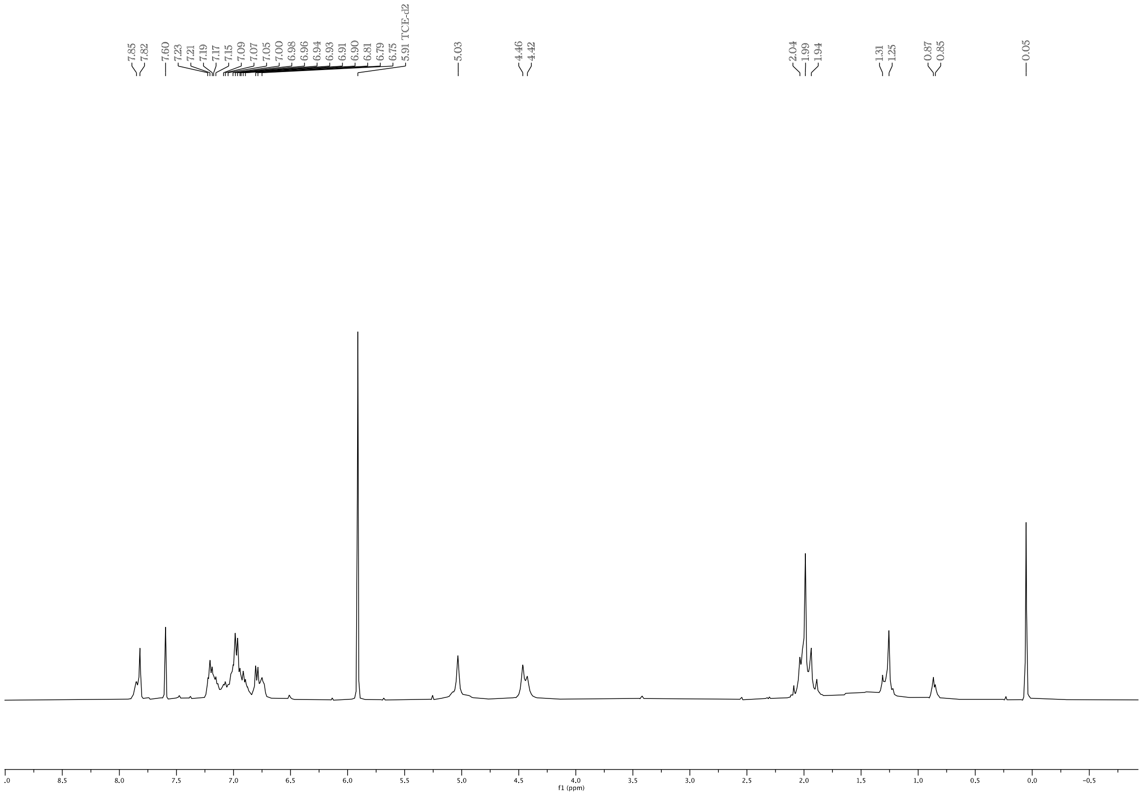

Fig. S42: ^{1}H-NMR (400 MHz) spectrum for the formation of cage **TP3** at 100 °C in TCE-d_2 after 36 hours.

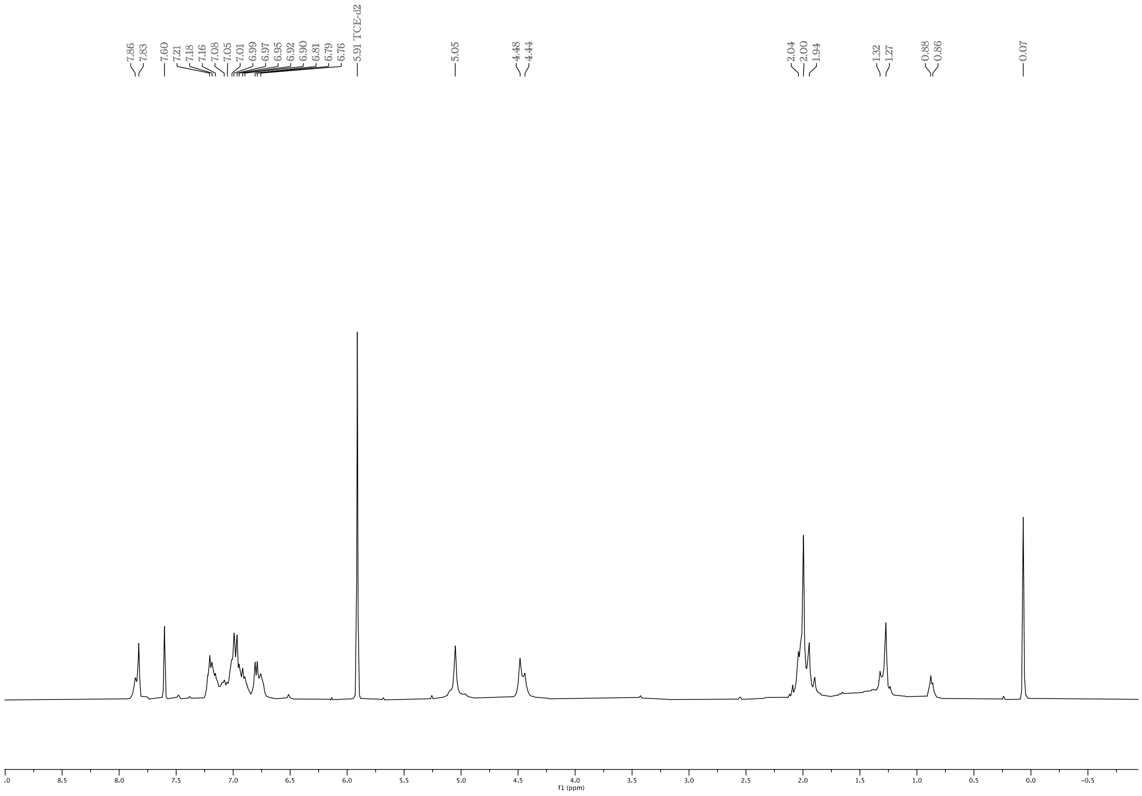

Fig. S43: ^{1}H-NMR (400 MHz) spectrum for the formation of cage **TP3** at 120 °C in TCE-d_2 after 36 hours.

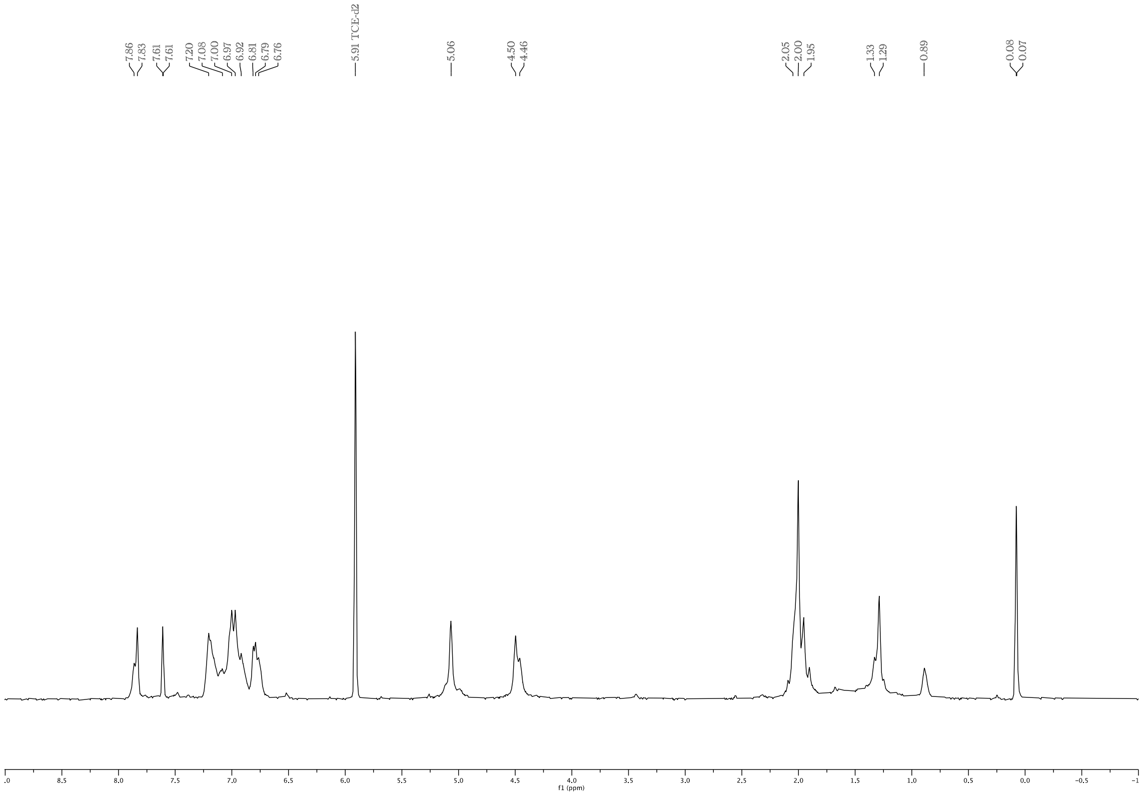

Fig. S44: ^{1}H-NMR (400 MHz) spectrum for the formation of cage **TP3** at 140 °C in TCE-d_2 after 36 hours.

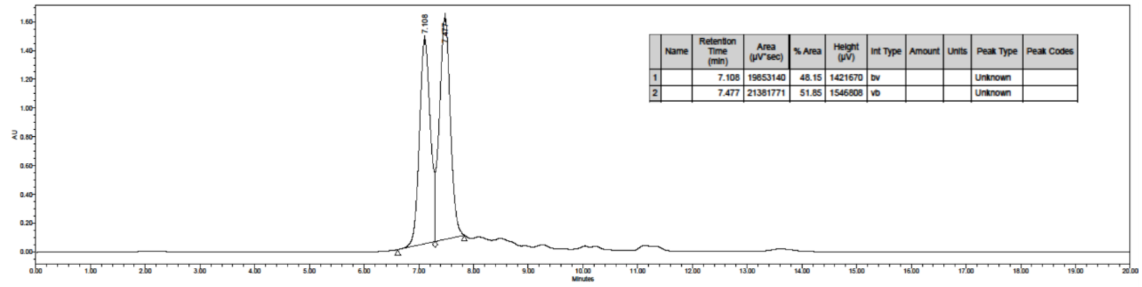

	Name	Retention Time (min)	Area (µV*sec)	% Area	Height (µV)	Int Type	Amount	Units	Peak Type	Peak Codes
1		7.108	19853140	48.15	1421670	bv			Unknown	
2		7.477	21381771	51.85	1546808	vb			Unknown	

Fig. S45: GPC traces for the formation of cage **TP3** after 1 week (Phenogel^{TM} 5µm 500 Å, 300 x 7.8 mm, CH_2Cl_2 as solvent at 1 mL/min).

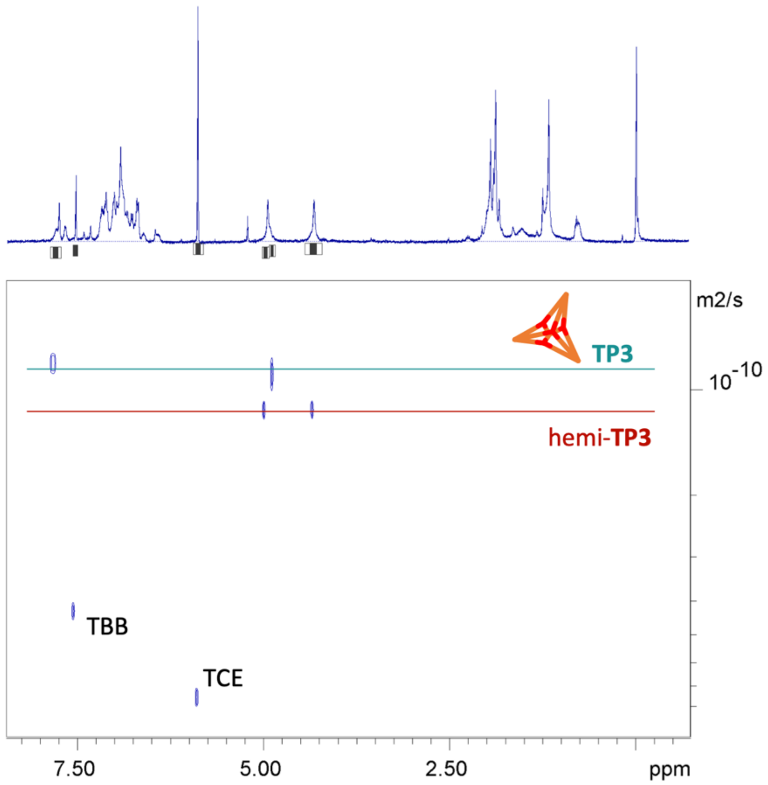

Fig. S46: ^{1}H-DOSY NMR spectrum for the formation of cage **TP3** (500 MHz, TCE-d_2).

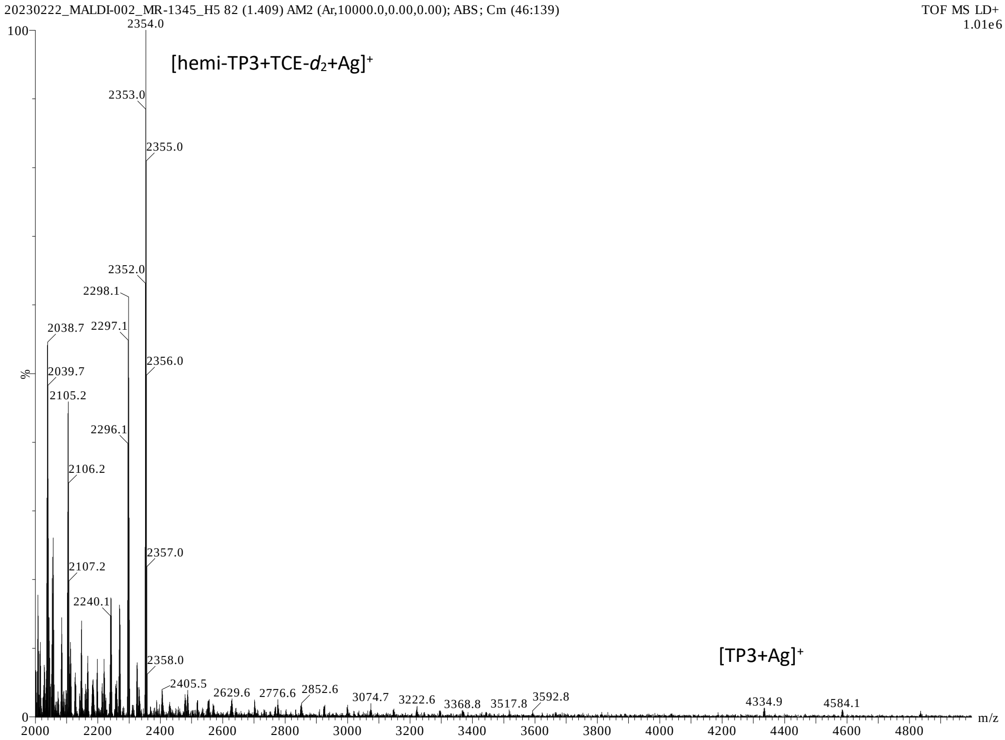

Fig. S47: HR-MALDI-TOF-MS spectrum of boroxine cage **TP3**.

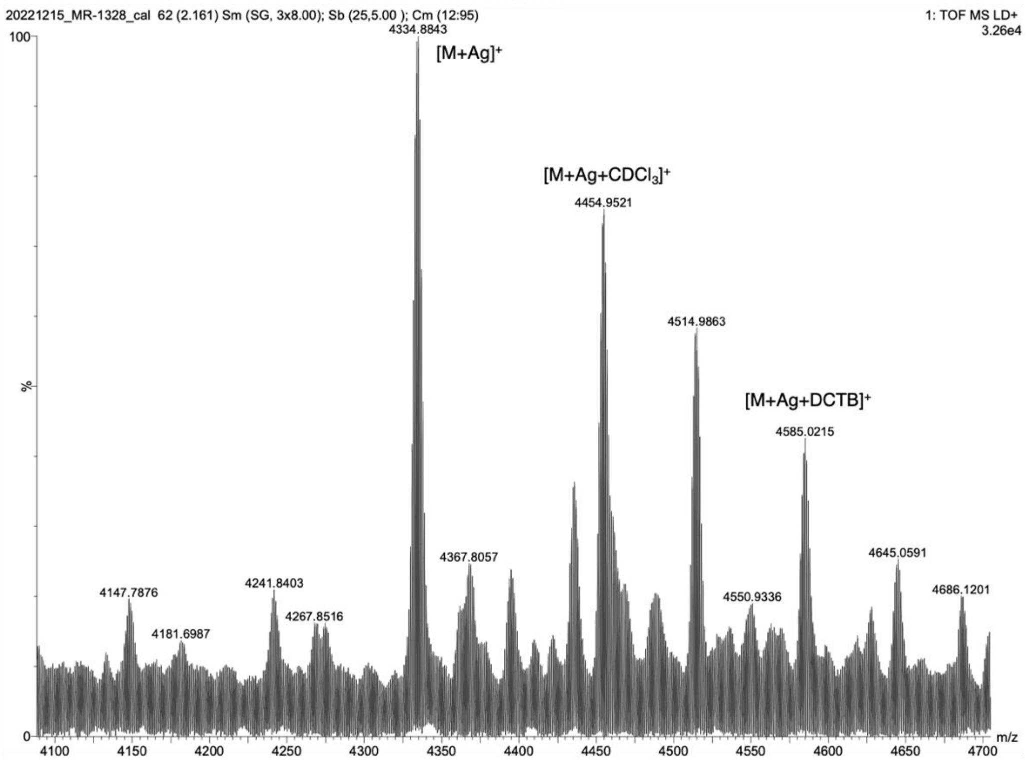

Fig. S48: HR-MALDI-TOF-MS spectrum of boroxine cage **TP3**.

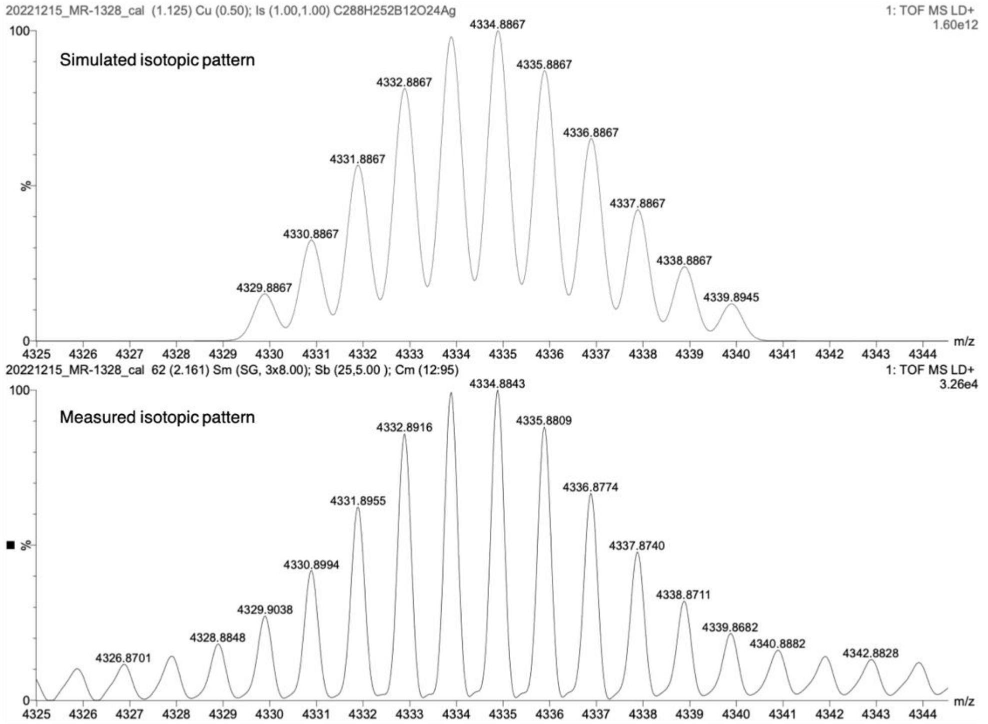

Fig. S49: Simulated (top) and measured (bottom) isotopic pattern for **TP3** with MALDI-TOF-MS.

5. Variable temperature NMR Studies of cage TP2.

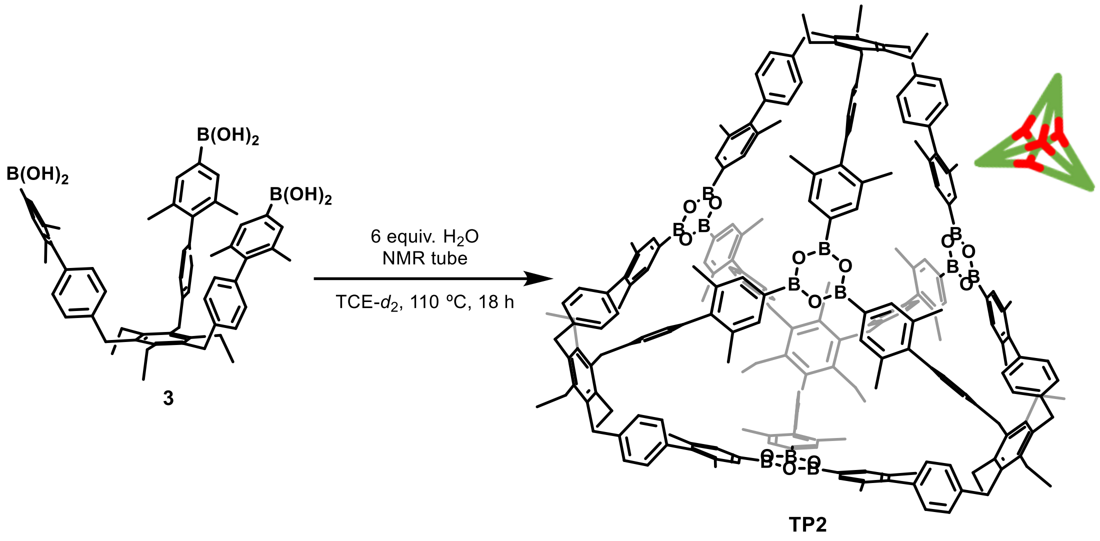

A NMR tube was charged with triboronic acid **3** (3.3 mg, 3.76 µmol, 1.0 equiv), H_2O (0.4 µL, 22.59 µmol, 6 equiv), and anhydrous TCE-*d*$_2$ (0.7 mL, 0.005 M). The NMR tube was sealed with a Teflon cap and heated to T = 110 °C overnight. Once the formation of **TP2** was confirmed, ^{1}H-NMR spectra were recorded at 25 °C, 50 °C, and 100 °C.

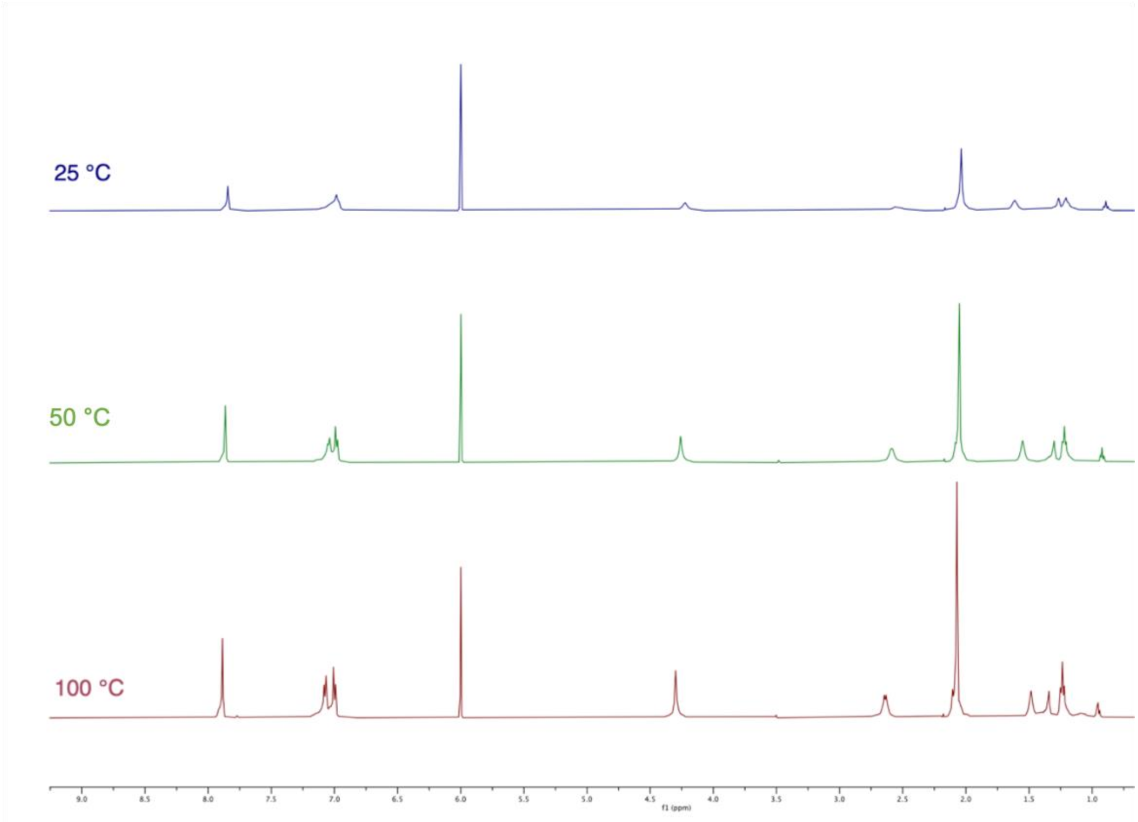

Fig. S50: Variable-temperature ^{1}H-NMR (500 MHz) studies of cage **TP2** in TCE-d$_2$.

Additionally, a ^{13}C-NMR and DEPT90 and DEPT135 spectra were recorded at 100 °C.

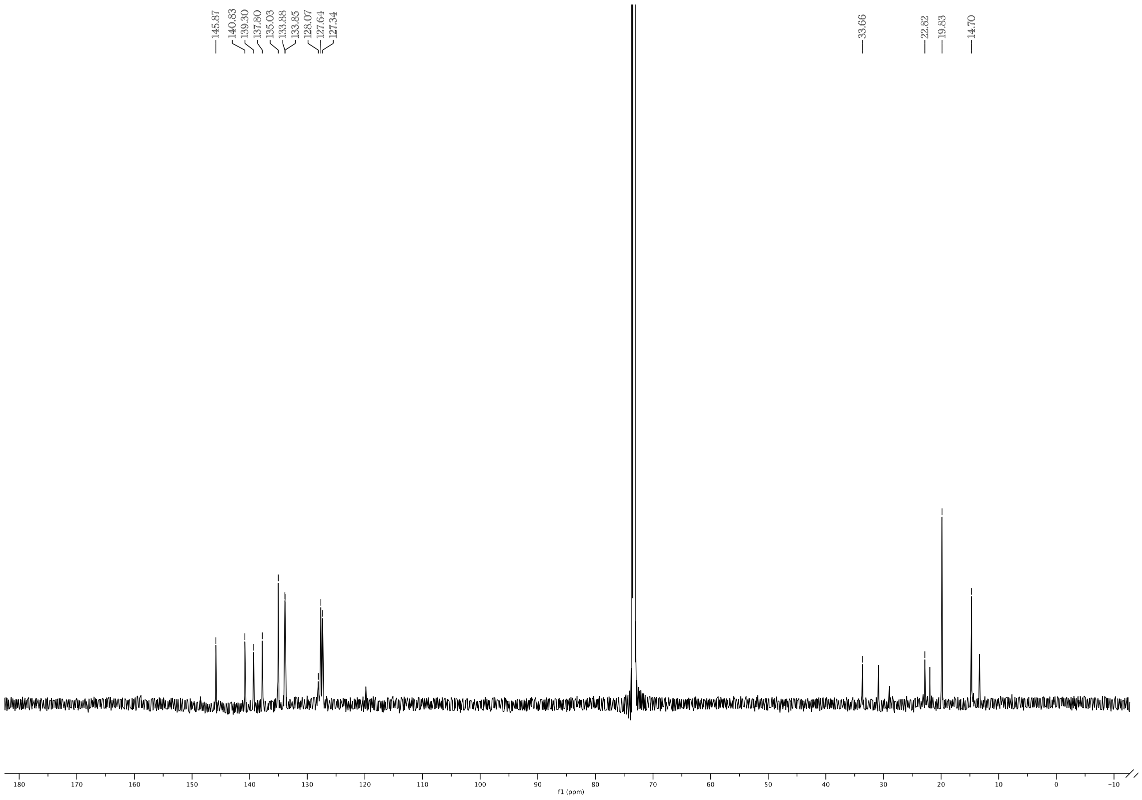

Fig. S51: ^{13}C-NMR (126 MHz) spectrum of cage **TP2** in TCE-d_2 at 100 °C.

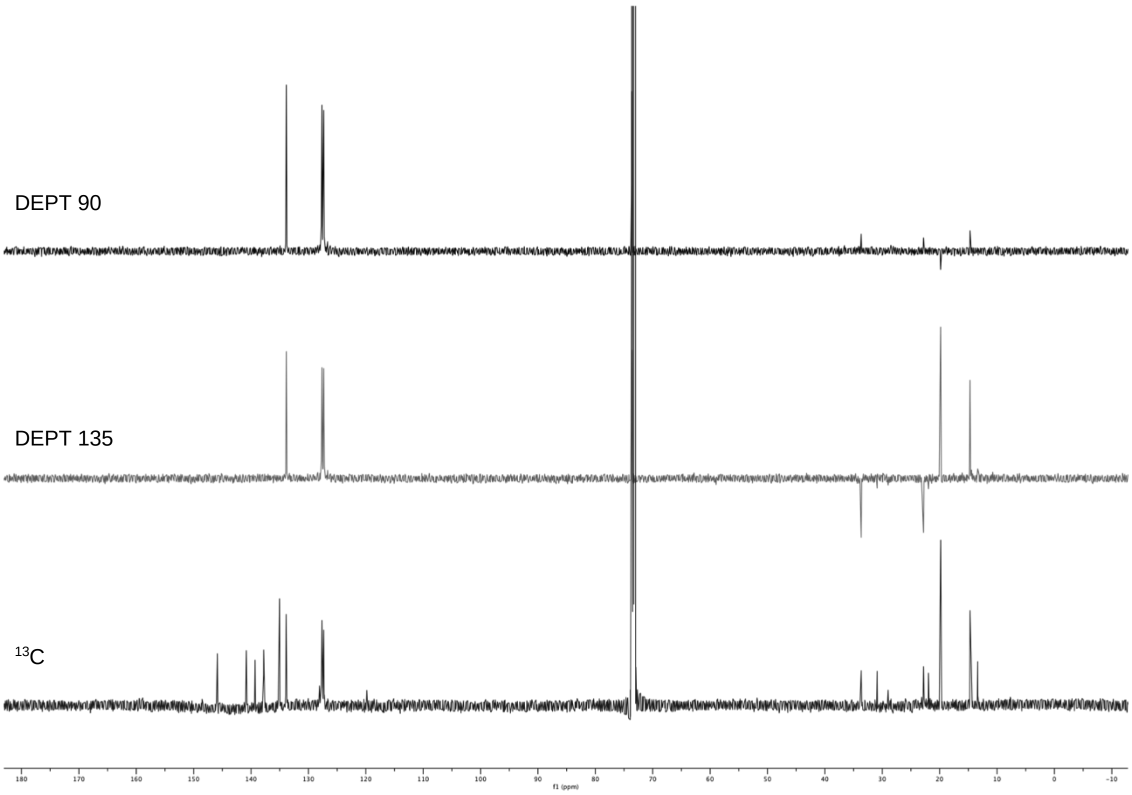

Fig. S52: ^{13}C-NMR (126 MHz) spectrum, DEPT90 and DEPT135 of cage **TP2** in TCE-d_2 at 100 °C.

Variable-temperature yield determination was carried out by adding 20 µL of IS (0.1 M of 1,3,5-tribromobenzene (TBB) in TCE-d_2) to the NMR tube, and heating the mixture to 100 °C overnight. Upon cooling the reaction mixture to RT, a quantitative ^{1}H-NMR spectrum was recorded at 25 °C. Subsequently, the probe was heated to 50 °C and maintained at this temperature for 30 minutes and a quantitative ^{1}H-NMR spectrum was recorded. Then, this procedure was repeated at 100 °C.

The semi-q spectra were recorded using calibrated 90 degrees pulses and relaxation delays of 15 s with a total of 32 scans. Four integration regions of product signals were chosen (8.00-7.75, 7.25-6.80, 4.45-3.95, 2.15-1.93 ppm), maintained for the different temperatures and integrated in relation to the 1,3,5-tribromobenzene signal (the integral of the Ar-*H* signal was set to a value of one). For each of the four product signals, the yield (y) was determined, and the average of all signals gave the yield (Y) of the reaction. The error ΔY was calculated using the standard deviation function of the four values of y.

Table S2: Variable-temperature yield determination for cage **TP2** based on quantitative ^{1}H-NMR measurements using 1,3,5-tribromobenzene as the internal standard.

	T [°C]	Signal [ppm]	Integral (I)	Nuclei (N)	I/N	n_{cage}[1] [mmol]	$n_{100\%}$[2] [mmol]	y [%]	ΔY [%]
**TP2**	**25**	8.0-7.75	2.12	24	0.08833	0.000549	0.00094	58.4	
		7.25-6.80	4.94	48	0.10292	0.000640	0.00094	68.1	
		4.45-3.95	2.42	24	0.10083	0.000627	0.00094	66.7	
		2.15-1.93	6.72	72	0.09333	0.000580	0.00094	61.7	
							**$Y_{25\,°C}$:**	**63.7**	**4**
**TP2**	**50**	8.0-7.75	2.17	24	0.09042	0.000562	0.00094	59.8	
		7.25-6.80	5.24	48	0.10917	0.000679	0.00094	72.2	
		4.45-3.95	2.54	24	0.10583	0.000658	0.00094	70.0	
		2.15-1.93	7.16	72	0.09944	0.000618	0.00094	65.8	
							**$Y_{50\,°C}$:**	**66.9**	**5**
**TP2**	**100**	8.0-7.75	2.88	24	0.12	0.000746	0.00094	79.4	
		7.25-6.80	6.81	48	0.14188	0.000882	0.00094	93.8	
		4.45-3.95	3.37	24	0.14042	0.000873	0.00094	92.9	
		2.15-1.93	9.61	72	0.13347	0.000830	0.00094	88.3	
							**$Y_{100\,°C}$:**	**88.6**	**7**

[1] n_{cage} is calculated by multiplying the moles of added TBB by the I/N ration and defines the moles of boroxine cage **TP2** in the NMR tube. [2] $n_{100\%}$ is the moles of cage for a theoretical yield of 100%.

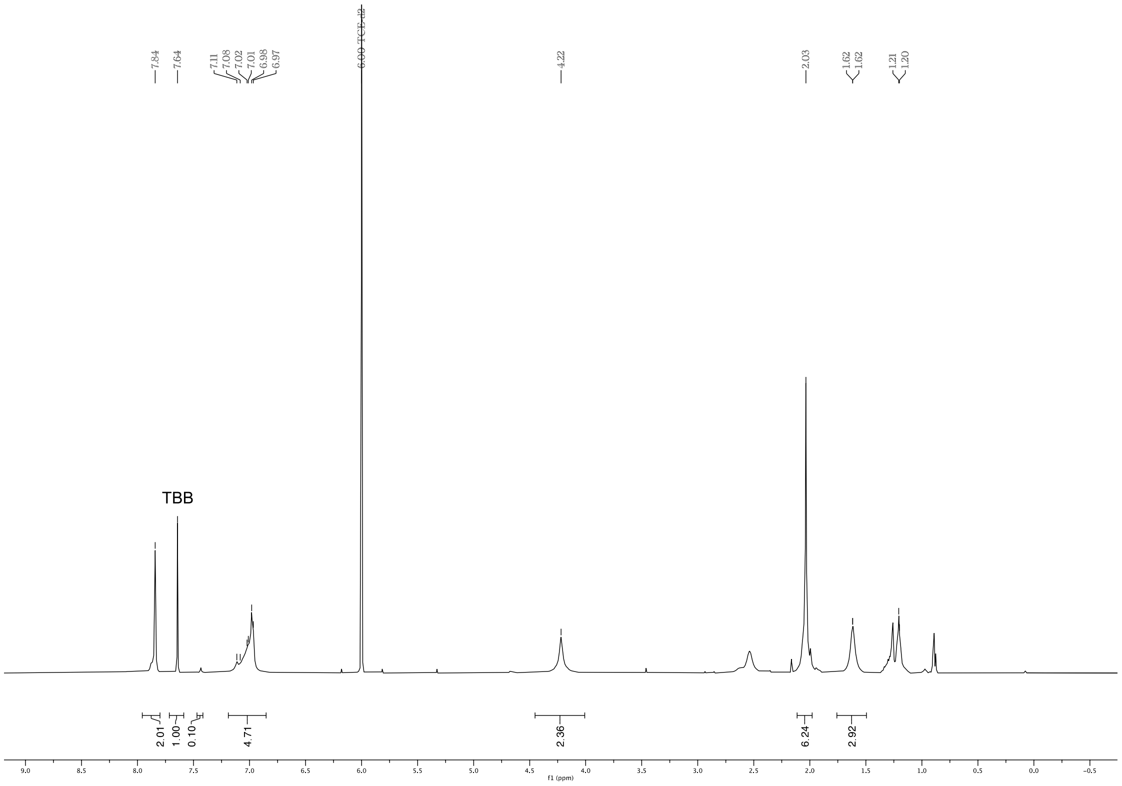

Fig. S53: Quantitative ^{1}H-NMR (500 MHz) spectrum of the self-assembly of cage **TP2** in TCE-d_2, using 1,3,5-tribromobenzene as the IS recorded at 25 °C. Only the signals used for quantification and the signal corresponding to the IS (TBB) and H_2O were integrated.

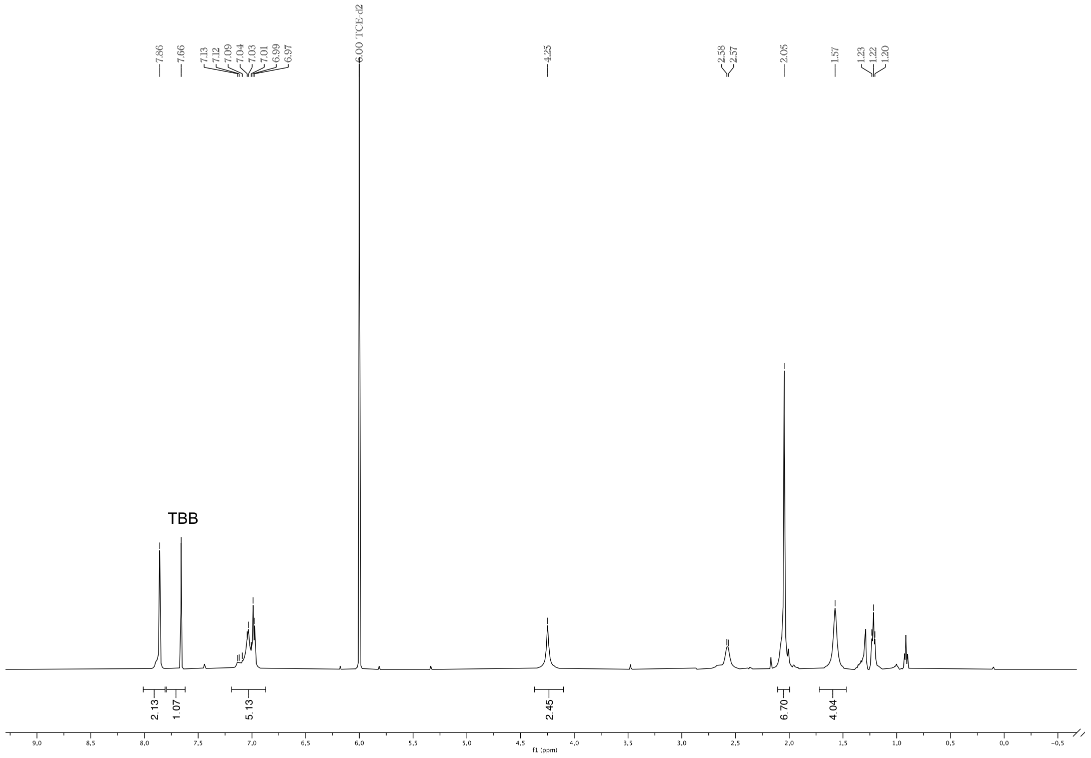

Fig. S54: Quantitative ^{1}H-NMR (500 MHz) spectrum of the self-assembly of cage **TP2** in TCE-d_2, using 1,3,5-tribromobenzene as the IS recorded at 50 °C. Only the signals used for quantification and the signal corresponding to the IS (TBB) and H_2O were integrated.

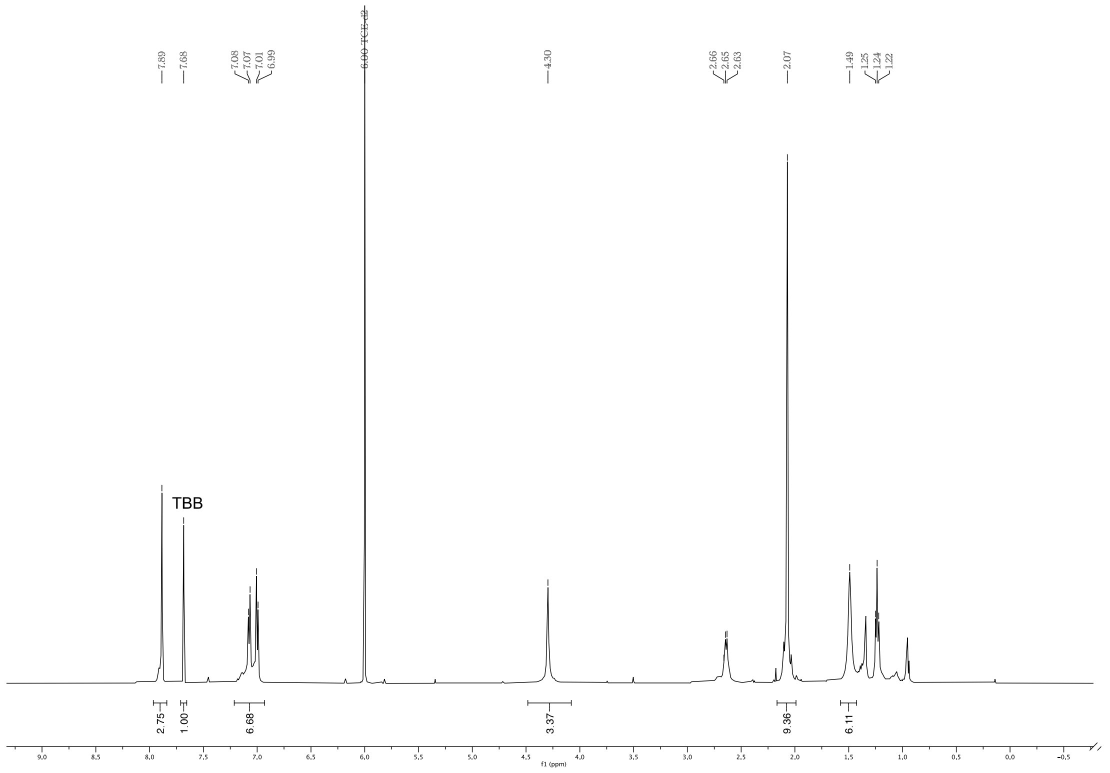

Fig. S55: Quantitative ^{1}H-NMR (500 MHz) spectrum of the self-assembly of cage **TP2** in TCE-d_2, using 1,3,5-tribromobenzene as the IS recorded at 100 °C. Only the signals used for quantification and the signal corresponding to the IS (TBB) and H_2O were integrated.

Yield determination of TP2 in TCE-d₂ at 100 °C after the addition of molecular sieves.

The general procedure for the formation of boroxine cages was applied to triboronic acid precursor **3** (12 mg, 13.6 µmol, 1.0 equiv) using 2.7 mL TCE-d_2 (0.005 M) with H_2O (1.5 µL, 81.6 µmol, 6 equiv). After one night, the reaction was cooled to room temperature, and the formation of a very fine white precipitate was observed (this precipitate was previously confirmed to be tripod **3** by HRMS-MALDI-TOF, manuscript reference 20). A 250 µL aliquot was extracted and placed in an NMR tube to which 250 µL of TCE-d_2 and 20 µL of a solution of TBB in TCE-d_2 (0.095 M) were added. Then, a quantitative ^{1}H-NMR spectrum was recorded at 25 °C and the **TP2** yield (Y = 70 ± 5 %) was determined using TBB as the internal standard. The reaction was heated again at 100 °C for 30 minutes, and at this temperature, several pellets of 4 Å, activated molecular sieves were added. The reaction was left for 20 minutes at that temperature and then cooled to room temperature. This time, the formation of the white precipitate was not observed. A 250 µL aliquot was extracted and placed in an NMR tube to which 250 µL of TCE-d_2 and 20 µL of a solution of TBB in TCE-d_2 (0.095 M) were added. Then, a quantitative ^{1}H-NMR spectrum was recorded at 25 °C and the **TP2** yield (Y = 92 ± 7 %, with a 98% purity according to the GPC trace) was determined using TBB as the internal standard.

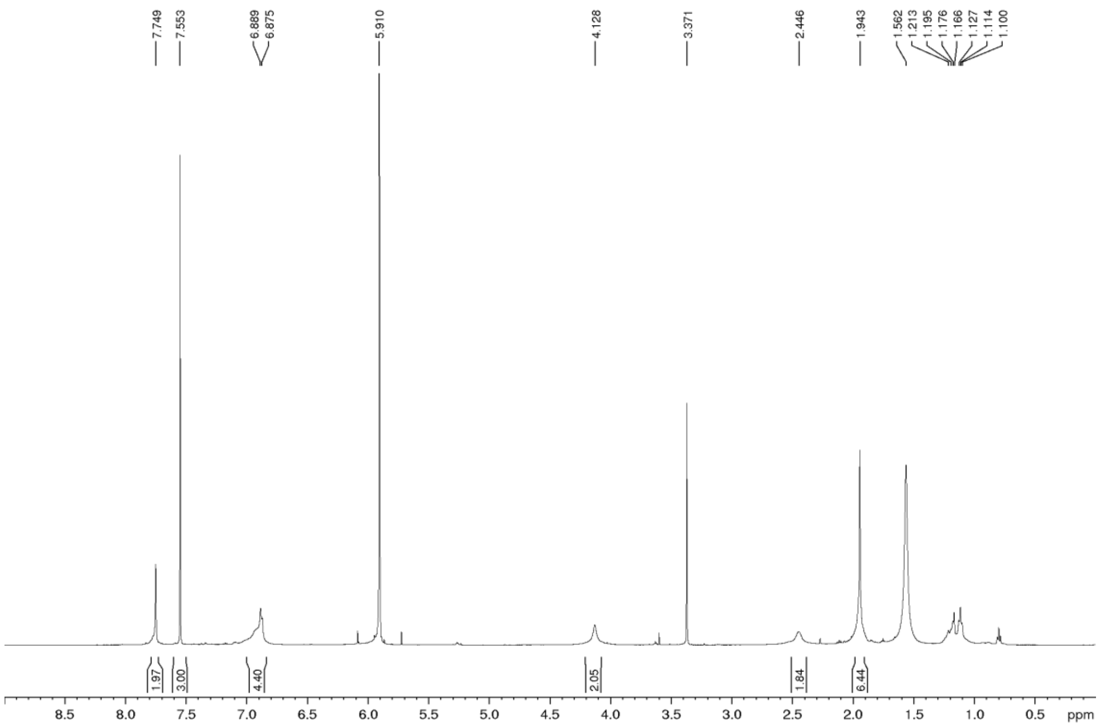

Fig. S56: Quantitative ^{1}H-NMR (500 MHz) spectrum of the self-assembly of cage **TP2** in TCE-d_2 (before addition of molecular sieves), using 1,3,5-tribromobenzene as the IS recorded at RT. Only the signals used for quantification and the signal corresponding to the IS (TBB).

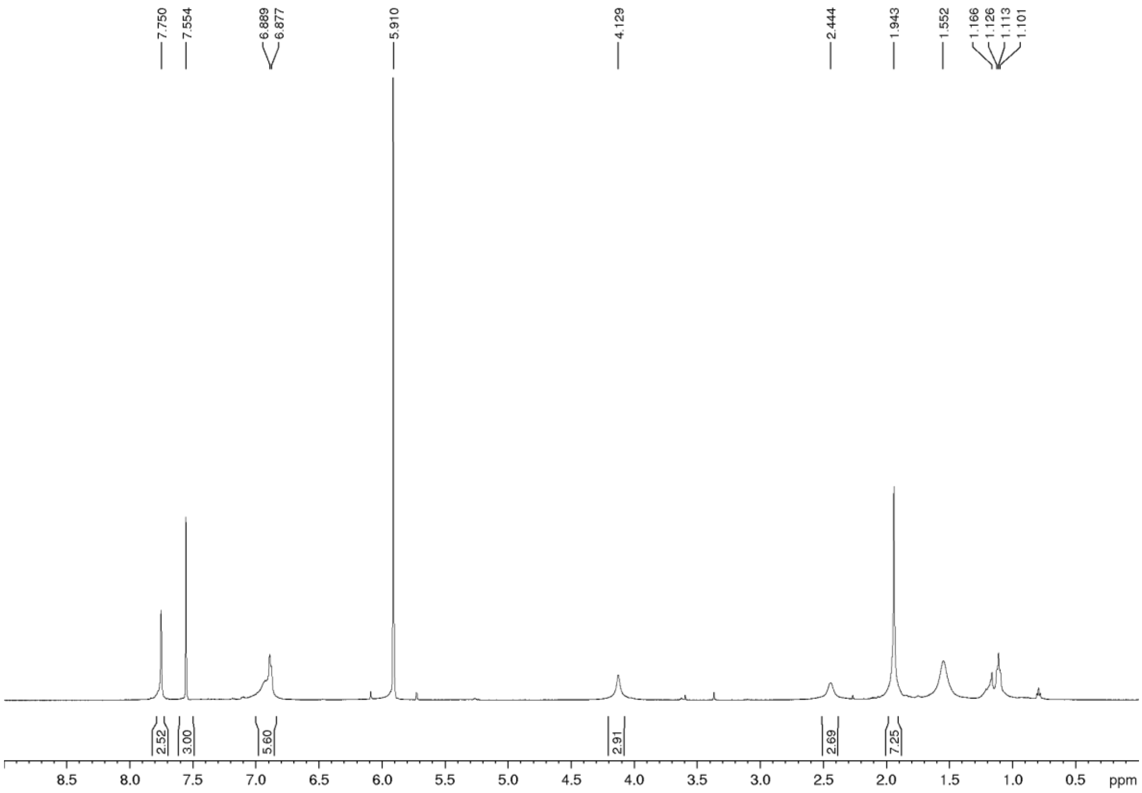

Fig. S57: Quantitative ^{1}H-NMR (500 MHz) spectrum of the self-assembly of cage **TP2** in TCE-d_2 (after the addition of molecular sieves at 100 ºC), using 1,3,5-tribromobenzene as the IS recorded at RT. Only the signals used for quantification and the signal corresponding to the IS (TBB).

Table S3: Yield determination for cage **TP2** based on quantitative ^{1}H-NMR measurements using 1,3,5-tribromobenzene as the internal standard.

	**T [°C]**	**Signal [ppm]**	**Integral (I)**	**Nuclei (N)**	**I/N**	**n_{cage}[1] [mmol]**	**n_{total}[2] [mmol]**	**y [%]**	**ΔY [%]**
**TP2**	**25**	7.75	1.97	24	0.08208333	0.000156449	0.00168964	67.6	
		7.2	4.4	48	0.09166667	0.000174714	0.00188691	75.5	
		4.2	2.05	24	0.08541667	0.000162802	0.00175826	70.3	
		2.5	1.84	24	0.07666667	0.000146125	0.00157814	63.1	
		2.02	6.44	72	0.08944444	0.000170479	0.00184117	73.6	
							**Y:**	**70.0**	**4.9**
**TP2**	**25**	7.75	2.52	24	0.105	0.000200127	0.00216137	86.5	
		7.2	5.6	48	0.11666667	0.000222363	0.00240152	96.1	
		4.2	2.91	24	0.12125	0.000231099	0.00249587	99.8	
		2.5	2.69	24	0.11208333	0.000213628	0.00230718	92.3	
		2.02	7.25	72	0.10069444	0.000191921	0.00207274	82.9	
							**Y:**	**91.5**	**6.9**

[1] n_{cage} is calculated by multiplying the moles of added TBB by the I/N ration and defines the moles of boroxine cage **TP2** in the NMR tube. [2] n_{total} is the moles of cage in the reaction.

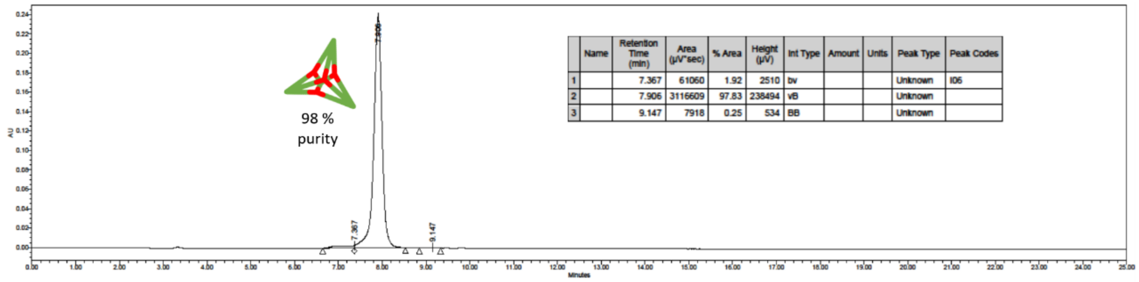

Fig. S58: GPC trace for the formation of cage **TP2** in TCE-d_2 (after the addition of molecular sieves at 100 ºC) (Phenogel™ 5µm 500 Å, 300 x 7.8 mm, CH_2Cl_2 as solvent at 1 mL/min).

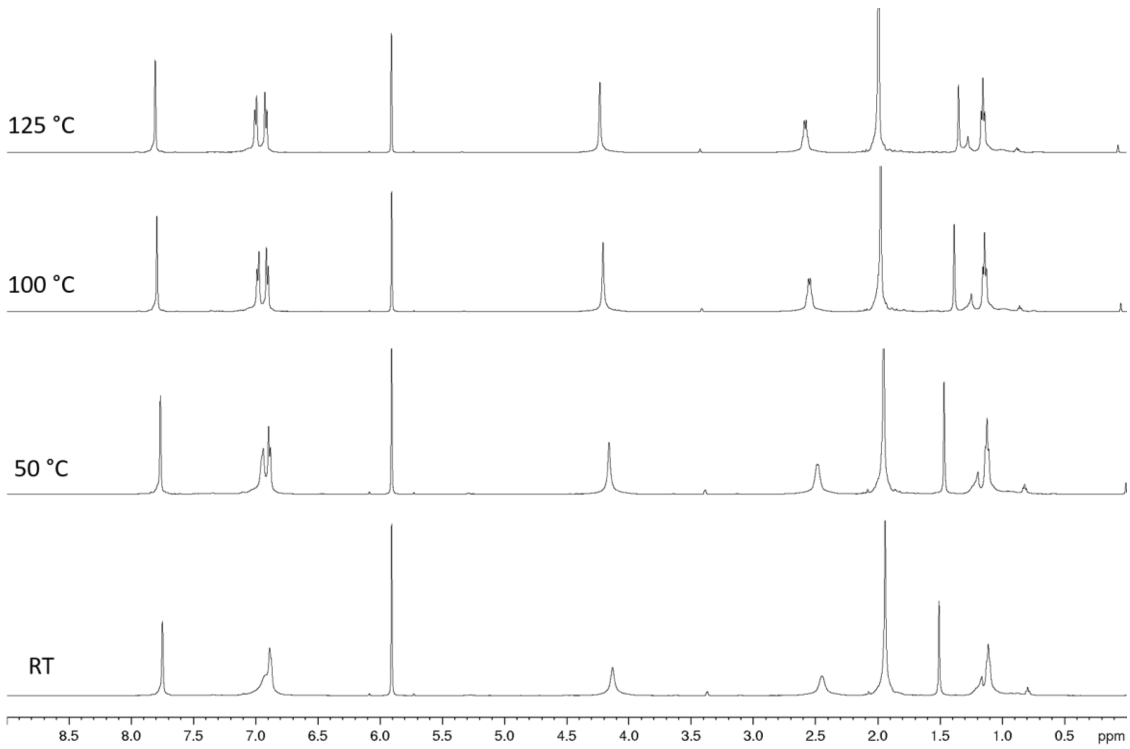

Fig. S59: VT ^{1}H-NMR of cage **TP2** in TCE-d_2 (after the addition of molecular sieves at 100 ºC).

6. Synthesis and Characterization of boronate Cages

General procedure for the synthesis boronate cages: A two-necked pressure tube equipped with a magnetic stirring bar was charged with triboronic acid (1.0 equiv), **THB** (1.5 equiv), H_2O (2.0 equiv per boronic acid moiety), and was evacuated and back-filled with N_2 for a total of five times. Subsequently, anhydrous $CDCl_3$ was added under positive N_2 pressure, and the tube was sealed tight, and heated to 110 °C. The initially heterogeneous mixture turned homogenous upon completion of the reaction. After allowing it to cool to RT, a 0.5 mL aliquot was extracted under positive N_2 pressure, which was analyzed by ^{1}H NMR spectroscopy. The yield was determined by adding 1,1,2,2-tetrachloroethane (TCE) as an internal standard and recording a quantitative proton NMR spectrum.

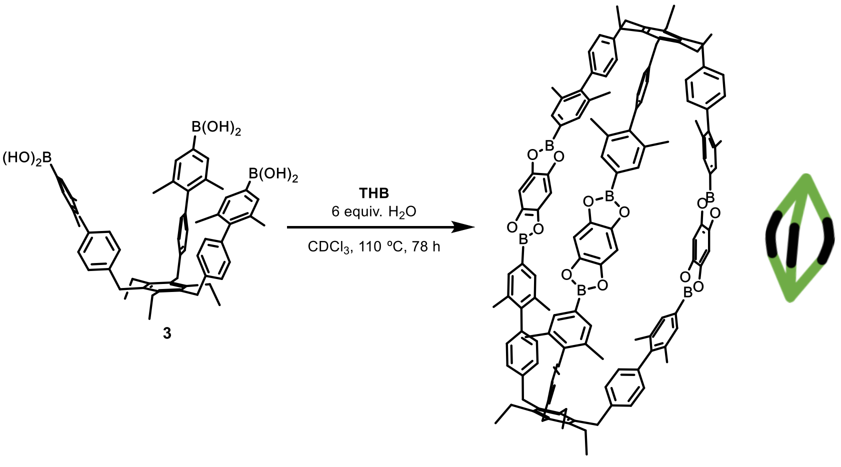

BP1

Boronate cage BP1. General procedure for the formation of boronate cages was applied to triboronic acid precursor **3** (8.3 mg, 9.47 µmol, 1.0 equiv), benzene-1,2,4,5-tetraol (**THB**) (2.02 mg, 14.2 µmol, 1.5 equiv) using 1.9 mL anhydrous $CDCl_3$ (0.005 M) with H_2O (1.0 µL, 56.8 µmol, 6.0 equiv). The product formed after three days with a yield of 39 ± 0 %. **BP1** was purified by size-exclusion chromatography using an Ultrastyragel® 500 Å (19x300mm) column on a Waters 600 E with UV detection at 254 nm and CH_2Cl_2 as solvent at 1mL/min. The clean fractions were joined, 0.75 mL TCE-d_2 were added and the CH_2Cl_2 was removed by rotatory evaporation at RT over several hours to give pure **BP1**.

^{1}H NMR (500 MHz, TCE-d_2) δ 7.64 (s, 24H, *H*-10), 7.18 (s, 6H, *H*-11), 6.88 (d, *J* = 7.4 Hz, 12H, *H*-5), 6.79 (d, *J* = 8.4 Hz, *H*-4), 4.12 (s, 12H, *H*-2), 2.47 (q, *J* = 6.5 Hz, 12H, *H*-2'), 1.87 (s, 36H, *H*-9), 1.13 (t, *J* = 7.2 Hz, 1H, *H*-3').

^{13}C NMR (126 MHz, TCE-d_2) δ 145.7, 143.0, 140.5, 139.2, 137.1, 135.6, 134.4, 133.1, 127.6, 127.4, 98.9, 97.7, 33.4, 29.2, 20.0, 15.1.

^{1}H DOSY-NMR (500 MHz, $CDCl_3$): $D = 4.01 \pm 0.02 \times 10^{-10}$ m^2/s

HRMS (MALDI+, DCTB, AgTFA) *m/z* calcd. for $C_{132}H_{120}{}^{10}B^{11}B_5O_{12}Ag$ $[M+Ag]^+$ 2070.8422, found 2070.8477.

IR (ATR, FT) ν (cm^{-1}) 727, 906, 1003, 1141, 1342, 1364, 1513, 2929, 2967, 3399.

UV/vis (Cyclohexane) λ_{max} (nm) 224, 252, 301, 312.

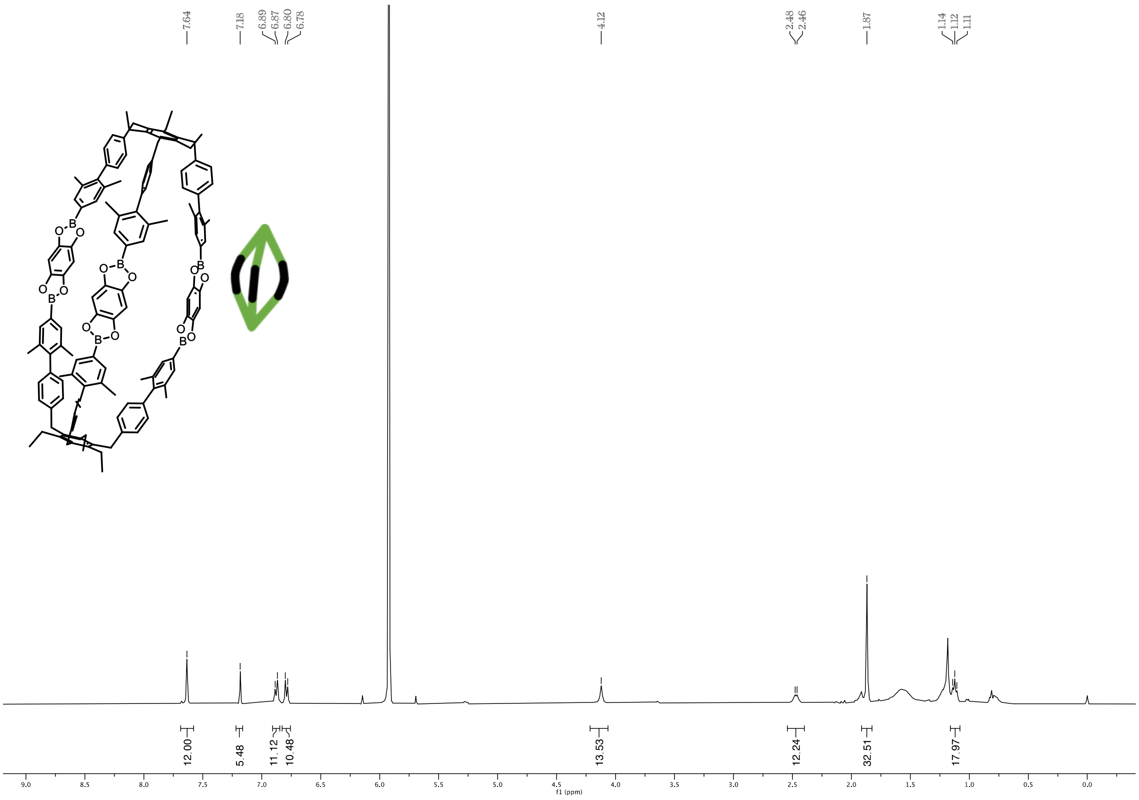

Fig. S60: ^{1}H-NMR (500 MHz, TCE-d_2, RT) spectrum of purified cage **BP1**.

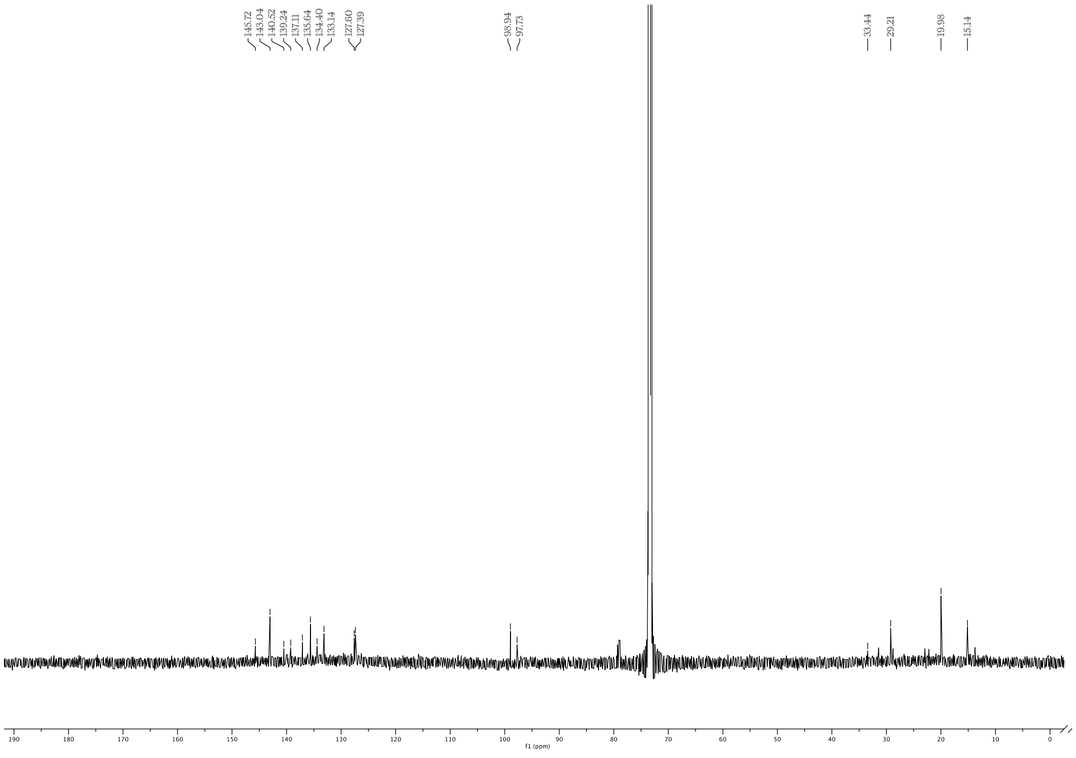

Fig. S61: ^{13}C-NMR (126 MHz, TCE-d_2, RT) spectrum of purified cage **BP1**.

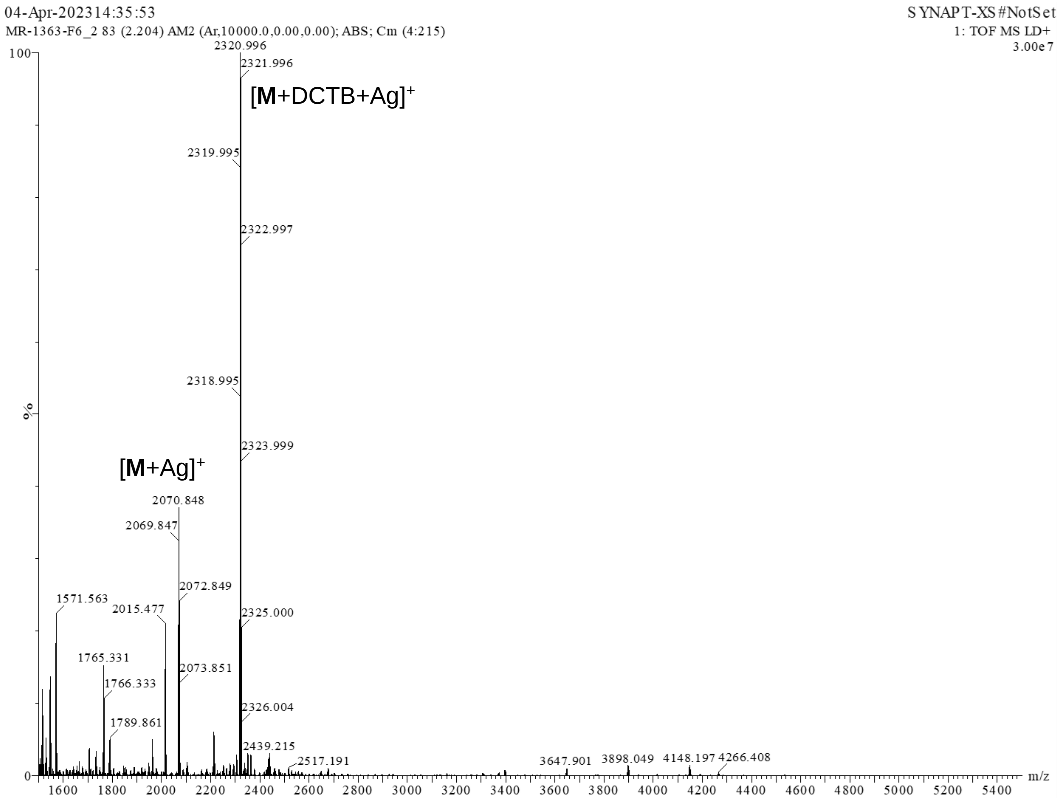

Fig. S62: Full MALDI-TOF-MS spectrum of purified cage **BP1**.

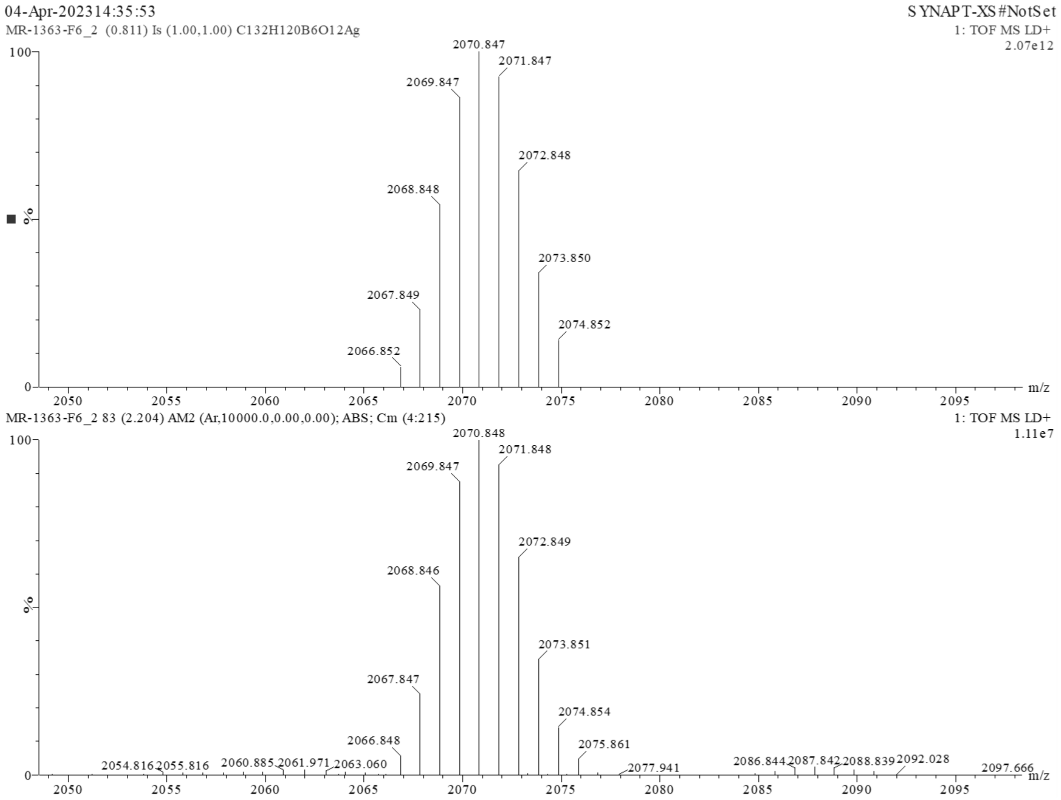

Fig. S63: Simulated (top) and measured (bottom) isotopic pattern for purified **BP1** with HR-MALDI-TOF-MS.

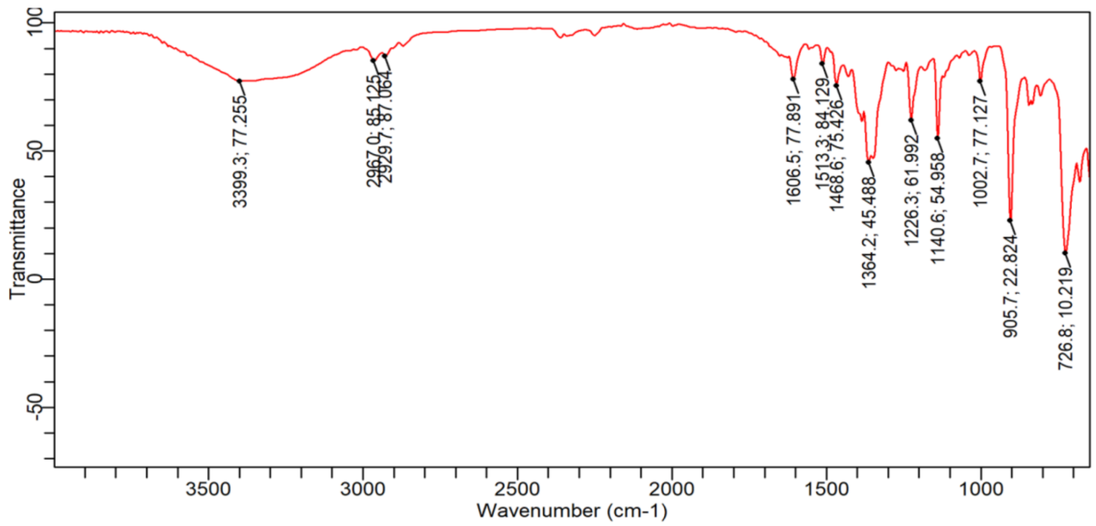

Fig. S64: FT-IR spectrum of **BP1** (ATR, diamond).

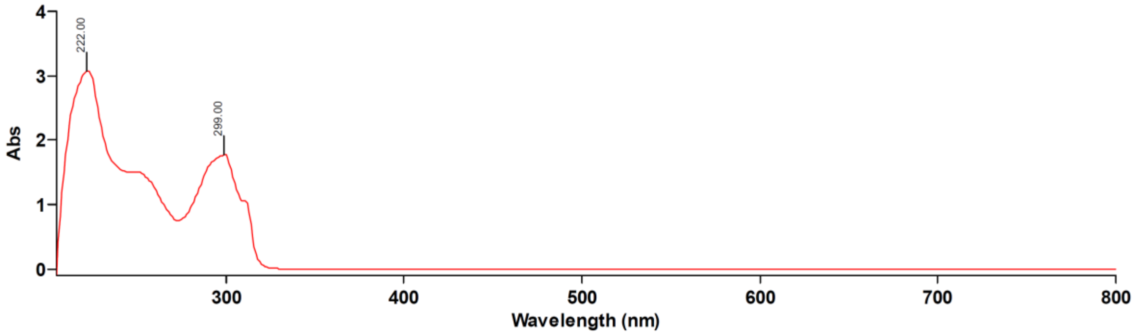

Fig. S65: UV/vis spectrum of **BP1** in cyclohexane.

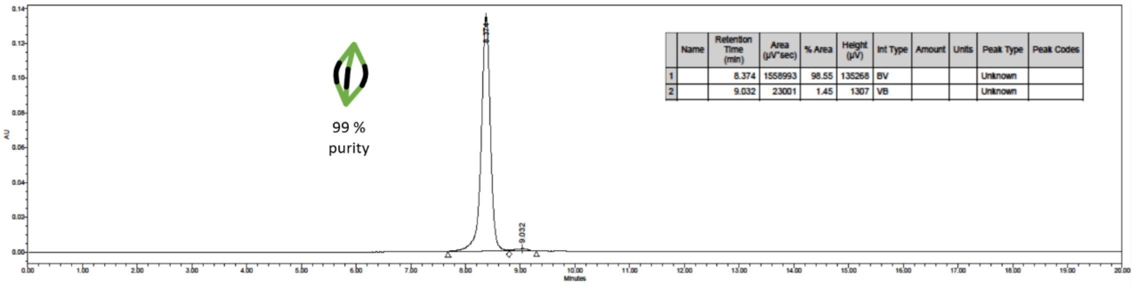

	Name	Retention Time (min)	Area (µV*sec)	% Area	Height (µV)	Int Type	Amount	Units	Peak Type	Peak Codes
1		8.374	1558993	98.55	135268	BV			Unknown	
2		9.032	23001	1.45	1307	VB			Unknown	

Fig. S66: GPC trace for purified cage **BP1** (Phenogel^{TM} 5µm 500 Å, 300 x 7.8 mm, CH_2Cl_2 as solvent at 1 mL/min).

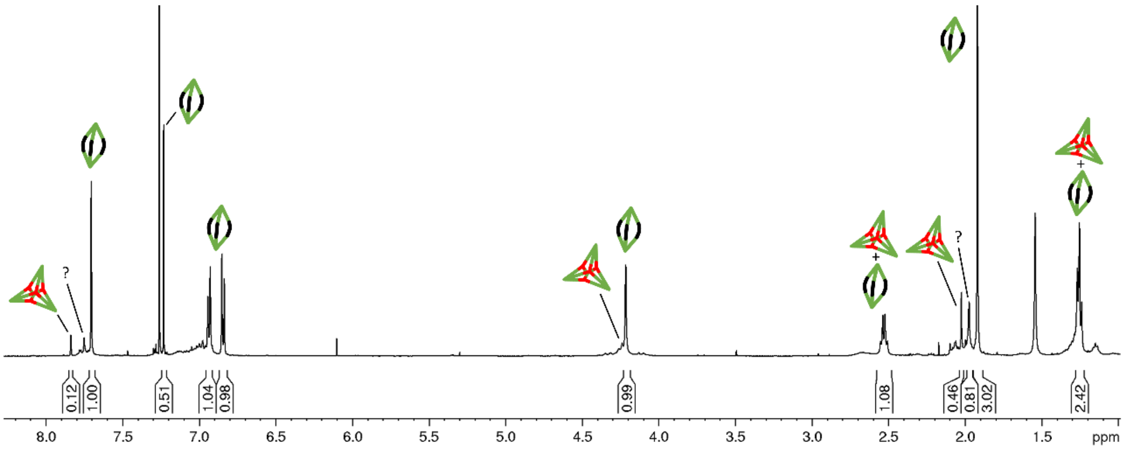

Fig. S67: ^{1}H-NMR (500 MHz, $CDCl_3$) spectrum of the assembly of cage **BP1** at 72 hours, recorded at 25 °C. The different species (**BP1**, **TP2** and unknown) are assigned using cartoon representation.

Table S4: Yield determination for cage formation **BP1** based on quantitative ^{1}H-NMR measurements using TCE as the internal standard after 72 hours in $CDCl_3$ at 110 ºC in a sealed tube.

cage	Signal [ppm]	Integral (I)	Nuclei (N)	I/N	$n_{aliquot}$[1] [mmol]	n_{tot}[2] [mmol]	$n_{100\%}$[3] [mmol]	y [%]	ΔY [%]
**BP1**	7.7	0.76	12	0.06333	0.0005999	0.0018598	0.0047400	39	
	7.2	0.37	6	0.06167	0.0005842	0.0018109	0.0047400	38	
	6.9	0.76	12	0.06333	0.0005999	0.0018598	0.0047400	39	
	4.2	0.75	12	0.06250	0.0005921	0.0018354	0.0047400	39	
	1.9	2.28	36	0.06333	0.0005999	0.0018598	0.0047400	39	
							**Y**	**39**	**0**
**TP2**	7.9	0.09	24	0.00375	0.0000355	0.0001101	0.0023700	5	
	2.0	0.33	72	0.00458	0.0000434	0.0001346	0.0023700	6	
							**Y**	**5**	**1**
**?**	7.8	0.15	24	0.00625	0.0000592	0.0001835	0.0023700	8	
	2.0	0.59	72	0.00819	0.0000434	0.0001345	0.0023700	6	
							**Y**	**7**	**1**

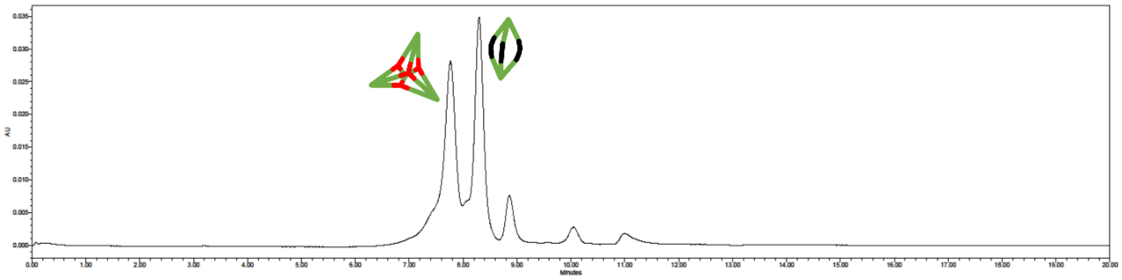

Fig. S68: GPC trace of the assembly of cage **BP1** at t = 24 hours (Phenogel™ 5µm 500 Å, 300 x 7.8 mm, CH_2Cl_2 as solvent at 1 mL/min).

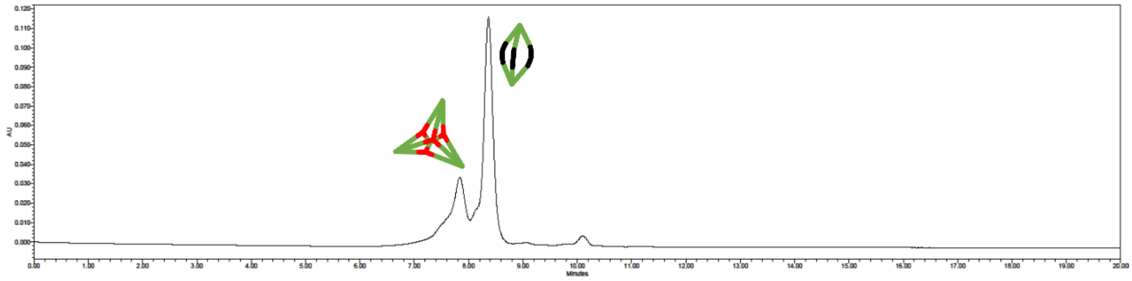

Fig. S69: GPC trace of the assembly of cage **BP1** at t = 48 hours (Phenogel™ 5µm 500 Å, 300 x 7.8 mm, CH_2Cl_2 as solvent at 1 mL/min).

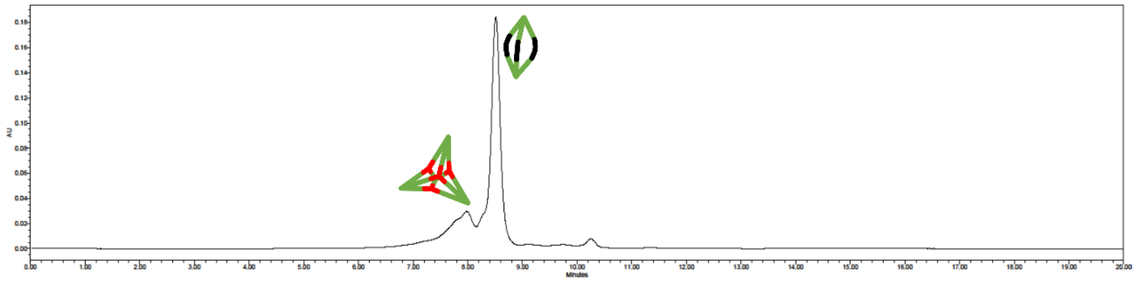

Fig. S70: GPC trace of the assembly of cage **BP1** at t = 72 hours (Phenogel™ 5µm 500 Å, 300 x 7.8 mm, CH_2Cl_2 as solvent at 1 mL/min).

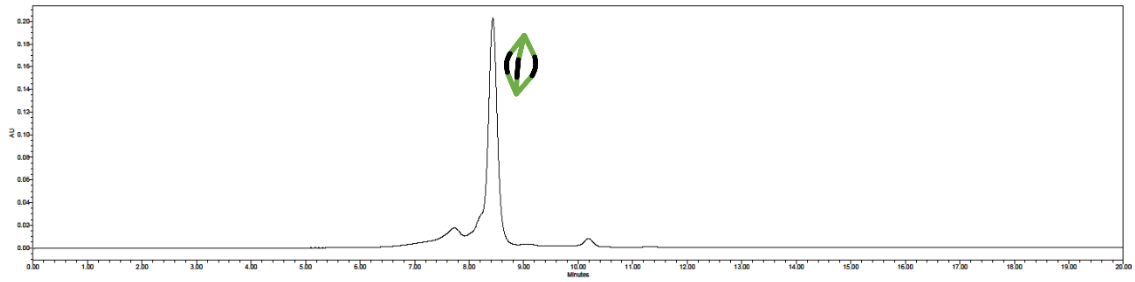

Fig. S71: GPC trace of the assembly of cage **BP1** at t = 96 hours (Phenogel™ 5µm 500 Å, 300 x 7.8 mm, CH_2Cl_2 as solvent at 1 mL/min).

Boronate cage BP2. General procedure for the formation of boronate cages was applied to triboronic acid precursor **11** (5.8 mg, 5.22 µmol, 1.0 equiv), benzene-1,2,4,5-tetraol

(**THB**) (1.11 mg, 7.83 µmol, 1.5 equiv) using 1.0 mL anhydrous $CDCl_3$ (0.005 M) with H_2O (0.6 µL, 31.3 µmol, 6.0 equiv).

HRMS (MALDI+, DCTB, AgTFA) *m/z* calcd. for $C_{162}H_{132}{}^{10}B^{11}B_5O_{18}{}^{109}Ag$ $[M+Ag]^+$ 2538.9056, found 2538.9050.

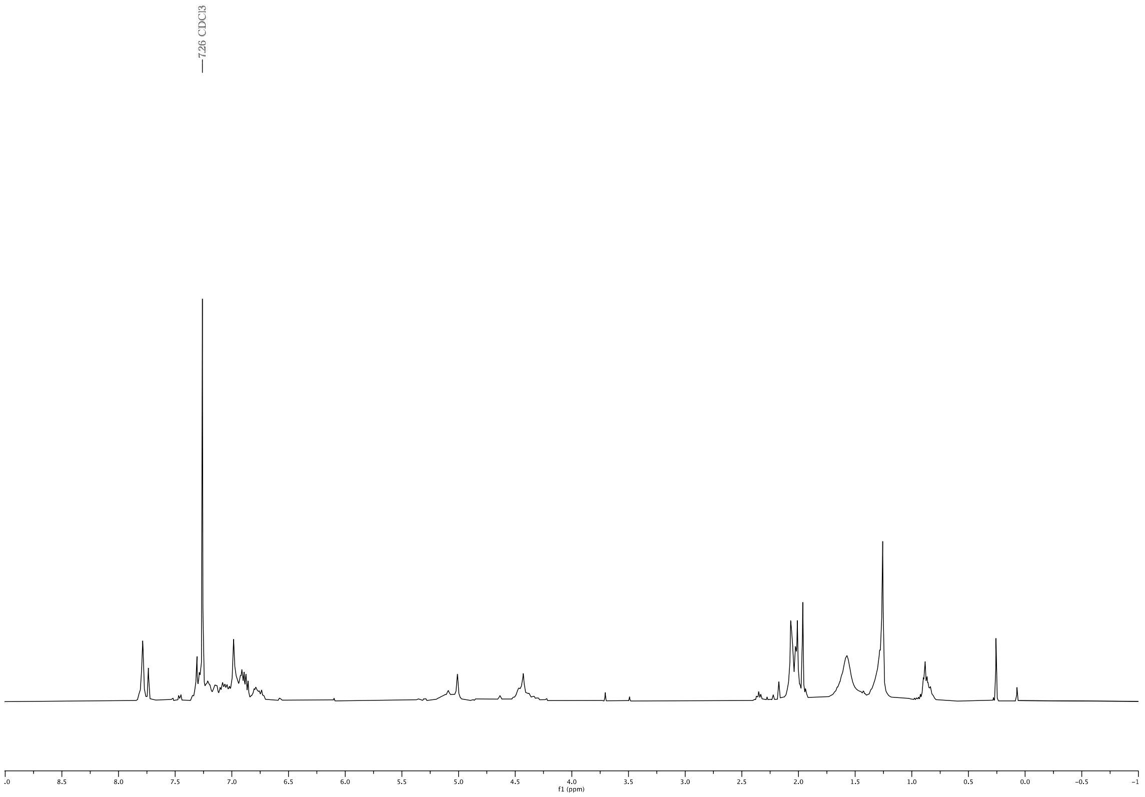

Fig. S72: ^{1}H-NMR (400 MHz, $CDCl_3$) spectrum of the assembly of cage **BP2** at 72 hours, recorded at 25 °C.

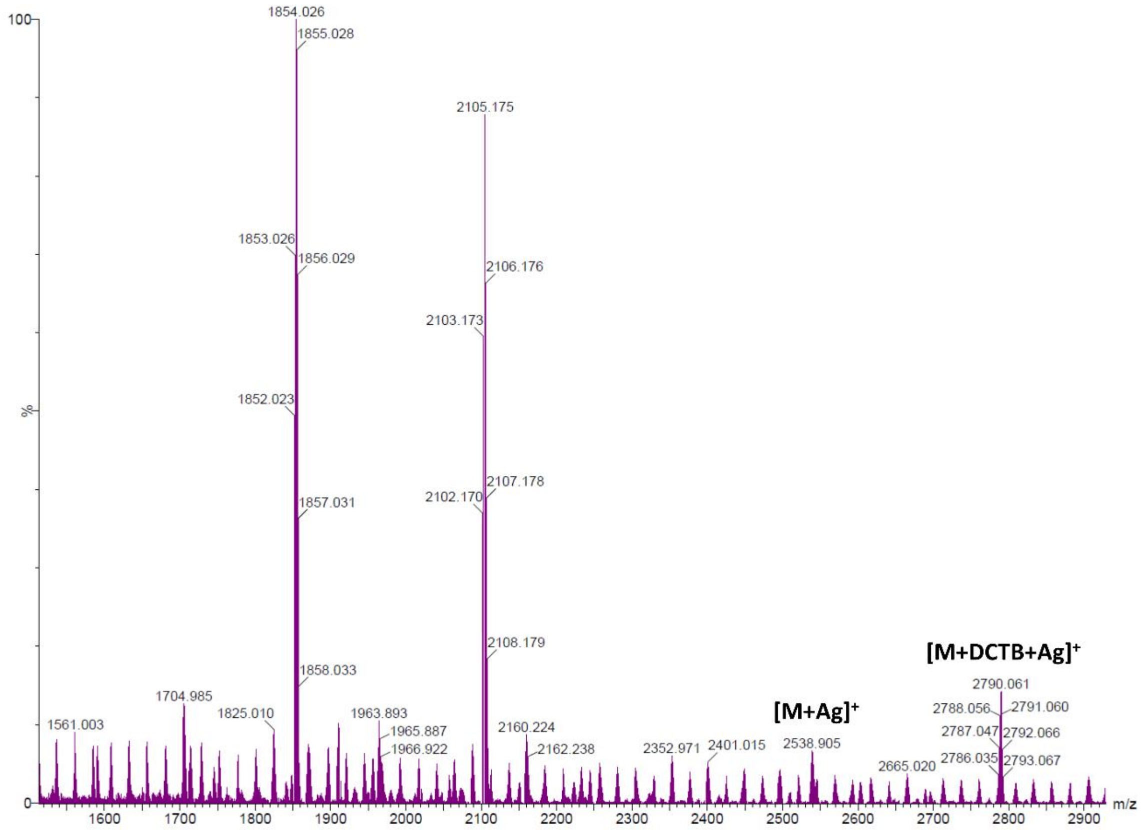

Fig. S73: HR-MALDI-TOF-MS spectrum of boronate cage **BP2** at 72 hours.

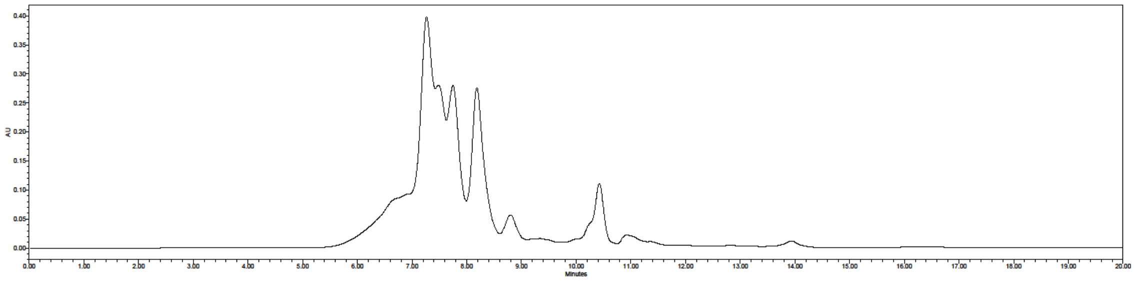

Fig. S74: GPC trace of the assembly of cage **BP2** at t = 72 hours (Phenogel^{TM} 5µm 500 Å, 300 x 7.8 mm, CH_2Cl_2 as solvent at 1 mL/min).

7. Cage Metamorphosis

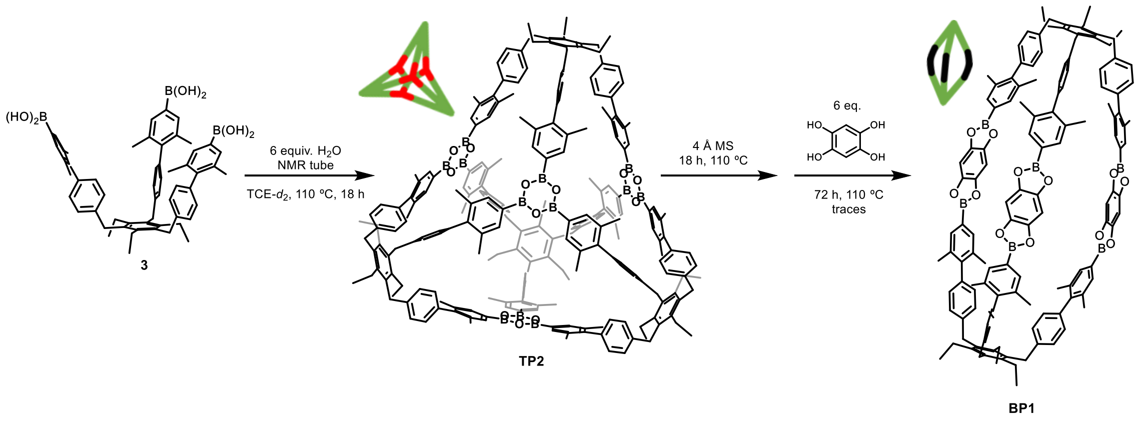

"Dry" Metamorphosis of TP2 to BP1. An NMR tube was charged with triboronic acid **3** (3.3 mg, 3.76 µmol, 1.0 equiv), H_2O (400 µL, 5.65 µmol, 6 equiv), and anhydrous TCE-d_2 (0.7 mL, 0.005 M). The NMR tube was sealed with a Teflon cap, and heated to T = 110 °C overnight. Once the formation of **TP2** was confirmed, two spatula tips of 4 Å MS were added, and the resulting mixture was heated to T = 110 °C overnight. A ^{1}H-NMR spectrum was recorded, and **THB** (0.8 mg, 5.65 µmol, 1.5 equiv relative to **3**) was added. The mixture was heated to T = 110 °C and monitored via ^{1}H-NMR. After 72 h, only traces of **BP1** were detected. Additional time did not increase the yield further.

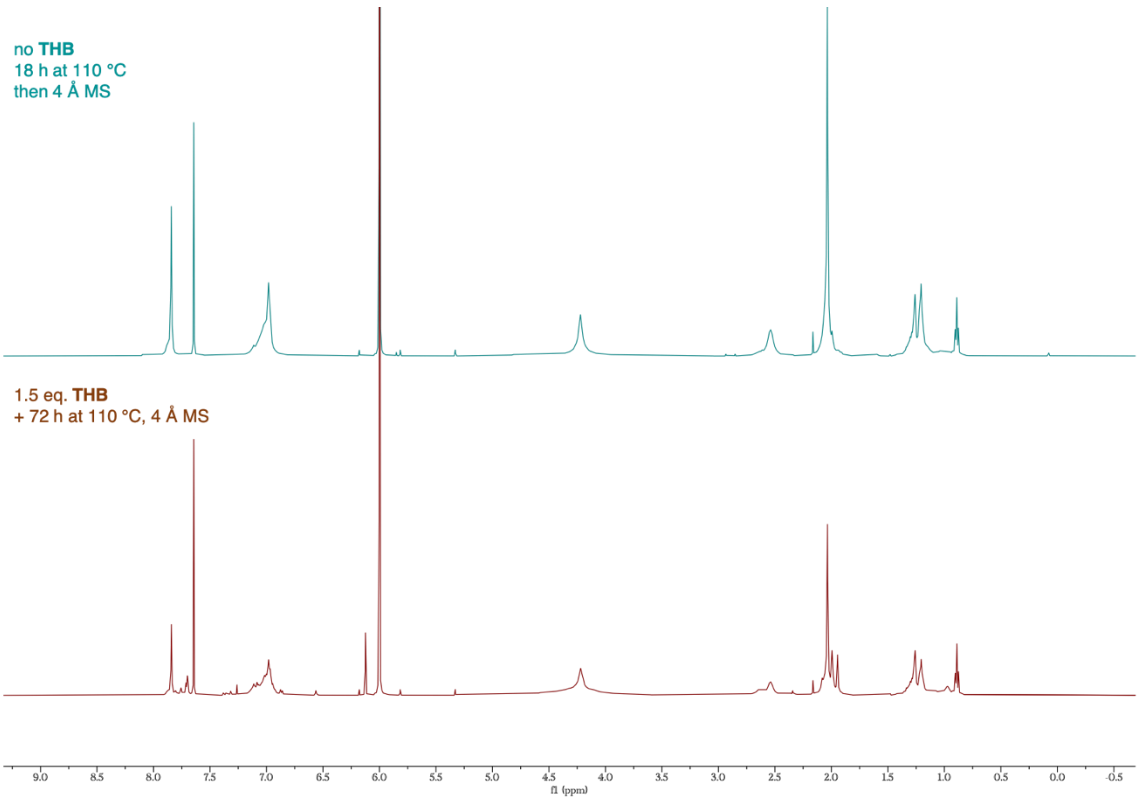

Fig. S75: ^{1}H-NMR spectra for "dry" cage metamorphosis experiments. Spectrum of **TP2** after addition of 4 Å MS and 18 h at 110 °C (top) and after addition of 1.5 equiv THB and heating for 72 h (bottom).

Table S5: Yield determination for "dry" metamorphosis experiment based on quantitative ^{1}H-NMR measurements using TBB as the internal standard. The yield was determined based on the reaction control 72 h after the addition of 1.5 equiv **THB**.

cage	Signal [ppm]	Integral (I)	Nuclei (N)	I/N	n_{cage}[1] [mmol]	$n_{100\%}$[2] [mmol]	y [%]	ΔY [%]
**TP2**	7.8	1.1	24	0.04583	0.0002849	0.000940	30	
	7.0	3.44	48	0.07167	0.0004455	0.000940	47	
	2.0	3.47	72	0.04819	0.0002996	0.000940	32	
						**Y:**	**37**	**10**
**BP1**	0.7	0.13	12	0.01083	0.0000673	0.001880	4	
	2.2	0.62	36	0.01722	0.0001071	0.001880	6	
						**Y:**	**5**	**1**
**?**	2.0	0.91	72	0.01264	0.0000786	0.000940	8	
						**Y:**	**8**	

[1] n_{cage} is calculated by multiplying the moles of added TBB by the I/N ration and defines the moles of cage in the NMR tube. [2] $n_{100\%}$ is the moles of cage for a theoretical yield of 100%.

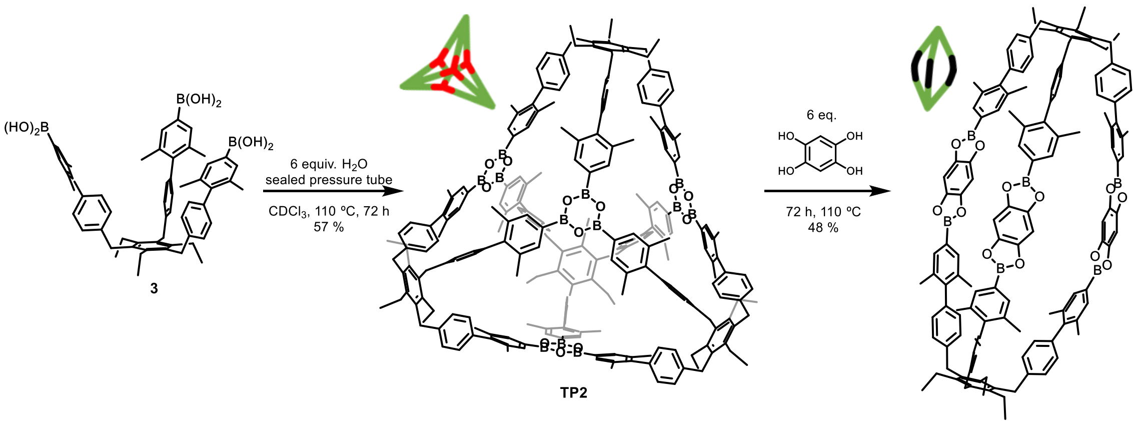

"Wet" Metamorphosis of TP2 to BP1. General procedure for the formation of boroxine cages was applied to triboronic acid precursor **3** (8.77 mg, 10.0 µmol, 1.0 equiv) using 2.0 mL anhydrous $CDCl_3$ (0.005 M) with H_2O (1.1 µL, 60.0 µmol, 6.0 equiv). The yield (Y = 56 ± 7 %) was determined using quantitative ^{1}H-NMR after 72 h by extracting a 0.2 mL aliquot, adding 0.3 mL anhydrous $CDCl_3$ and TCE as the internal standard. Additionally an analytic GPC was performed. This point was defined as t = 0 h.

Subsequently, **THB** (1.92 mg, 13.5 µmol, 1.5 equiv relative to **3**) and 0.4 mL anhydrous $CDCl_3$ were added. The pressure vessel was agitated vigorously to ensure suspension of all material and heated to 110 °C. Reaction control was performed as described above using qNMR and GPC at t = 24 hours, t = 48 hours, t = 72 hours and t = 96 hours. At t = 96 hours, no **TP2** was observed anymore and **BP1** was the predominant species with a yield of 49 ± 9 %.

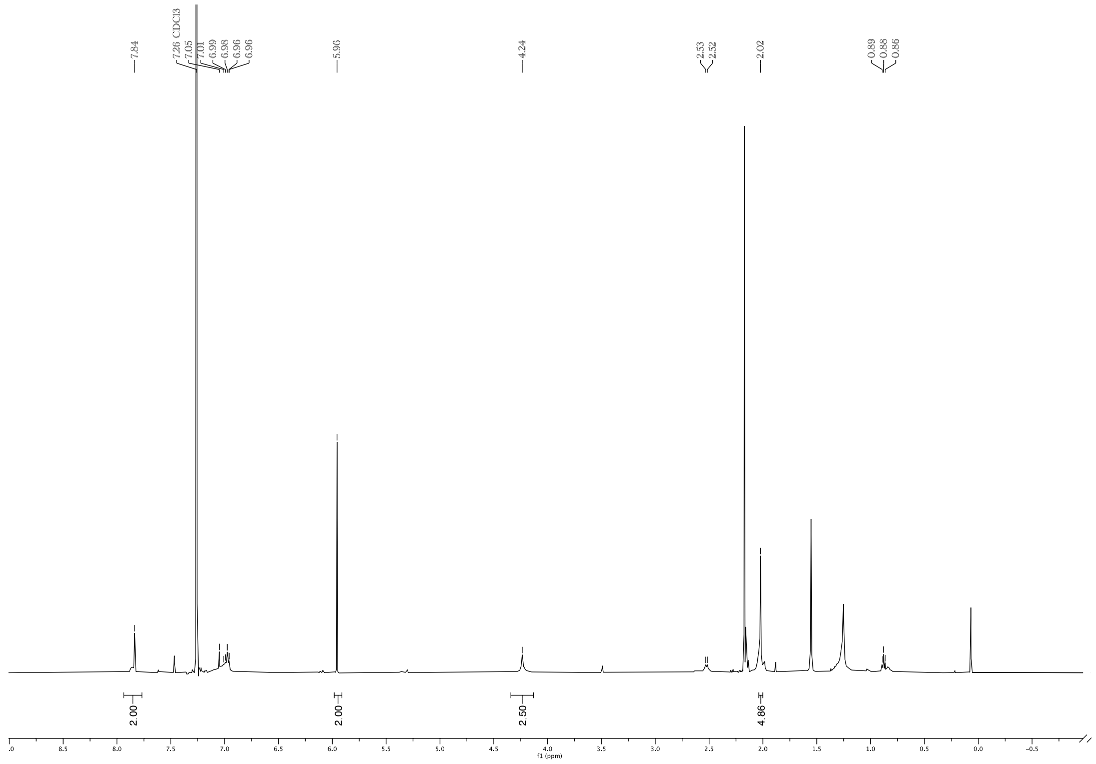

Fig. S76: Reaction control of “wet” cage metamorphosis. Quantitative ^{1}H-NMR spectrum for the reversible formation of **TP2** (72 h, 110 °C, $CDCl_3$). Subsequent to this spectrum, **THB** was added t = 0 hours. TCE used as the internal standard.

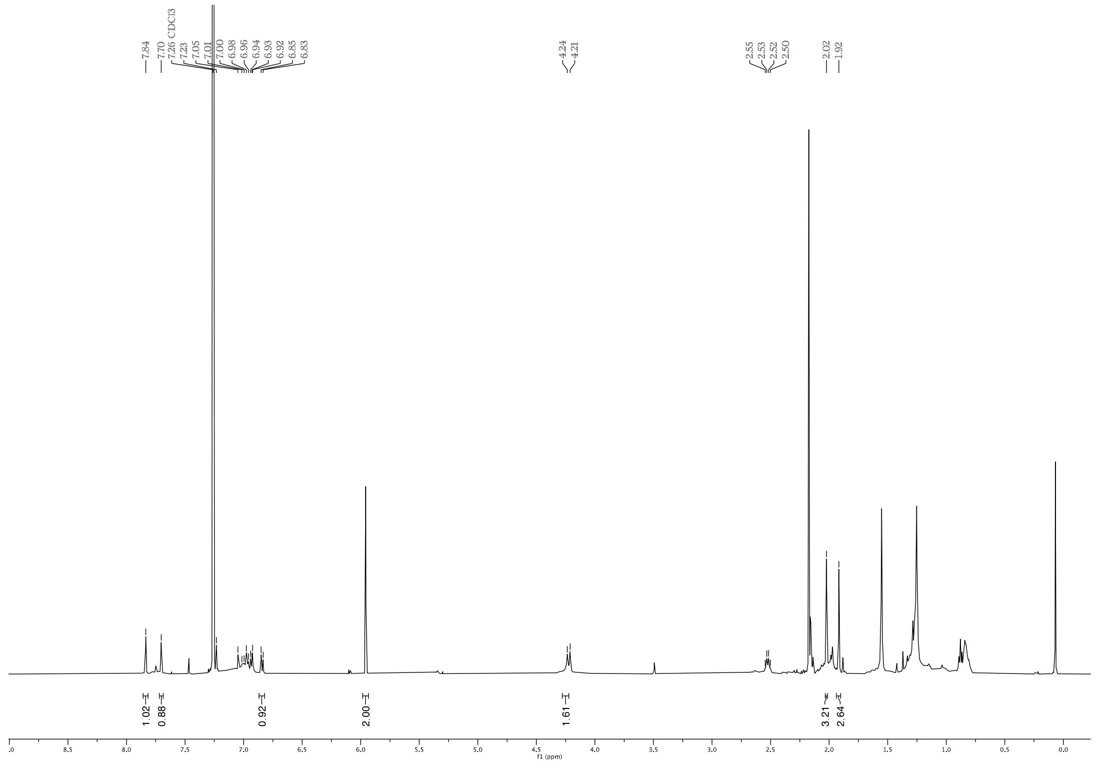

Fig. S77: Reaction control of “wet” cage metamorphosis. Quantitative ^{1}H-NMR spectrum at t = 24 hours. TCE used as the internal standard.

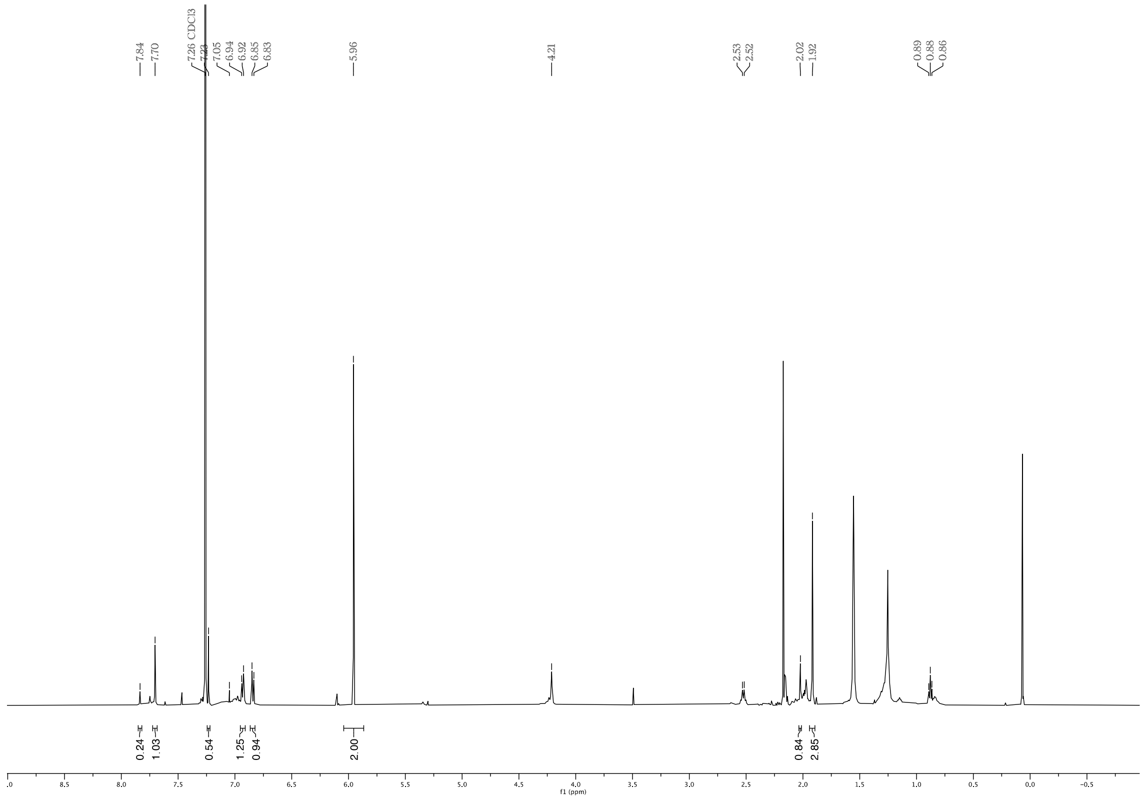

Fig. S78: Reaction control of "wet" cage metamorphosis. Quantitative ^{1}H-NMR spectrum at t = 48 hours. TCE used as the internal standard.

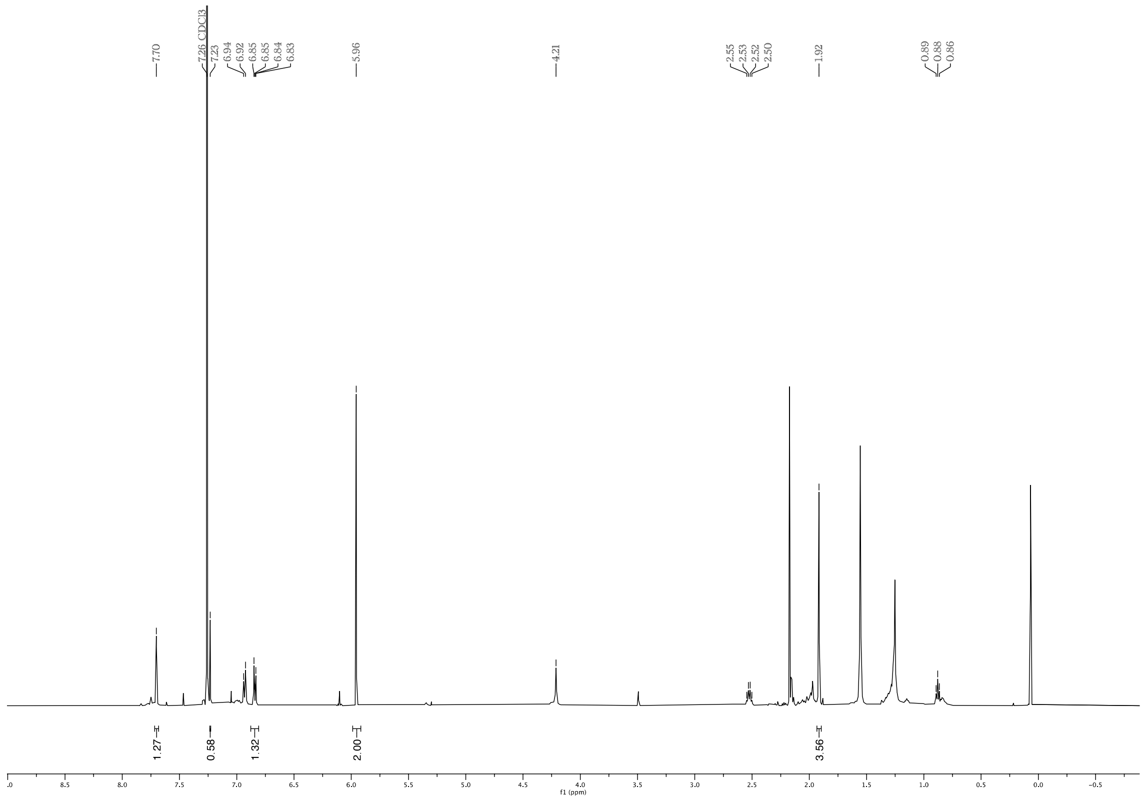

Fig. S79: Reaction control of "wet" cage metamorphosis. Quantitative ^{1}H-NMR spectrum at t = 72 hours. TCE used as the internal standard.

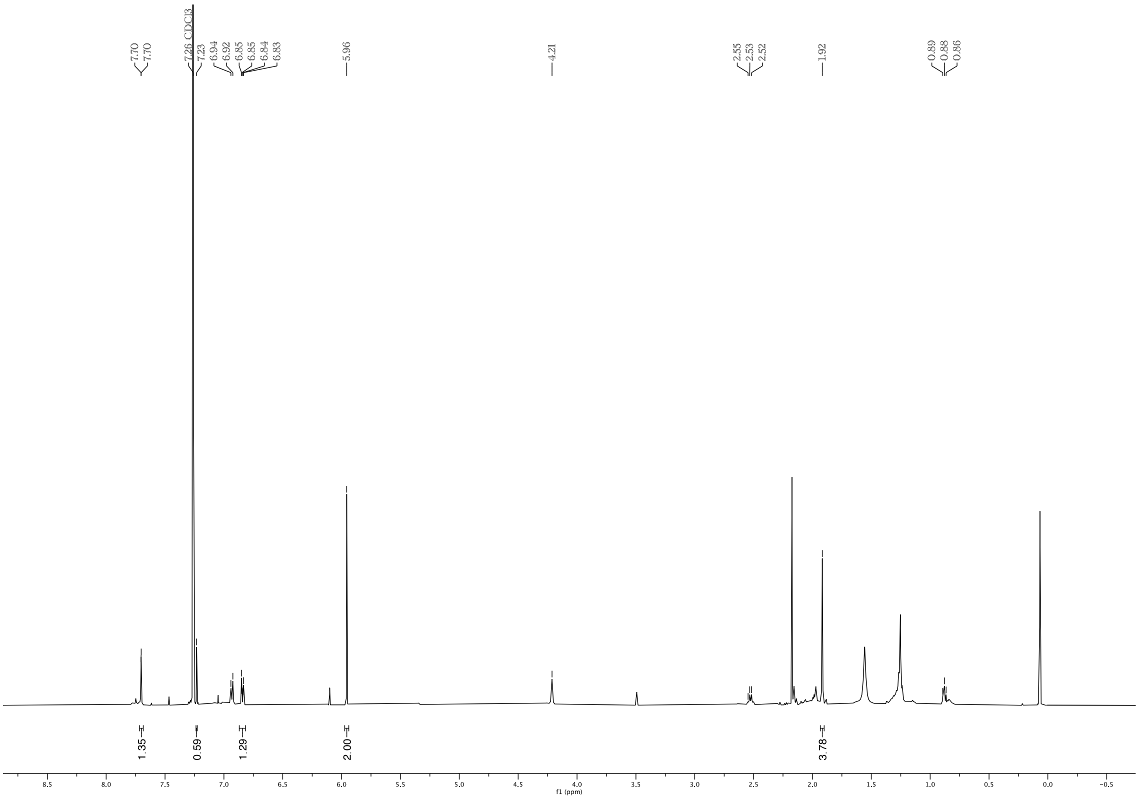

Fig. S80: Reaction control of "wet" cage metamorphosis. Quantitative ^{1}H-NMR spectrum at t = 96 hours. TCE used as the internal standard.

Table S6: Yield determination for "wet" metamorphosis experiment based on quantitative ^{1}H-NMR measurements using TCE as the internal standard.

t = 0 h	Signal [ppm]	Integral (I)	Nuclei (N)	I/N	$n_{aliquot}$[1] [mmol]	n_{tot}[2] [mmol]	y [%]	ΔY [%]
**TP2**	7.8	2.03	24	0.08458	0.0001602	0.00160249	64	
	2.5	1.71	24	0.07125	0.0001350	0.00134988	54	
	2.0	4.86	72	0.06750	0.0001279	0.00127884	51	
						**Y:**	**56**	**7**
**t = 24 h**								
**TP2**	7.8	1.02	24	0.04250	0.0000805	0.00088571	39	
	2.0	3.21	72	0.04458	0.0000845	0.00092913	41	
						**Y:**	**40**	**4**
**BP1**	7.7	0.88	12	0.07333	0.0001389	0.00152829	34	
	6.9	0.92	12	0.07667	0.0001453	0.00159776	36	
	1.8	2.64	36	0.07333	0.0001389	0.00152829	34	
						**Y:**	**34**	**1**
**t = 48 h**								
**TP2**	7.8	0.24	24	0.01000	0.0000189	0.0002084	9	
	2.0	0.84	72	0.01167	0.0000221	0.00024314	11	
						**Y:**	**10**	**1**
**BP1**	7.7	1.03	12	0.08583	0.0001626	0.00178879	40	
	6.9	0.94	12	0.07833	0.0001484	0.00163249	36	
	1.8	2.85	36	0.07917	0.0001500	0.00164986	37	
						**Y:**	**38**	**2**

**t = 72 h**								
**TP2**	7.8	0	24	0	0	0	0	
	2.0	0	72	0	0	0	0	
						**Y:**	**0**	**0**
**BP1**	7.7	1.27	12	0.10583	0.0002005	0.0022056	49	
	7.3	0.58	6	0.09667	0.0001831	0.00201456	45	
	6.9	1.32	12	0.11000	0.0002084	0.00229243	51	
	1.8	3.56	36	0.09889	0.0001874	0.00206087	46	
						**Y:**	**48**	**3**
**t = 96 h**								
**TP2**	7.8	0	24	0	0	0	0	
	2.0	0	72	0	0	0	0	
						**Y:**	**0**	**0**
**BP1**	7.7	1.35	12	0.11250	0.0002131	0.00234453	52	
	7.3	0.59	6	0.09833	0.0001863	0.00204929	46	
	6.9	1.29	12	0.10750	0.0002037	0.00224033	50	
	1.8	3.78	36	0.10500	0.0001989	0.00218823	49	
						**Y:**	**49**	**3**

[1]$n_{aliquot}$ is calculated by multiplying the moles of added TCE by the I/N ration and defines the moles of product in the 0.2 mL aliquot used for the quantification. [2]n_{tot} defines the moles of the product in the whole reaction mixture.

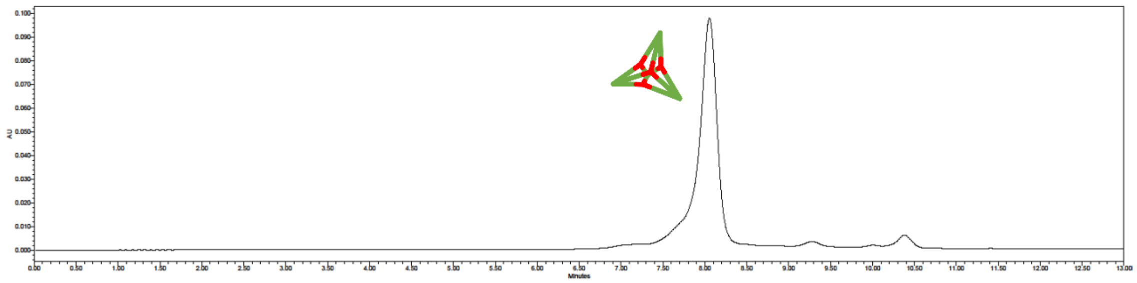

Fig. S81: GPC trace for cage metamorphosis at t = 0 hours (Phenogel™ 5µm 500 Å, 300 x 7.8 mm, CH_2Cl_2 as solvent at 1 mL/min).

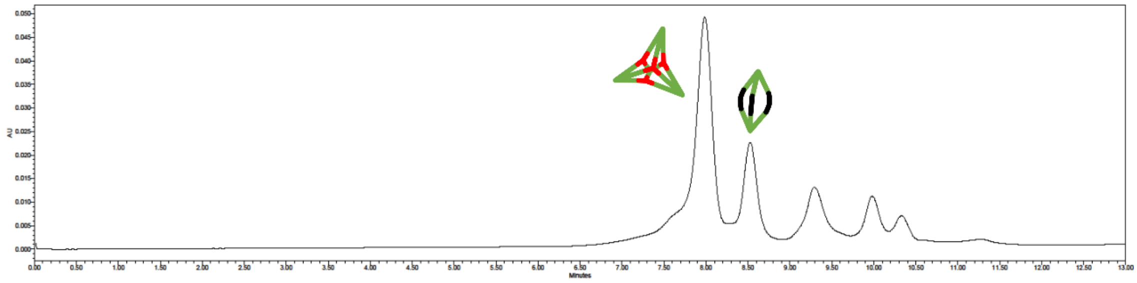

Fig. S82: GPC trace for cage metamorphosis at t = 24 hours (Phenogel™ 5µm 500 Å, 300 x 7.8 mm, CH_2Cl_2 as solvent at 1 mL/min).

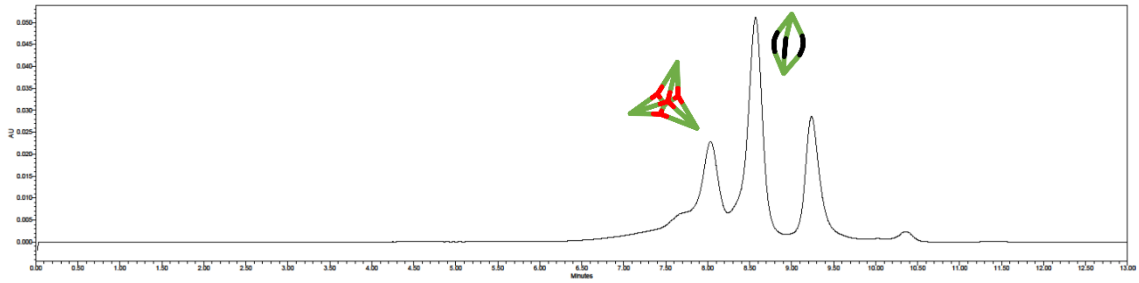

Fig. S83: GPC trace for cage metamorphosis at t = 48 hours (PhenogelTM 5µm 500 Å, 300 x 7.8 mm, CH_2Cl_2 as solvent at 1 mL/min).

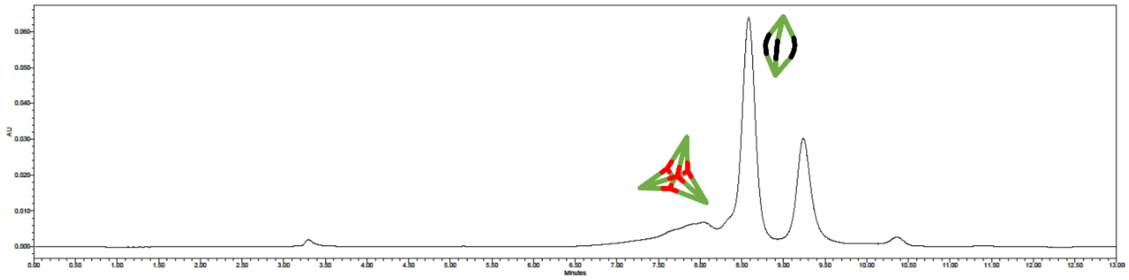

Fig. S84: GPC trace for cage metamorphosis at t = 72 hours (PhenogelTM 5µm 500 Å, 300 x 7.8 mm, CH_2Cl_2 as solvent at 1 mL/min).

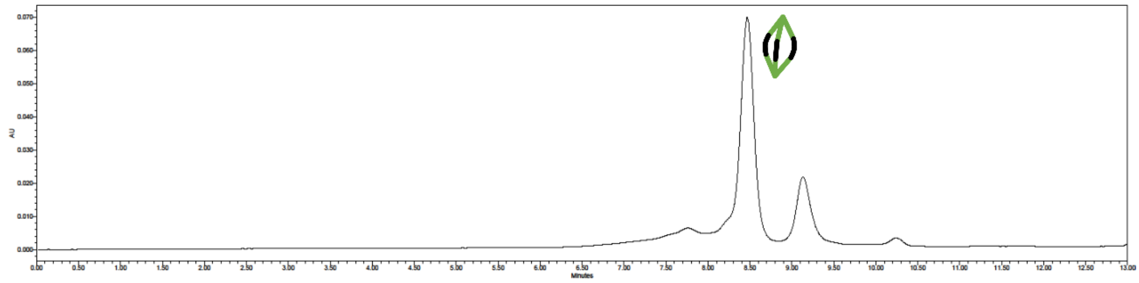

Fig. S85: GPC trace for cage metamorphosis at t = 96 hours (PhenogelTM 5µm 500 Å, 300 x 7.8 mm, CH_2Cl_2 as solvent at 1 mL/min).

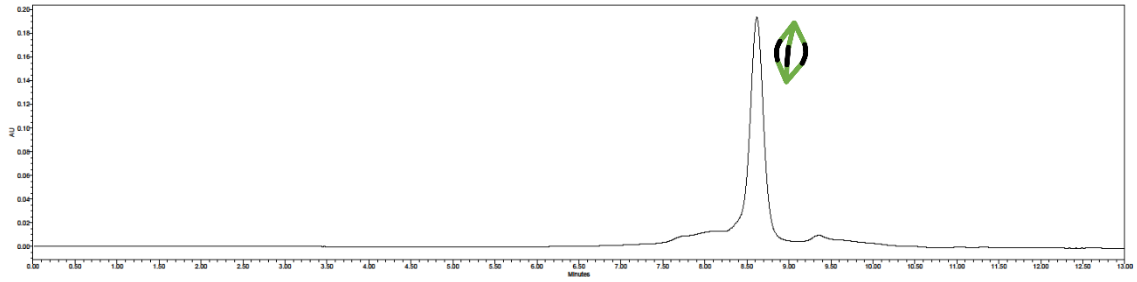

Fig. S86: GPC trace for cage **BP1** (PhenogelTM 5µm 500 Å, 300 x 7.8 mm, CH_2Cl_2 as solvent at 1 mL/min).

8. DOSY-NMR spectra and Volume Approximation

Due to low the solubility and the possibility to form boroxine aggregates of the boronic acid tripods in $CDCl_3$, their respective B-protected precursors were used. To determine the final diffusion coefficient D, only signals that correspond to the species in question were considered. Additionally, in several cases some product signals were not considered when there was ^{1}H-NMR chemical shift overlapping with others signals that do not belong to the studied product (such as water or grease). D values obtained from averaging all molecule signals were used for further calculation of radius and volumes. The volume was determined using the Stokes-Einstein equation and assuming a spherical particle shape:

$$D = \frac{k_B T}{6\pi\eta r}$$

The molecular radius was calculated by using:

$$r = \frac{k_B T}{6\pi\eta}\frac{1}{D}$$

And the molecular volume by using:

$$V = \frac{4}{3}\pi r^3$$

With
r: radius of the sphere particle
V: volume of the sphere particle
D: diffusion coefficient; [D] = m^2/s
k_B: Boltzmann constant, $k_B = 1.38x10^{-23}$ J/K
T: temperature; T = 298 K
η: dynamic viscosity; η ($CDCl_3$) = $5.28x10^{-4}$ Ns/m^2

There are two main different error sources in the calculation of the D values. One derives from the linear regression used to determine D from the area below the signal (area integration) decay of the DOSY measurement, which lies in the order of magnitude 10^{-13} m^2/sec. The other, derives from averaging the different values for D (one for each signal of the molecule) using the standard deviation function "STDEV.S" of Microsoft Excel and lies in the order of magnitude 10^{-12} m^2/sec. As the second error is remarkably bigger than the error from the linear regression, only the latter is considered and defined as ΔD. As the radius r and volume V are functions of D, there errors can be calculated using error propagation as follows:

$$\Delta r(D) = \left|\frac{\partial r}{\partial D}\right| \Delta D = \frac{k_B T}{6\pi\eta D^2}\Delta D$$

and

$$\Delta V(r) = \left|\frac{\partial V}{\partial r}\right| \Delta r = 4r^2\pi\, \Delta r$$

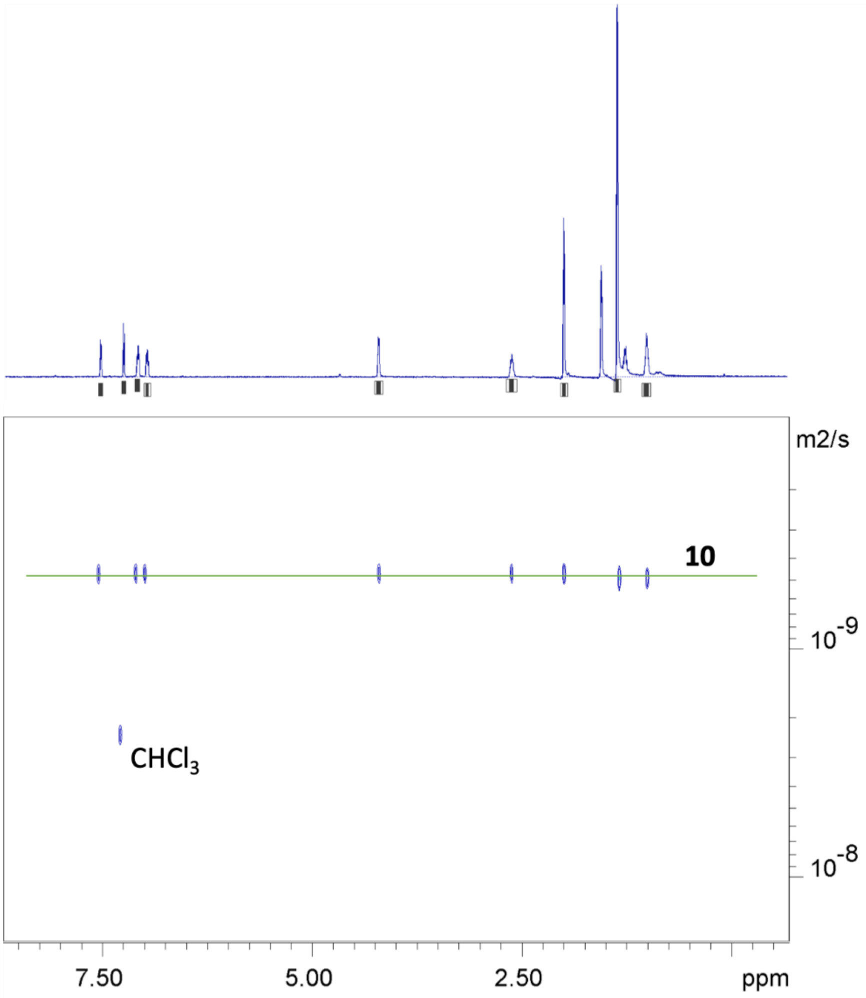

Fig. S87: ^{1}H-DOSY NMR spectrum of building block **10** (500 MHz, $CDCl_3$).

Table S7: Diffusion constants and chemical shifts for each ^{1}H-NMR DOSY signal corresponding to building block **10**.

Building Block 10	Chemical shift [ppm]	D [m²/s]
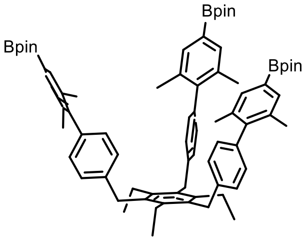 10	7.53	4.68×10^{-10}
	7.10	4.76×10^{-10}
	6.97	4.75×10^{-10}
	4.18	4.72×10^{-10}
	2.62	4.61×10^{-10}
	0.99	4.85×10^{-10}
	**Average:**	$\mathbf{4.73 \times 10^{-10}}$
	**Error ΔD:**	$\mathbf{7.48 \times 10^{-12}}$

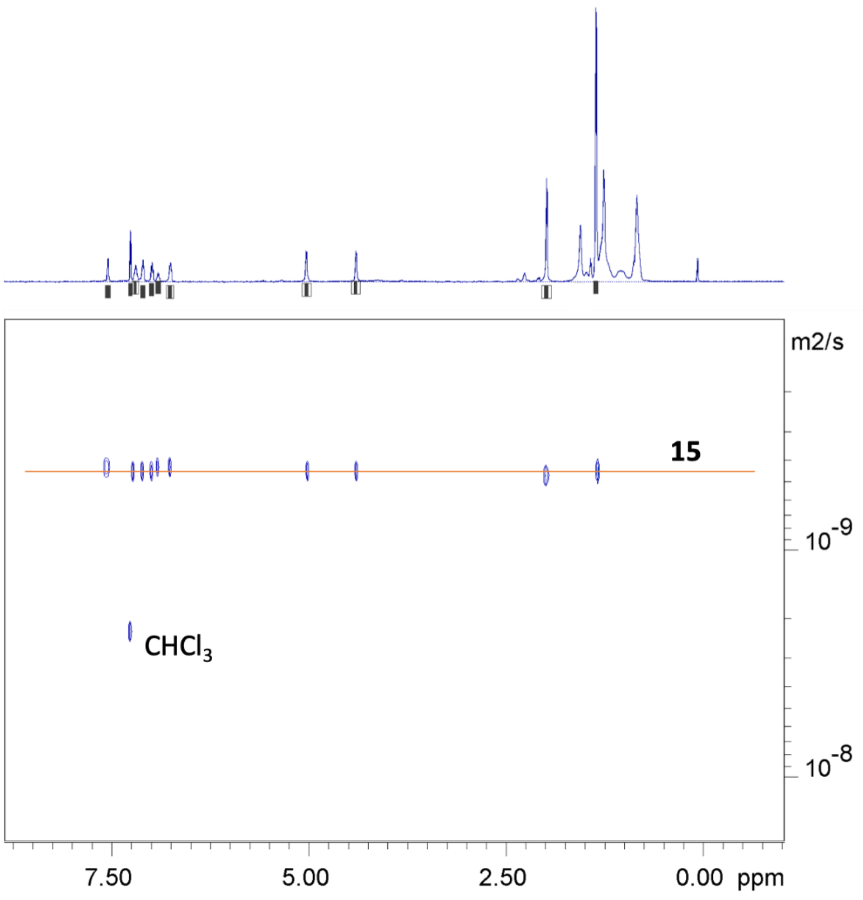

Fig. S88: ^{1}H-DOSY NMR spectrum of building block **15** (500 MHz, $CDCl_3$).

Table S8: Diffusion constants and chemical shifts for each ^{1}H-NMR DOSY signal corresponding to building block **15**.

**Building Block 15**	**Chemical shift [ppm]**	**D [m²/s]**
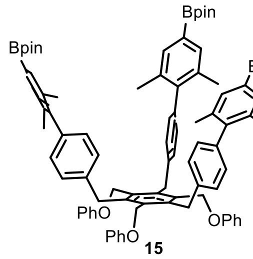 15	7.54	4.40×10^{-10}
	7.20	4.56×10^{-10}
	7.10	4.45×10^{-10}
	6.99	4.44×10^{-10}
	6.91	4.36×10^{-10}
	6.76	4.36×10^{-10}
	5.03	4.55×10^{-10}
	4.38	4.50×10^{-10}
	1.98	4.55×10^{-10}
	1.35	4.57×10^{-10}
	**Average:**	$\mathbf{4.47 \times 10^{-10}}$
	**Error ΔD:**	$\mathbf{8.30 \times 10^{-12}}$

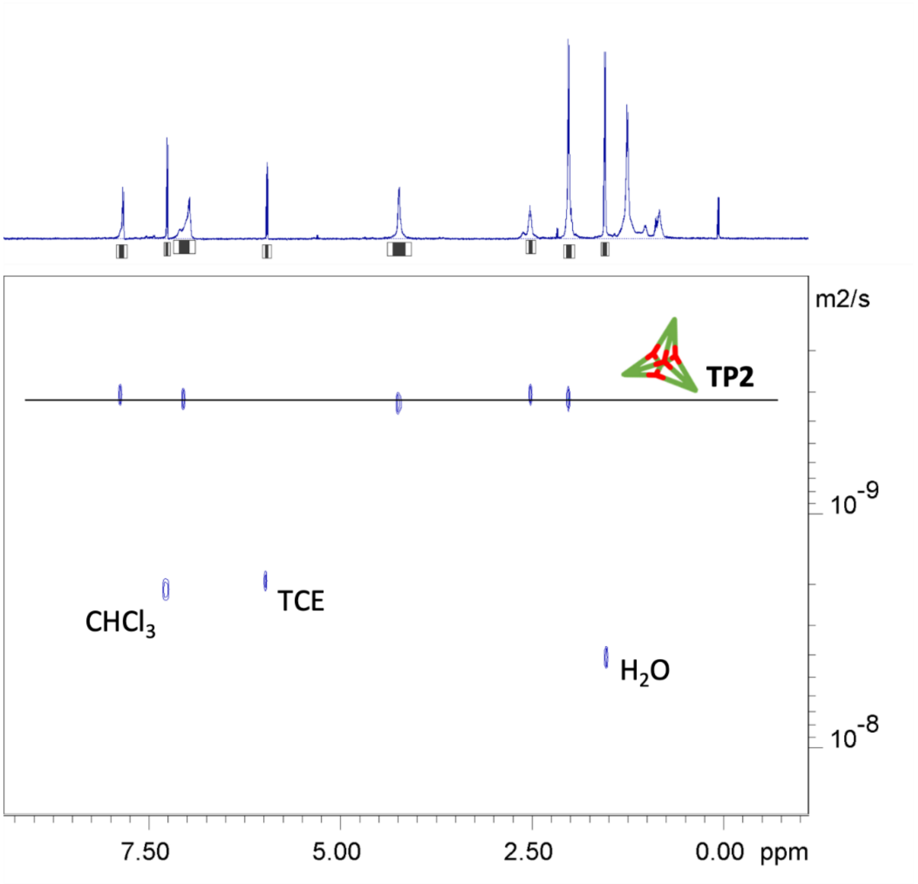

Fig. S89: ^{1}H-DOSY NMR spectrum of cage **TP2** (500 MHz, $CDCl_3$).

Table S9: Diffusion constants and chemical shifts for each ^{1}H-NMR DOSY signal corresponding to cage **TP2**.

**TP2**	**Chemical shift [ppm]**	**D [m²/s]**
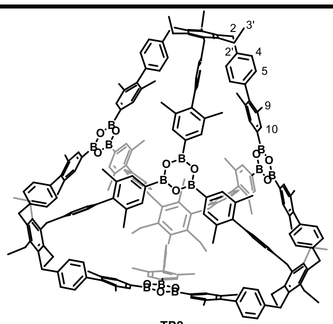 TP2	7.86	3.17×10^{-10}
	7.04	3.26×10^{-10}
	4.23	3.32×10^{-10}
	2.50	3.22×10^{-10}
	2.00	3.30×10^{-10}
	**Average:**	$\mathbf{3.25 \times 10^{-10}}$
	**Error ΔD:**	$\mathbf{6.07 \times 10^{-12}}$

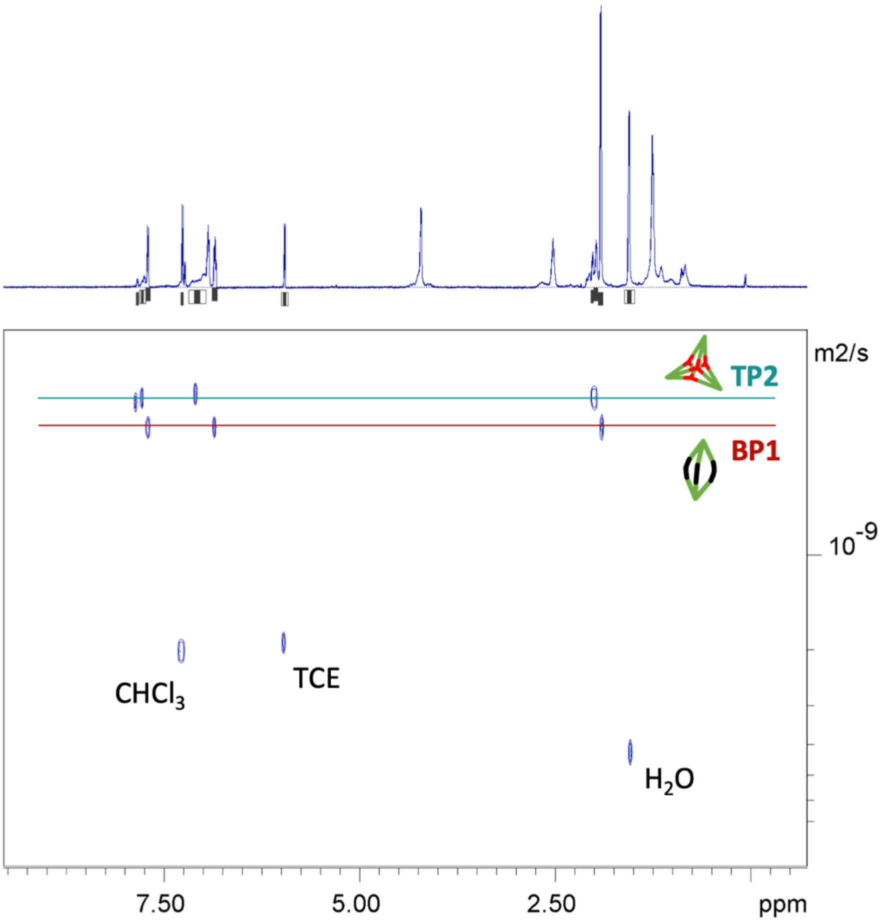

Fig. S90: ^{1}H-DOSY NMR spectrum of the formation of cage **BP1**. The DOSY shows the coexistence of two/three species with distinct diffusion coefficients along with the $CDCl_3$ (rest proton signal), TCE (internal standard) and water (500 MHz, $CDCl_3$).

Table S10: Diffusion constants and chemical shifts for each ^{1}H-NMR DOSY signal corresponding to cage **BP1,** cage **TP2** and unknown species.

species	Chemical shift [ppm]	D [m²/s]
**TP2**	7.83	3.41×10^{-10}
**?**	7.77	3.24×10^{-10}
**BP1**	7.70	4.02×10^{-10}
**TP2**	7.06	3.16×10^{-10}
**BP1**	6.84	3.99×10^{-10}
**TP2**	2.02	3.33×10^{-10}
**BP1**	1.9	3.99×10^{-10}
	**Average TP2/?:**	**3.29×10^{-10}**
	**Error ΔD TP2/?:**	**1.28×10^{-11}**
	**Average BP1:**	**4.00×10^{-10}**
	**Error ΔD BP1:**	**2.12×10^{-12}**

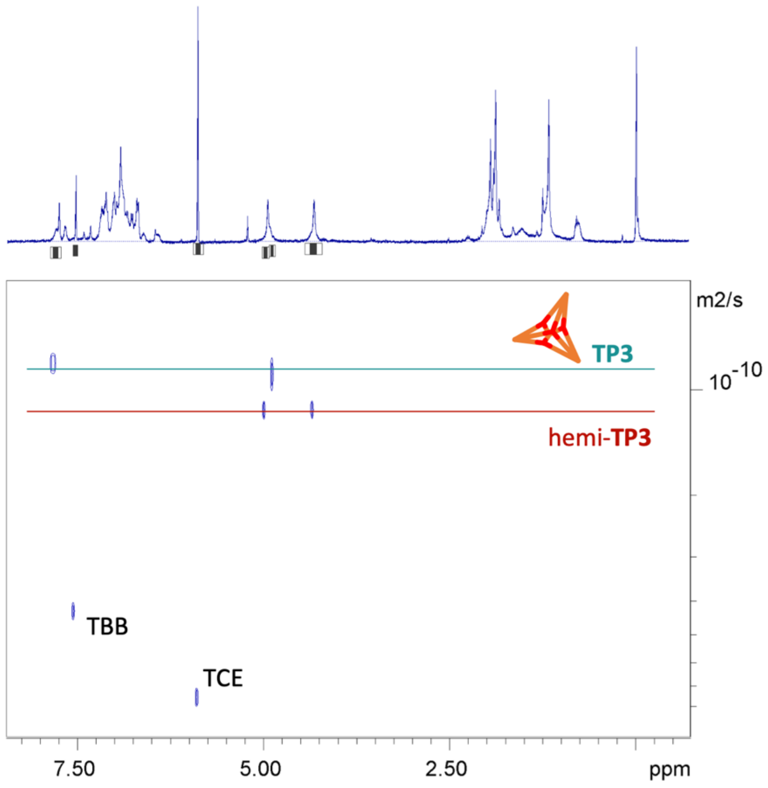

Fig. S91: ^{1}H-DOSY NMR spectrum for the formation of cage **TP3** (500 MHz, TCE-d_2).

Table S11: Diffusion constants and chemical shifts for each ^{1}H-NMR DOSY signal corresponding to cage **TP3,** hemi-**TP3** in TCE-d_2.*

species	Chemical shift [ppm]	D [m²/s]
**TP3**	7.81	8.43×10^{-11}
**hemi-TP3**	4.97	1.14×10^{-10}
**TP3**	4.89	8.43×10^{-11}
**hemi-TP3**	4.33	1.14×10^{-10}
	**Average TP3**	**3.29×10^{-10}**
	**Error ΔD TP3**	**1.28×10^{-11}**
	**Average hemi-TP3:**	**4.00×10^{-10}**
	**Error ΔD hemi-TP3:**	**0**

***hemi-TP3 refers to partially formed **TP3** consisting of two units of monomer **15** condensated together via two partial boroxine rings, leaving two unbound boronic acids.

Table S12: Diffusion coefficients, radii, volumes and corresponding errors.

	D<br>[10^{-10} m^2/s]	ΔD<br>[10^{-10} m^2/s]	r<br>[Å]	Δr<br>[Å]	V<br>[$Å^3$]	ΔV<br>[$Å^3$]
**10**	4.73	0.07	8.74	0.14	2794	132
**15**	4.47	0.08	9.24	0.17	3308	184
**TP2**	3.25	0.06	12.7	0.24	8597	481
**TP2***	3.29	0.13	12.6	0.49	8356	974
**BP1***	4.00	0.02	10.3	0.55	4628	73
**TP3****	0.87	0.04	13.7	0.06	10684	13
**hemi-TP3****	1.14	0	10.4	0	4724	0

*values obtained from mixture of **TP2** and **BP1** (

Table **S10**); **D determined in TCE-d_2 with η (TCE-d_2) = 0.00184 Ns/m^2.

9. Molecular Modeling and Volume Approximation

Molecular models were obtained by molecular mechanics calculations using Schrodinger/Macromodel. To obtain the global minimum energy structure, conformational searches was performed using a hybrid method that included MonteCarlo (MCMM) and Low Mode structure sampling and the OPLS4 force field. Extended nonbonded cutoff distances (a van der Waals cutoff of 8.0 Å and an electrostatic cutoff of 20.0 Å) were used. The lowest-energy structure of the conformational search was used as the final molecular model.

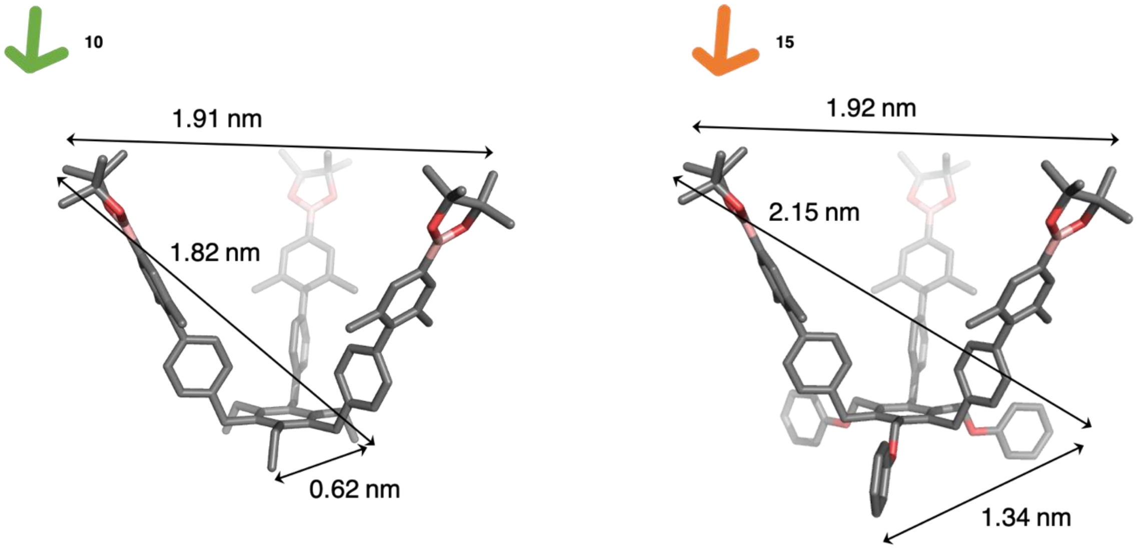

Fig. S92: Molecular models (OPLS4 force field) of B-pin tripods **10** and **15**.

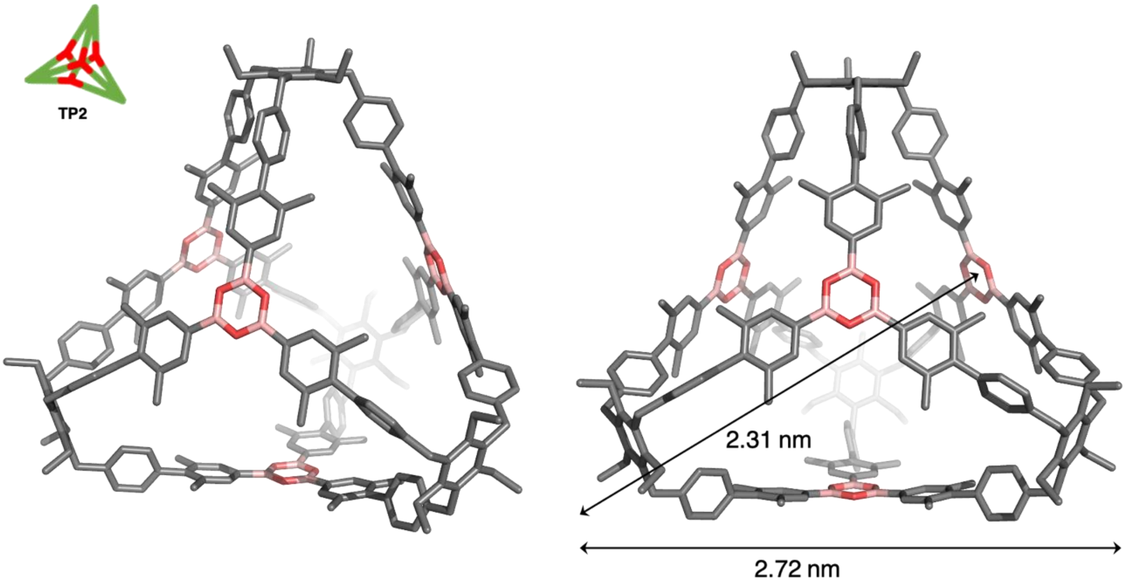

Fig. S93: Molecular model (OPLS4 force field) of cage **TP2**.

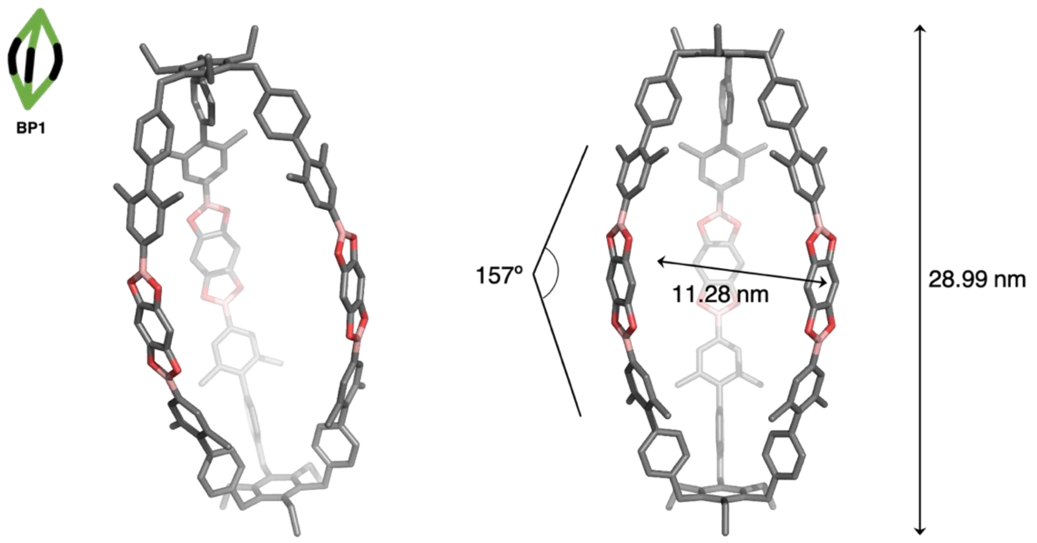

Fig. S94: Molecular model (OPLS4 force field) of boronate cage **BP1**.

Approximate volumes were calculated based on the molecule diameters obtained from the molecular models. Due to the irregular shape of the structures, the diameter was calculated by mean averaging representative diameters for each molecule. For the tripods this means that the Bpin-Bpin, foot-foot and Bpin-foot distances were taken into account. For the tetrapod, the foot-foot and foot-boroxine distance was considered. For the boronate bipyramidoid the foot-foot and THB-centroid diameters were used. The volume was then calculated analogously to DOSY measurements (see Paragraph 8). This constitutes a rough approximation and only serves for qualitative comparison. For the tripodal precursors, the corresponding Bpin-tripods (instead of the boronic acids) were used to ensure comparability with the values obtained from the DOSY-NMR experiments.

Table S13: Overview over volume calculation of tripods **10**, **15** and cages **TP2** and **BP1** on molecular modeling.

compound		d [nm]	r [nm]	V [Å³]
**10**	1	0.62	0.31	
	2	1.82	0.91	
	3	1.91	0.96	
	**average**		**0.73**	**1603**
**15**	1	1.34	0.67	
	2	2.15	1.08	
	3	1.92	0.96	
	**average**		**0.96**	**3083**
**TP2**	1	2.71	1.36	
	2	2.31	1.16	
	**average**		**1.26**	**8310**
**BP1**	1	2.90	1.45	
	2	1.13	0.56	
	**average**		**1.01**	**4274**

10. References

[1] F. E. Golling, M. Quernheim, M. Wagner, T. Nishiuchi and K. Müllen, *Angew. Chem. Int. Ed.*, 2014, **53**, 1525.

[2] M. Rondelli, A. H. Daranas and T. Martín *J. Org. Chem*., 2023, **88**, 2113.

[3] A. M. Haydl, L. J. Hilpert and B. Breit, *Chem. Eur. J.*, 2016, **22**, 6547.

[4] O. R. Cromwell, J. Chung and Z. Guan, *J. Am. Chem. Soc.*, 2015*,* **137**, 6492.

[5] The ^{13}C-signal corresponding to Ar-C-Bpin was too weak to be detected. The aliphatic region shows three additional signals, probably due to the presence of slow interconversion of conformers. The signals could be easily assigned by comparison with DEPT experiments.

[6] DCTB = *trans*-2-[3-(4-*tert*-Butylphenyl)-2-methyl-2-propenylidene]malononitrile, matrix used in sample preparation for MALDI analysis.
